# Toward N-*peri*-Annulated Planar Blatter Radical through aza-Pschorr and Photocyclization

**DOI:** 10.1021/acs.joc.3c02051

**Published:** 2023-11-24

**Authors:** Patrycja Szamweber, Anna Pietrzak, Georgia A. Zissimou, Piotr Kaszyński

**Affiliations:** † Centre of Molecular and Macromolecular Studies, Polish Academy of Sciences, 90-363 Łódź, Poland; ‡ Faculty of Chemistry, Łódź University of Technology, 90-924 Łódź, Poland; § Faculty of Chemistry, University of Łódź, 91-403 Łódź, Poland; ∥ Department of Chemistry, Middle Tennessee State University, Murfreesboro, Tennessee 37132, United States

## Abstract

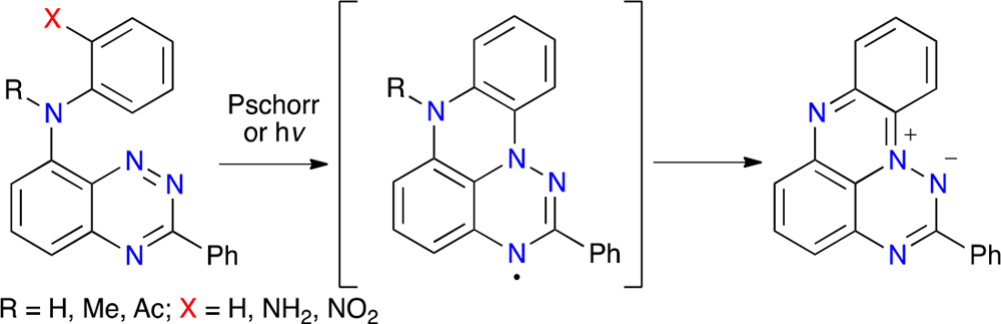

Preparation of the elusive N-*peri*-annulated planar Blatter radicals was attempted using aza-Pschorr and photocyclization methods. In both methods, substrates containing N–Me and N–Ac groups yielded a zwitterionic heterocycle lacking the N-substituent as the main product, while in one of them a carbazole derivative representing a new heterocyclic system was also obtained. The formation of the zwitterion and the carbazole suggests the formation of the desired planar Blatter radical, which undergoes facile fragmentation through homolysis of the N–R bond. This mechanism is supported by DFT computational results, which also suggest that *N*-Ar derivatives should be sufficiently stable for isolation. Electronic structures of three planar Blatter radicals annulated with the O, S, and N–Ph groups are compared.

## Introduction

**Figure 1:**
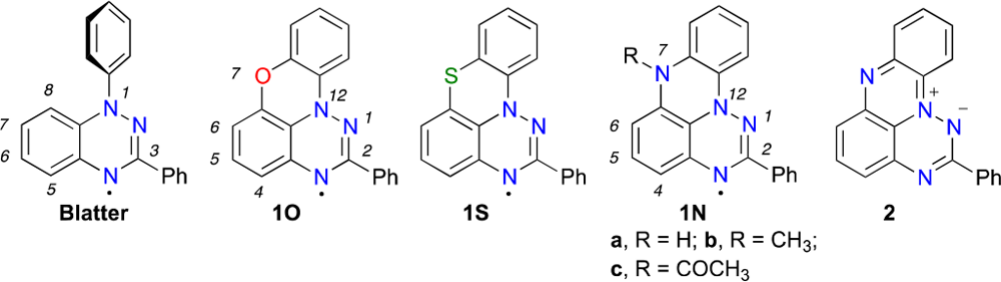
Structures and numbering system of Blatter radical, its planar analogues **1**, and zwitterion **2**.

Benzo[*e*][1,2,4]triazin-4-yls,^1,2^ derivatives of the prototypical Blatter radical^3^ (**Blatter**, Figure 1), are of increasing interest as building blocks and components of modern functional materials,^2^ such as sensors,^4^ metal–organic frameworks,^5^ spin filters,^6,7^ organic batteries,^8,9^ and liquid crystals.^10,11^ For this reason, there is a concerted effort to develop chemistry of this exceptionally stable, π-delocalized, and redox active paramagnetic system. In this context, we have demonstrated planar Blatter radical analogues,^12^ in which positions C(8) and C(ortho) in **Blatter** are connected with an oxygen (**1O**) or sulfur (**1S**) atom (Figure 1). This structural change resulted in a better spin delocalization and a red shift of the electronic absorption spectra^12^ and enabled synthesis of new paramagnetic liquid crystals.^13,14^ Therefore, it is of interest to develop a synthetic route to radicals **1N**, the nitrogen analogues of **1O** and **1S**. In contrast to chalcogens in **1O** and **1S**, the nitrogen atom in **1N** is trivalent and the substituent R connected to the N atom can be used to tune the electronic system (Figure 1).

A recent attempt^15^ at synthesis of the 4-methyl derivative of the parent radical **1N-a** through a double cyclocondensation of a diamide resulted in the 4-methyl derivative of zwitterion **2** apparently through a loss of a hydrogen atom from the N(7) position (Figure 1). Since hydrogen transfer from a spin-containing position is an easy and common process, it could be speculated that substituting the N(7) position with a group, such as methyl (**1N-b**) or acetyl (**1N-c**), could lead to a stable and isolable radical.

Herein we report our efforts at obtaining radicals **1N-a** and **1N-b** by using two recently developed methods for the synthesis of planar Blatter radicals and appropriate substrates. Results of the reactions, including formation of a new heterocyclic ring system, were used to formulate reaction mechanisms supported with extensive DFT calculations. Stability of the expected radicals **1N** as well as comparison of the electronic structures of planar Blatter radicals **1** are assessed using DFT computational methods.

### Synthetic Strategy

**Figure 2:**
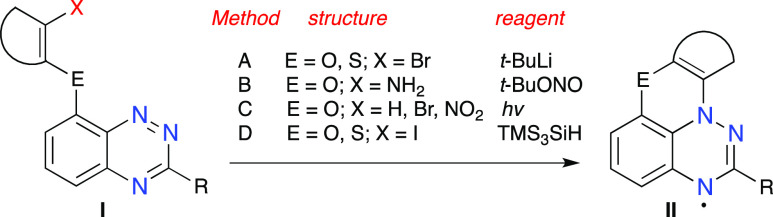
Methods for accessing planar Blatter radicals **II**.

There are four methods developed so far to access planar Blatter radicals with annulating oxygen^12,16−18^ (E = O) and sulfur^12,18^ (E = S) atoms (Figure 2), which could, in principle, give access to planar nitrogen-annulated radicals **1N**. All methods involve a (het)aryl substituent connected at the C(8) position of the benzo[*e*][1,2,4]triazine ring (**I**, Figure 2) and containing an appropriate functional group X in the adjacent position allowing for the formation of radical **II**. Only in the case of Method C,^17^ photocyclization also occurs for nonfunctionalized (X = H) precursors.

**Figure 3:**
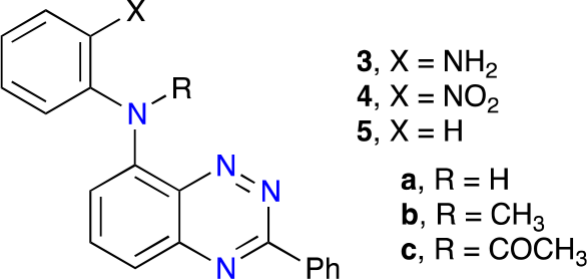
Selected precursors to radicals **1N**.

Analysis of the four methods demonstrated that radicals **1N** could, in principle, be obtained through the aza-Pschorr method^16^ (Method B) using amines **3** (Figure 3) or through photocyclization^17^ (Method C) of derivatives **4** or **5**. The choice of the methods was dictated by the anticipated easier access to derivatives **3**–**5** than to halogen-substituted derivatives required for Methods A and D.

## Results and Discussion

### Synthesis of Precursors

**Scheme 1:**
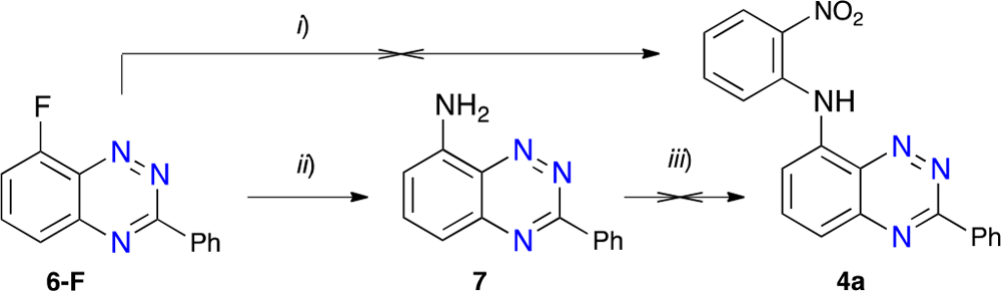
Attempted Preparation of 2-Nitrophenyl Derivative **4a** Reagents and conditions: (*i*) NaH, 2-nitroaniline, dry DMSO, 90 °C; (*ii*) 25% aqueous ammonia, MeCN, 120 °C, sealed tube, 12 h, 90% yield; (*iii*) NaH, 2-fluoronitrobenzene, dry DMSO, 90 °C.

Nitro derivative **4a** was envisioned as the key precursor to derivatives **3** and **4**. Initial attempts were focused on aromatic nucleophilic substitution reactions of the readily available^12^ 8-fluoro-3-phenylbenzo[*e*][1,2,4]triazine (**6-F**) under standard conditions:^12,17^ DMSO solvent and the presence of NaH (Scheme 1). Despite prolonged reaction times (up to 72 h) and temperatures up to 120 °C, the expected product was not formed, and the unreacted starting **6-F** was recovered. Other combinations of aprotic solvents and bases were equally unsuccessful. An attempt to N-arylate 8-amino-3-phenylbenzo[*e*][1,2,4]triazine (**7**) with 2-fluoronitrobenzene in DMSO in the presence of NaH was also unsuccessful. The lack of success in these two processes is presumably due to the low nucleophilicity of both amines. In contrast, the reaction of **6-F** with ammonia readily produced the amine **7** in a high yield (Scheme 1).

**Scheme 2:**
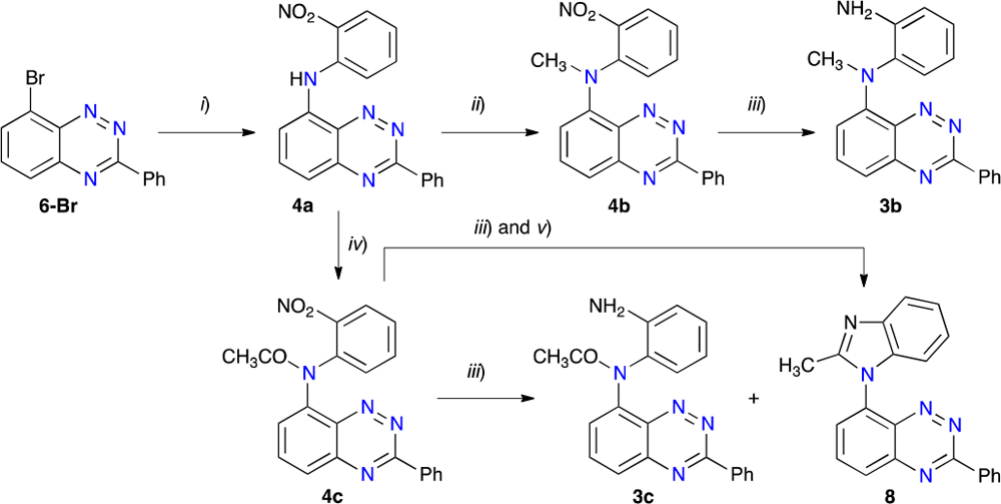
Preparation of Precursors **3** and **4** Reagents and conditions: (*i*) 2-nitroaniline (1.1 equiv), Pd_2_(dba)_3_ (5 mol %), DavePhos (7.5 mol %), Cs_2_CO_3_ (2 equiv), toluene, 120 °C, N_2_, 24 h, 82% yield; (*ii*) MeI (3 equiv), NaH (3 equiv), DMA, N_2_, 2 h, rt, 89% yield; (*iii*) H_2_ (55 psi), EtOH/THF for **4b**, EtOH/CH_2_Cl_2_ for **4c**, Pd/C 10% (30% mass), rt, overnight, **3b** 94% yield, **3c** see text; (*iv*) Ac_2_O (5 equiv), ZnCl_2_ (1 equiv), 80 °C, 28 h, 76% yield; (*v*) EtOH, 1 drop of conc. HCl, 1 h, 95% yield.

As the nucleophilic substitution failed to yield **4a**, attention was turned to metal-catalyzed processes. Initial experiments with an Ullmann-type reaction of 8-bromo-3-phenylbenzo[*e*][1,2,4]triazine (**6-Br**) with 2-nitroaniline in the presence of CuI (20 mol %), 1,10-phenanthroline (5 mol %), and Cs_2_CO_3_ in dry DMF again did not yield the desired product **4a**, even with elongated reaction times (up to 4 days) and temperatures up to 110 °C. Finally, Pd-catalyzed C–N cross coupling of **6-Br** and 2-nitroaniline conducted in toluene in the presence of Pd_2_(dba)_3_, DavePhos and Cs_2_CO_3_ gave the desired product **4a** as red crystals in yields up to 82% (Scheme 2).

**Scheme 3:**
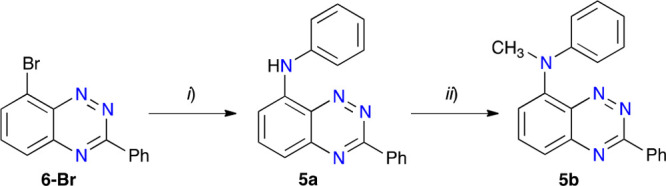
Preparation of Derivatives **5** Reagents and conditions: (*i*) aniline (1.1 equiv), Pd_2_(dba)_3_ (5 mol %), DavePhos (7.5 mol %), Cs_2_CO_3_ (2 equiv), toluene, N_2_, 120 °C, 28 h, 87% yield; (*ii*) MeI (3 equiv), NaH (3 equiv), DMA, N_2_, 2 h, rt, 90% yield.

The obtained nitroaniline **4a** was subsequently converted to the *N*-methyl (**4b**) and *N*-acetyl (**4c**) derivatives using the standard conditions. Thus, a reaction of **4a** with excess MeI in DMA in the presence of NaH gave *N*-Me derivative **4b** in 89% yield, while **4c** was obtained in 76% yield by reaction of **4a** with excess acetic anhydride in the presence of ZnCl_2_ at 80 °C. The reaction flasks were protected from light since the products appeared to be photosensitive. For comparison purposes, compounds **5a** and **5b** were obtained in an analogous way starting from **6-Br** and aniline (Scheme 3).

Aniline **3b**, required for the aza-Pschorr reaction, was obtained by catalytic reduction of the corresponding nitro analogue **4b** (Scheme 2). In the case of reduction of **4c**, slow cyclization of the resulting amine **3c** to the benzimidazole **8** was observed and pure amine **3c** free of **8** could not be isolated. The process was acid-catalyzed, as demonstrated by formation of **8** in a nearly quantitative yield by treatment of the reaction mixture in EtOH with a drop of HCl. The starting 8-bromo derivative **6-Br** was obtained in a way analogous to that of the preparation^12^ of the 8-fluoro analogue **6-F** and will be described elsewhere.

### Cyclization

**Scheme 4:**
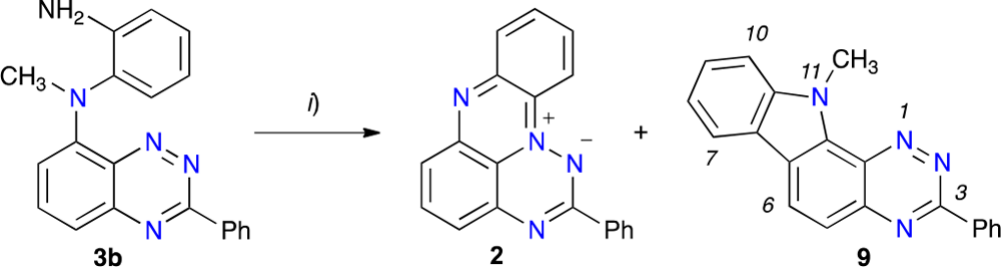
Pschorr-Type Cyclization of Derivative **3b** Reagents and conditions: (*i*) *t*-BuONO (6 equiv), PhCl, N_2_, 2.5 h, 70 °C, **2** 23% yield and **9** 10% yield. A partial numbering system for **9** is shown.

Investigation of the formation of **1N** started with the aza-Pschorr cyclization^16^ of amine **3b**. Thus, treatment of **3b** with *t*-BuONO in PhCl under N_2_ at 70 °C resulted in the formation of two major products: a yellow and a more polar dark green, according to TLC analysis of the crude reaction mixture (Scheme 4). Chromatographic separation and extensive spectroscopic analysis revealed that the less polar yellow solid product is 11-methyl-3-phenyl-11*H*-[1,2,4]triazino[6,5-*a*]carbazole (**9**) isolated in 10% yield, while the green solid was identified as zwitterion **2** obtained in 23% yield. The former represents a new heterocyclic system, while the isolation of **2** suggests that the desired radical **1N-b** was formed as a transient, unstable species under the reaction conditions (*vide infra*). Therefore, the milder photocyclization method^17^ was tested for the preparation of **1N**.

**Scheme 5:**
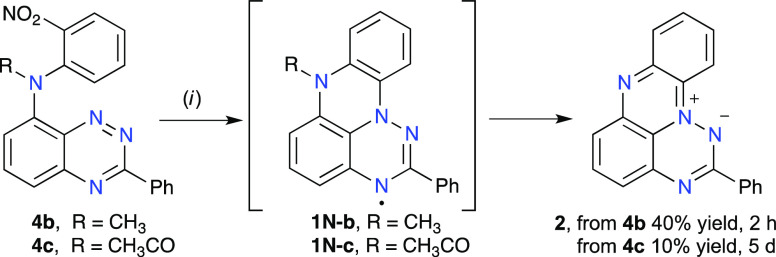
Photocyclization of **4b** and **4c** Reagents and conditions: (*i*) For **4b**: *hv*, CH_2_Cl_2_, 2 h, 40% yield. For **4c**: *hv*, EtOH, 5 d, 10% yield.

Irradiation of dilute (∼1 mM) solutions of the *N*-Me derivative **4b** in CH_2_Cl_2_ with a 300 W halogen lamp resulted in full consumption of the starting material after 2 h, and zwitterion **2** was obtained in 40% yield as the only isolable product (Scheme 5). The *N*-Ac derivative **4c** did not photocyclize in CH_2_Cl_2_ solutions; however, changing the solvent to EtOH led to the formation of zwitterion **2** isolated in 10% yield after *ca*. 5 days of irradiation, along with recovered unreacted triazine **4c**. The *N*-unsubstituted derivative **4a** was stable under these conditions: it did not show any photocyclization products after 24 h and was quantitatively recovered.

For comparison purposes, *N*-Me derivative **5b** lacking the nitro group was also irradiated under the conditions described above for **4b**. No photochemical products were observed after 4 days, and the starting material was recovered in full.

### Molecular and Crystal Structures

**Figure 4:**
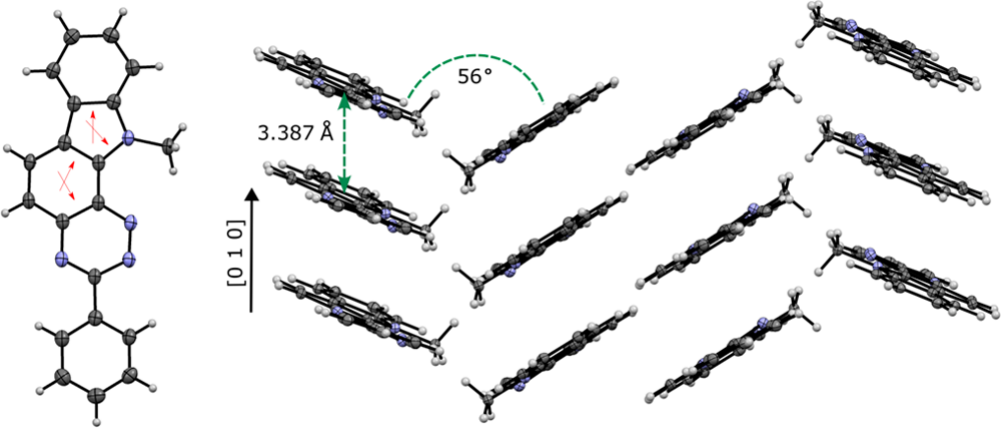
Left: Molecular structure of **9**. Atomic displacement ellipsoids are drawn at the 50% probability level. The red arrows point to bonds most affected by ring fusion of **10** with indole. Right: Partial crystal packing of **9**.

The molecular structure of carbazole derivative **9** was confirmed with the single-crystal X-ray diffraction analysis of a yellow needle-shaped monoclinic crystal belonging to the *P*2_1_/*n* space group. The crystals of **9** were grown from AcOEt by a slow evaporation method. Results are shown in Figure 4, and full data are provided in the Supporting Information.

The asymmetric unit contains one molecule of **9** (Figure 4). The heterocyclic core of the molecule is nearly planar, while the C(3)-phenyl group is twisted relative to the core plane by 5.9°. The dimensions of the benzo[*e*][1,2,4]triazine fragment in **9** are similar to those found in 3-phenylbenzo[*e*][1,2,4]triazine (**10**).^19^ Thus, the most differences in the interatomic distances are within 2σ with the average of 0.005 Å for all values. The largest changes in bond lengths in parent **10** upon fusing with indole are observed for the C(7)–C(8) bond, the common edge with the indole fragment (+0.036 Å), and the C(4a)–C(8a) bond (+0.019 Å). Without these two distances, the average difference between the two structures is less than 1σ (0.002 Å). Similar results are obtained for the indole fragment in which the C(2)–C(3) bond in the parent structure^20^ (the common edge connecting indole and **10**) and N(1)–C(2) are expanded in **9** by +0.052 Å and +0.019 Å, respectively, while the C(3a)–C(7a) is contracted by −0.029 Å.

The N(11)–Me bond length in **9** (1.459(2) Å) is close to that reported for *N*-Me carbazole (1.456(4) Å),^21^ but unlike in the latter, the methyl group in **9** assumes a nearly ideal eclipsed conformation relative to the carbazole system.

Molecules of **9** form slipped stacks with the interplanar distance of 3.387 Å and the slippage angle of 23.8°. The stacks are arranged along the [010] direction in an alternating *syn*- and *anticlinic* pattern with the tilt angle between the stacks of 56° (Figure 4). The crystal packing of **9** is governed by short CH_2_–H···N(2) contacts (2.582 Å, 0.168 inside the van der Waals separation) between molecules in the anticlinic stacks.

### Mechanistic Considerations

**Scheme 6:**
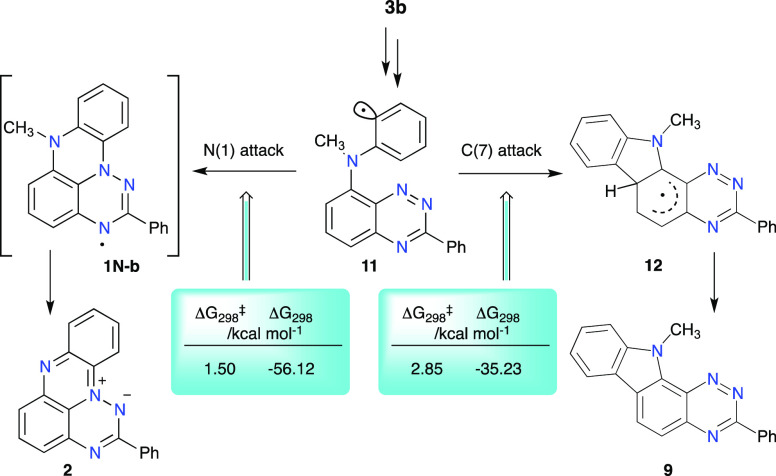
Proposed Mechanism for the Formation of Zwitterion **2** and Carbazole **9** Thermodynamic parameters calculated with the B3LYP/6-311++G(d,p)//B3LYP/6-311G(d,p) method in the PhCl dielectric medium.

Results indicate that radicals **1N-b** and **1N-c** are formed in the Pschorr and photochemical processes, but they are unstable under the reaction conditions and decompose to zwitterion **2**. The possible formation of **1N-b** in the former process is further supported by the isolation of carbazole derivative **9**, a product of Pschorr cyclization of transient phenyl radical **11** at the C(7) position (Scheme 6). Therefore, it can be assumed that radical **11** also cyclizes at the N(1) position leading to **1N-b**, which subsequently fragments to give **2**. DFT analysis of both cyclization pathways indicates that the activation energy required for the N(1) attack (Δ*G*^‡^_298_ = 1.50 kcal mol^–1^) is lower than that for the C(7) attack (Δ*G*^‡^_298_ = 2.85 kcal mol^–1^, Scheme 6). This 1.35 kcal mol^–1^ difference in the activation energies corresponds to an approximate 7:1 ratio (at 70 °C) of the two products, which is consistent with the observed yield of carbazole **9** (10%). The calculated activation energies for the cyclization of **11** are significantly lower than those for the oxygen analogue: Δ*G*^‡^_298_ = 3.29 kcal mol^–1^ for the N(1) attack and Δ*G*^‡^_298_ = 10.46 kcal mol^–1^ for the C(7) attack. Due to a large difference between the two energies (ΔΔ*G*^‡^_298_ = 7.17 kcal mol^–1^), radical **1O** is the only observed product of the aza-Pschorr cyclization.^16^ The higher rate of formation of radical **1N-b** than adduct **12** is associated with a significantly larger exergonic effect (by about 21 kcal mol^–1^) of the former cyclization process.

**Figure 5:**
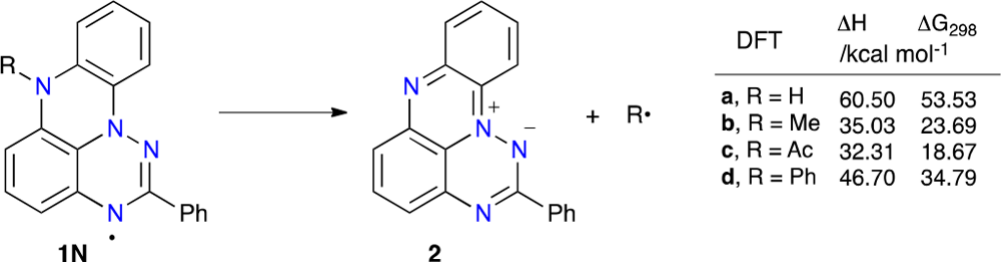
Thermodynamic parameters for fragmentation of radicals **1N** calculated with the B3LYP/6-311++G(d,p)//B3LYP/6-311G(d,p) method in a PhCl dielectric medium.

For a better understanding of the nature of radicals **1N**, their thermodynamic stability against fragmentation and formation of zwitterion **2** was assessed with the DFT method. Results shown in Figure 5 indicate that all fragmentation processes are endergonic, with the highest energy change Δ*G*_298_ = 53.53 kcal mol^–1^ for the parent radical **1N-a**. Since hydrogen transfer processes are generally easy (*e.g*., to O_2_), it can be assumed that this process dominates in the formation of **2** instead of the direct homolysis of the N–H bond.

The free energy change Δ*G*_298_, calculated to be below 24 kcal mol^–1^, indicates that homolysis of the N–Me and N–Ac bonds in radicals **1N** and formation of **2** are feasible at ambient temperature, which is consistent with experimental observations. In contrast, the calculated free energy change in the homolysis of the N–Ph bond in **1N-d** is significantly higher. The calculated Δ*G*_298_ = 34.79 kcal mol^–1^ suggests that this process is ineffective at temperatures below 100 °C and indicates that *N*-Ar derivatives **1N** could be stable to isolation.

Finally, photocyclization of three benzo[*e*][1,2,4]triazinyls, **4b**, **4c**, and **5b**, was briefly investigated with TD-DFT methods. Electronic excitations calculated with the TD-B3LYP/6-31G(d,p)//B3LYP/6-311G(d,p) method indicate that the lowest excited singlet is ^1^(π,π*) for **4b** and **5b**, while for **4c** the S_1_ state is ^1^(n,π*). The *n* HOMO–1 and π LUMO are localized on the benzo[*e*][1,2,4]triazine ring, while the π HOMO involves mainly the arylamino substituent at the C(8) position in all three derivatives. In all cases, the cyclization can occur at the S_1_ state, although in the case of **4c** a facile intersystem crossing (ISC) to the triplet electron manifold is possible.

The energetics of the photocyclization process of three model precursors, **4b**′, **4c**′, and **5b**′, in which the C(3) phenyl group was replaced with an H atom, was investigated at the TD-B3LYP/6-31G(d,p) level of theory in CH_2_Cl_2_ dielectric medium. Analysis of geometries optimized at the S_1_ state revealed a particularly short nonbonding distance of 2.743 Å between the NO_2_ group *ipso* carbon atom and the N(1) nitrogen atom in **4b**′. This is consistent with intramolecular donor–acceptor interactions between the two sites. The *ipso* carbon becomes more electrophilic upon photoinduced shift of electron density from the NMe group toward the NO_2_ group. Such a reorganization of electron density upon photoexcitation is less efficient for the NAc derivative **4c**′ and impossible for the **5b**′ analogue lacking the NO_2_ group. Consequently, the N(1)···C distance is longer in **4c**′ with relaxed S_1_ geometry, although still well inside the van der Waals separation (3.175 Å), while in **5b**′ the distance is 3.742 Å. This demonstrates the particular role of the NO_2_ group in efficient photocyclization processes.

**Figure 6:**
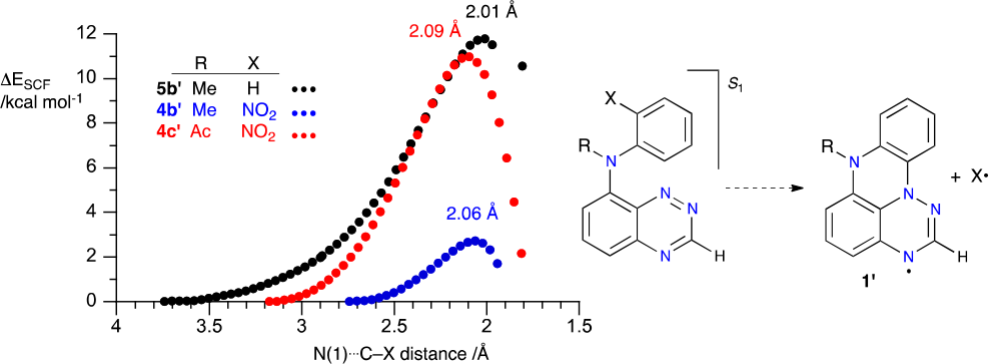
Relative energy of PES relaxed scans along the N(1)···C vector leading to **1N-b**′ and **1N-c**′ in the S_1_ state of models **4b**′, **4c**′, and **5b**′ obtained at the TD-B3LYP/6-31G(d,p) level of theory in the CH_2_Cl_2_ dielectric medium.

Relaxed scans of the potential energy surface (PES) in the S_1_ state for all three model precursors revealed that the barrier to cyclization at the N(1) position is the lowest for the *N*-Me derivative **4b**′ with Δ*E*_SCF_ = 2.72 kcal mol^–1^ (Figure 6). The *N*-Ac analogue **4c**′ has about a 4 times higher barrier (10.97 kcal mol^–1^), and the highest barrier is found for the *N*-Me derivative lacking the NO_2_ group (11.79 kcal mol^–1^). These results are consistent with experimental observations of fast consumption of starting **4b** and very slow or no conversion for **4c** and **5b**, respectively.

### Characterization of Zwitterion **2**

**Figure 7:**
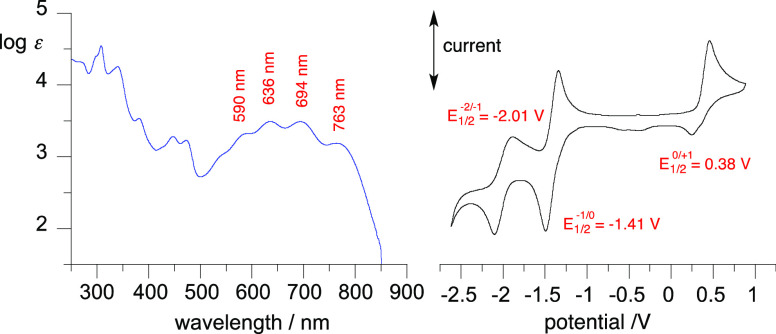
Left: Electronic absorption spectrum of **2** in CH_2_Cl_2_. Right: Cyclic voltammogram for **2** and half-wave potentials referenced to the Fc/Fc^+^ couple (IUPAC convention); 0.5 mM in CH_2_Cl_2_ [*n*-Bu_4_N]^+^ [PF_6_]^−^ (50 mM), at *ca*. 20 °C, 50 mV s^–1^, scans from 0 V in the anodic direction; glassy carbon working electrode, Pt counter electrode, and Ag/AgCl pseudoreference electrode.

The parent zwitterion **2** was characterized by UV–vis spectroscopic and electrochemical methods. Electronic spectroscopy conducted in CH_2_Cl_2_ solutions revealed high energy absorption bands in the region 230–500 nm and a group of five overlapping, moderate intensity bands in the visible range extending to the near IR region (Figure 7). The lowest energy absorption maximum was found at 763 nm (log ε = 3.19), and the optical band gap was determined from the onset of absorption as 1.50 eV. The spectrum of **2** is qualitatively similar to that of the recently reported 4-methyl derivative with a maximum of the lowest energy band at 725 nm in the same solvent.^15^

**Figure 8:**
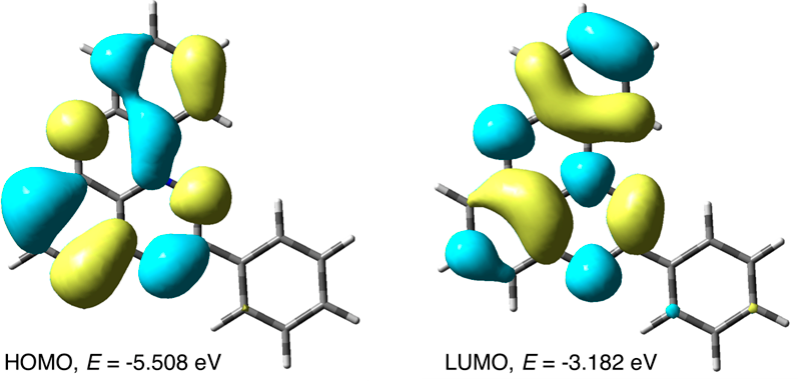
FMO contours and energies of **2** relevant to the low energy excitation obtained with the B3LYP/6-311++G(d,p)//B3LYP/6-311G(d,p) method in a CH_2_Cl_2_ dielectric medium. MO isovalue = 0.020.

According to the TD-DFT results, the lowest excitation is calculated at 673 nm (*f* = 0.076), involving almost exclusively the HOMO → LUMO transition (97%). Both FMOs are approximately evenly distributed over the heterocyclic core, and the difference in their energies is 2.33 eV (Figure 8).

Electrochemical analysis of zwitterion **2** revealed two quasi-reversible reduction processes with half-wave potentials *E*°_1/2_ at −1.41 and −2.01 V and a poorly reversible oxidation process with *E*°_1/2_ = 0.38 V vs the Fc/Fc^+^ couple (Figure 7).

### Comparison of Planar Blatter Radicals

Finally, the effect of the annulating heteroatom on the electronic properties of the planar Blatter radical was assessed by using DFT methods. For comparison purposes, radical **1N-d** with the N(7)–Ph group was used as the most likely to be isolable stable species.

**Figure 9:**
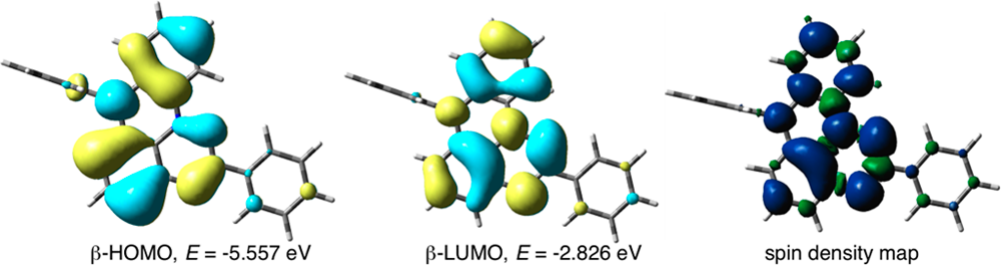
Left: DFT derived contours and energies for β-FMO relevant to low energy excitation in radical **1N-d** (MO isovalue = 0.020). Right: Spin map of **1N-d** with positive density (blue) and negative density (green). Density = 0.0006.

Data listed in Table 1 demonstrate that annulation and consequently planarization of the Blatter radical result in significant bathochromic and hyperchromic shifts of the low energy absorption band from 480 nm (*f* = 0.028) in **Blatter** to 666 nm (*f* = 0.063) in **1N-d**. In **Blatter** and **1O** the S_0_ → S_2_ excitation is over an order of magnitude more probable than the S_0_ → S_1_ process and hence more relevant to experimental observations. The main component of this excitation is the β-HOMO → β-LUMO transition in all four radicals. The density distributions of these two β-FMOs are similar in all three planar Blatter radicals and shown for **1N-d** in Figure 9.

**Table 1:** Comparison of DFT Derived Electronic Properties of the Blatter Radical and Its Planar Analogues^a^

radical	λ_max_^b^^,^^c^/nm	*f*^b^^,^^c^	β-HOMO^c^/eV	β-LUMO^c^/eV	α-HOMO^c^/eV	RDV^–1^ d
**Blatter**	480^e^	0.028^e^	–6.478	–2.937	–4.939	3.931
**1O**	587^e^	0.053^e^	–6.084	–3.065	–4.933	4.206
**1S**	645^f^	0.045^f^	–5.953	–3.169	–5.019	4.321
**1N-d** (R = Ph)	666^f^	0.063^f^	–5.557	–2.826	–4.617	4.746

a Single-point calculations in the CH_2_Cl_2_ dielectric medium for geometry obtained at the UB3LYP/6-311G(d,p) level of theory.

b Lowest energy excitation with significant *f* involving mainly the β-HOMO → β-LUMO transition.

c Obtained with the TD-UB3LYP/6-311++G(d,p) method.

d Obtained with the UB3LYP/EPR-II method in the CH_2_Cl_2_ medium.

e The S_0_ → S_2_ excitation.

f The S_0_ → S_1_ excitation.

The bathochromic shift calculated in the series is related to the progressively increasing energy of β-HOMO from −6.48 eV in prototypical **Blatter** to −5.56 eV in **1N-d**. At the same time, the β-LUMO is destabilized in the latter by over 0.11 eV relative to that in **Blatter**. In contrast, the chalcogens exert weaker effects on the β-HOMO and stabilize the β-LUMO up to 0.23 eV for **1S**. Analysis of the α electron manifold indicates that the annulating nitrogen atom significantly destabilizes the SOMO (α-HOMO) by 0.32 eV, while the chalcogens have a marginal impact.

**Figure 10:**
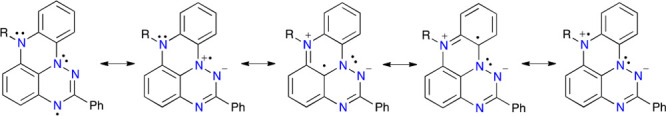
Selected resonance forms for **1N**.

The annulating heteroatom also affects the spin density distribution in the radicals. Calculations of the inverse of the Radical Delocalization Value^22^ (RDV^–1^) demonstrate that planarization of the Blatter radical indeed enhances spin delocalization and the N–Ph annulating group is the most effective in changing the RDV^–1^ value from 3.931 in **Blatter** to 4.746 in **1N-d** (Table 1). It needs to be stressed that the N–Ph group is orthogonal to the heterocycle plane, and hence, the two π systems do not interact (Figure 9). Consequently only 1% of spin is transferred to N(7)–Ph, relative to 5% transferred to the coplanar C(2)–Ph in **1N-d**, through a spin polarization mechanism. The high effectiveness of the N–R group in spin delocalization is related to the more efficient injection of electron density into the benzo[*e*][1,2,4]triazine fragment and stabilization of the zwitterionic resonance forms, as shown in Figure 10. This is consistent with high spin concentration on the *peri*-annulating heterocycle: it is 6.9% for N–Ph in **1N-d**, while it is about 3.3% for O and S in **1O** and **1S**, respectively. In agreement with the proposed mechanism, spin density in the *N*-Me derivative **1N-b** is higher, 7.2%, while that in the *N*-Ac **1N-c** is significantly lower (1%).

## Conclusions

The N-*peri*-annulated planar Blatter radicals **1N** still remain elusive. Two investigated approaches to this class of radicals using several precursors yielded the zwitterion **2** as the main product, which suggests that the desired radicals were formed as the transient species before undergoing fragmentation. This observation augmented with DFT calculations indicates the low thermal stability of *N*-alkyl and *N*-acyl derivatives **1N** against homolytic bond cleavage. Further support for this conclusion is provided by the isolation of small quantities of carbazole derivative **9**. Product **9** is apparently formed by the Pschorr cyclization process of the transient phenyl radical intermediate, which can cyclize either at the C(7) position of the benzo[*e*][1,2,4]triazine giving **9** or at the N(1) position. The latter leads to radical **1N** that undergoes fragmentation with the loss of the N(7) substituent and the formation of the isolated zwitterion **2**.

In accordance with the proposed mechanism, DFT calculations indicate that the loss of the Me and Ac substituent from the N(7) position is endergonic with Δ*G*_298_ less than 24 kcal mol^–1^ and hence feasible at ambient temperature. A much higher Δ*G*_298_ is calculated for the *N*-Ph derivative (**1N-d**), which suggests the stability of *N*-aryl radicals **1N** under ambient conditions. The Δ*G*_298_ calculated for *N*-H radical **1N-a** is the highest among the four radicals. The lack of observation of **1N-a** is presumably related to the oxidative instability of this species under Pschorr reaction conditions and the presence of atmospheric oxygen.

Analysis of the electronic structure of the three planar radicals indicates that the *peri*-annulating nitrogen atom has the strongest effect among the three heteroatoms on the energy of β-FMOs, and hence on the position of the low energy absorption bands and also on spin delocalization. Despite the orthogonal orientation of the *N*-aryl group, it can affect the radical’s π system through the inductive effect, hence controlling the electronic properties of the system. For these reasons, *N*-aryl radicals **1N** constitute attractive synthetic targets for materials with tunable properties.

## Computational Details

Quantum-mechanical calculations were carried out using the Gaussian 09 suite of programs.^23^ Geometry optimizations were undertaken at the (U)B3LYP/6-311G(d,p) level of theory in a vacuum. Vibrational frequencies were used to characterize the nature of the stationary points and to obtain thermodynamic parameters. Transition states for the formation of **1N-b** and **9** in the Pschorr reaction were located using the UB3LYP/6-311G(d,p) method and the QST3 algorithm. The three input geometries were obtained from relaxed PES scans. The final SCF energies for mechanistic considerations were obtained at the (U)B3LYP/6-311++G(d,p)//(U)B3LYP/6-311G(d,p) level of theory in the PhCl dielectric medium requested with the SCRF(Solvent = C6H5Cl) keyword (PCM model).^24^

Electronic excitation energies in the CH_2_Cl_2_ dielectric medium were obtained for derivatives **1** and **2** at the (U)B3LYP/6-311++G(d,p)//(U)B3LYP/6-311G(d,p) level of theory using the time-dependent^25^ DFT method supplied in the Gaussian 09 package. Solvation models in calculations were implemented by the PCM model^24^ using the SCRF(Solvent=CH2Cl2) keyword.

Atomic spin densities for radicals **1** were calculated using the UB3LYP/EPR-II//UB3LYP/6-311G(d,p) method in the CH_2_Cl_2_ dielectric medium requested with the SCRF(Solvent=CH2Cl2) keyword (PCM model).^24^ For the S atom, 6-31+G(2d) basis sets were requested with the “gen” keyword.

A relaxed scan of the potential energy surface (PES) in the S_1_ state for model compounds was conducted at the TD-B3LYP/6-31G(d,p) level of theory in the CH_2_Cl_2_ dielectric medium using the “TD=(singlets, root=1, NStates=14)” keyword and 0.04 Å step from the S_1_ state equilibrium geometry.

## Experimental Section

### General Methods

Reagents and solvents were commercially available. Reactions were protected from moisture with a N_2_ atmosphere. Anhydrous Na_2_SO_4_ was used for drying organic extracts, and all volatiles were removed under reduced pressure. Heat in reactions involving elevated temperatures was supplied using oil baths, and reported temperature refers to that of the bath. All reaction mixtures and column eluents were monitored by thin layer chromatography (TLC) using commercial silica gel TLC plates. The plates were observed under UV light at 254 and 365 nm. NMR spectra were obtained at 400 MHz (^1^H) and 100 MHz (^13^C) MHz in CDCl_3_. Chemical shifts were referenced to the solvent (^1^H and ^13^C: 7.26 and 77.16 ppm for CDCl_3_)^26^ using a Bruker Avance 400 spectrometer. Melting points were determined on a Melt-Temp II apparatus in capillaries, and they are uncorrected. High-resolution mass spectrometry (HRMS) measurements were performed by using a Varian 500 MS LS Ion Trap spectrometer. In all cases, little or no fragmentation was observed, and the M+ and MH+ peaks were the most intense signals. IR spectra were measured in KBr pellets with a Bruker Alpha ATR spectrometer. UV–vis spectra were recorded on a Jasco V770 spectrophotometer in spectroscopic-grade CH_2_Cl_2_ at concentrations of (1–10) × 10^–5^ M. Extinction coefficients were obtained by fitting the maximum absorbance at 308 nm against the concentration in agreement with Beer’s law. Irradiations were conducted with a 300 W halogen lamp (“portable halogen work lamp” without the protecting front glass window) equipped with a T3 double-ended RSC base J118 light bulb.

#### Attempted Preparation of Radical **1N-b**. Pschorr Cyclization of *N*^1^-Methyl-*N*^1^-(3-phenylbenzo[*e*][1,2,4]triazin-8-yl)benzene-1,2-diamine (**3b**)

To a solution of amine **3b** (72.0 mg, 0.22 mmol) in dry PhCl (1 mL), under N_2_ atm, *t*-BuONO (158 μL, 1.32 mmol, 6 equiv) was added dropwise with stirring. The resulting mixture was gradually heated to 70 °C over a period of 15 min and kept at 70 °C for 2.5 h, after which time TLC analysis showed complete consumption of substrate **3b**. The reaction mixture was cooled, volatiles were evaporated, and the dark residue was chromatographed on passivated silica gel using AcOEt/pet. ether mixture (1:4) as eluent. The first yellow fraction contained 7.0 mg (10% yield) of carbazole **9**, which was recrystallized from AcOEt/hexane. The second, more polar green fraction contained 15.0 mg (23% yield) of zwitterion **2**, which was also recrystallized from AcOEt. For analytical data, see below.

#### Attempted Preparation of Radicals **1N-b** and **1N-c** by Photocyclization

A 1 mM solution (100 mL, 0.1 mmol) of *N*-methyl derivative **4b** or **5b** in CH_2_Cl_2_ or *N*-acetyl **4c** in EtOH was placed in a round-bottom flask fitted with a magnetic stirrer and a reflux condenser. The solution was stirred and irradiated with a 300 W halogen lamp, which was placed approximately 30 cm from the flask. The irradiation warmed up the reaction mixture to 30–35 °C. Progress of the reaction was monitored by TLC (AcOEt/hexane, 1:4), and the irradiation was stopped either after all of the starting material was consumed (2 h for **4b**) or after an extended period of time (up to 5 days for **4c**). The solvent was evaporated, the residue was adsorbed on a passivated silica gel (1% Et_3_N), and the products were separated by column chromatography, using passivated silica gel (1% Et_3_N) and with a AcOEt/hexane mixture (1:4) as the eluent. The isolated product was recrystallized from AcOEt. Both precursors gave the same zwitterion **2** with yields of 40% for precursor **4b** (in CH_2_Cl_2_) and 10% for **4c** (in EtOH). In the latter case, the unreacted starting **4c** was recovered in 60% yield, while, in the case of irradiation of **5b**, the entire starting material was recovered.

#### 2-Phenyl[1,2,4]triazino[5,6,1-*de*]phenazin-12-ium-3-ide (**2**)

Yield 15.0 mg (23%) from **3b**. Mp 212–215 °C (AcOEt). ^1^H NMR (400 MHz, CDCl_3_) δ: 8.47 (d, *J* = 9.0 Hz, 1H), 8.28 (dd, *J*_1_ = 8.1 Hz, *J*_2_ = 1.7 Hz, 2H), 7.72 (d, *J* = 8.8 Hz, 1H), 7.67–7.60 (m, 2H), 7.53–7.48 (m, 3H), 7.43 (ddd, *J*_1_ = 8.5 Hz, *J*_2_ = 6.7 Hz, *J*_3_ = 1.4 Hz, 1H), 7.31 (dd, *J*_1_ = 8.6 Hz, *J*_2_ = 1.1 Hz, 1H), 6.97 (dd, *J*_1_ = 7.8 Hz, *J*_2_ = 1.1 Hz, 1H). ^13^C{^1^H} NMR (101 MHz, CDCl_3_) δ: 163.3, 147.9, 146.8, 145.9, 135.3, 134.5, 132.4, 131.4, 129.6, 128.5, 128.2, 127.6, 125.6, 125.0, 118.3, 117.5, 111.9. IR (KBr): ν 1595m, 1485m, 1440m, 1349s, 1255m, 766s, 693s cm^–1^. UV (CH_2_Cl_2_) λ_max_ (log ε) 763 (3.19), 694 (3.49), 636 (3.49), 590 (3.32), 473 (3.23), 448 (3.28), 382 (3.53), 340 (4.25), 308 (4.55), 266 (4.32), 248 (4.36) nm. HRMS (ESI+ – TOF) *m*/*z* [M + H]^+^ calcd for C_19_H_13_N_4_: 297.1140; found: 297.1138; Anal. Calcd for C_19_H_12_N_4_: C, 77.01; H, 4.08; N, 18.91. Found: C, 76.95; H, 4.05; N, 18.83%.

#### *N*^1^-Methyl-*N*^1^-(3-phenylbenzo[*e*][1,2,4]triazin-8-yl)benzene-1,2-diamine (**3b**)

To a solution of nitro derivative **4b** (140 mg, 0.40 mmol) in EtOH/THF (20 mL, 3:1) at *ca*. 20 °C, Pd/C (10 mol %, 46 mg, 0.4 mmol) was added and the mixture was stirred under a H_2_ atmosphere (55 psi) overnight. The mixture was filtered through a short pad of Celite and rinsed with EtOH (3 × 10 mL), and volatiles were removed *in vacuo*. The resulting crude amine **3b** was purified by column chromatography (CH_2_Cl_2_/pet. ether, 7:3) followed by recrystallization from EtOH to give 120 mg (94% yield) of the pure *title amine***3b** as red crystals: mp 214–217 °C (EtOH). ^1^H NMR (400 MHz, CDCl_3_) δ: 8.67–8.64 (m, 2H), 7.75 (t, *J* = 8.2 Hz, 1H), 7.57–7.53 (m, 3H), 7.49 (dd, *J*_1_ = 8.4 Hz, *J*_2_ = 0.8 Hz, 1H), 7.07 (td, *J*_1_ = 7.6 Hz, *J*_2_ = 1.5 Hz, 1H), 6.98 (dd, *J*_1_ = 7.8 Hz, *J*_2_ = 1.0 Hz, 1H), 6.90 (ddd, *J*_1_ = 9.1 Hz, *J*_2_ = 8.0 Hz, *J*_3_ = 1.4 Hz, 2H), 6.69 (td, *J*_1_ = 7.6 Hz, *J*_2_ = 1.6 Hz, 1H), 4.16 (brs, 2H), 3.63 (s, 3H). ^13^C{^1^H} NMR (101 MHz, CDCl_3_) δ: 158.9, 148.6, 143.2, 142.5, 141.5, 137.6, 136.4, 135.8, 131.3, 128.9, 128.8, 127.3, 126.2, 119.20, 119.18, 116.6, 114.8, 42.7. IR (KBr): ν 3446, 3385, 1590, 1566, 1526, 1490, 1377, 793, 744, 710, 693 cm^–1^. HRMS (ESI+ – TOF) *m*/*z* [M + H]^+^ calcd for C_20_H_18_N_5_: 328.1562; found: 328.1557. Anal. Calcd for C_20_H_17_N_5_: C, 73.37; H, 5.23; N, 21.39. Found: C, 73.12; H, 5.52; N, 21.38%.

#### *N*-(2-Nitrophenyl)-3-phenylbenzo[*e*][1,2,4]triazin-8-amine (**4a**)

A round-bottom flask was charged with 2-nitroaniline (106.4 mg, 0.77 mmol), 8-bromo-3-phenylbenzo[*e*][1,2,4]triazine (**6-Br**, 200 mg, 0.70 mmol), Pd_2_(dba)_3_ (32 mg, 5 mol %), 2-dicyclohexylphosphino-2′-(*N*,*N*-dimethylamino)biphenyl (DavePhos, 21 mg, 7.5 mol %), Cs_2_CO_3_ (456 g, 1.4 mmol), and dry toluene (2 mL). The mixture was purged with N_2_ for 10 min at *ca*. 20 °C and then stirred at 120 °C for 24 h under a N_2_ atmosphere. Upon completion, the reaction mixture was cooled, filtered through a pad of Celite, and washed with CH_2_Cl_2_ (20 mL) and AcOEt (20 mL), and volatiles were removed *in vacuo*. The residue was purified by column chromatography using AcOEt/pet. ether (7:3) as eluent to afford 196 mg (82% yield) of the *title compound***4a** as red crystals: mp 248–251 °C (AcOEt). ^1^H NMR (500 MHz, CDCl_3_) δ: 11.15 (s, 1H), 8.78–8.76 (m, 2H), 8.30 (dd, *J*_1_ = 8.5 Hz, *J*_2_ = 1.6 Hz, 1H), 7.93 (dd, *J*_1_ = 8.5 Hz, *J*_2_ = 1.3 Hz, 1H), 7.89 (t, *J* = 8.2 Hz, 1H), 7.72 (d, *J* = 7.8 Hz, 1H), 7.66 (dd, *J*_1_ = 8.5 Hz, *J*_2_ = 1.0 Hz, 1H), 7.63–7.59 (m, 4H), 7.10 (ddd, *J*_1_ = 7.8 Hz, *J*_2_ = 7.2 Hz, *J*_3_ = 1.3 Hz, 1H). ^13^C{^1^H} NMR (101 MHz, CDCl_3_) δ: 160.9, 142.6, 139.4, 138.8, 138.2, 137.9, 136.8, 135.6, 135.3, 131.8, 129.1, 129.0, 127.2, 121.3, 120.9, 119.0, 112.4. IR (KBr): ν 3335, 1619, 1574, 1501, 1384, 1327, 1262, 1148, 731, 684 cm^–1^. HRMS (AP+ – TOF) *m*/*z* [M + H]^+^ calcd for C_19_H_14_N_5_O_2_: 344.1147; found: 344.1140. Anal. Calcd for C_19_H_13_N_5_O_2_: C, 66.47; H, 3.82; N, 20.40. Found: C, 66.32; H, 3.90; N, 20.36%.

#### *N*-Methyl-*N*-(2-nitrophenyl)-3-phenylbenzo[*e*][1,2,4]triazin-8-amine (**4b**)

To a solution of amine **4a** (90.0 mg, 0.26 mmol) in dry DMA (1 mL), under a N_2_ atmosphere, was added NaH (19.0 mg, 0.78 mmol) and the reaction mixture was stirred at *ca*. 20 °C for 10 min. Methyl iodide (0.050 mL, 0.78 mmol) was then added in one portion, and the reaction was stirred under N_2_, at *ca*. 20 °C for 2 h. The organic layer was washed with H_2_O (10 mL), brine (5 mL), and dried (Na_2_SO_4_), and the solvent was removed *in vacuo* to afford 83.0 mg (89% yield) of the *title compound***4b** as dark red crystals. The product was used without further purification. Mp 149–153 °C (CH_2_Cl_2_/pet. ether). ^1^H NMR (500 MHz, CDCl_3_) δ: 8.66–8.62 (m, 2H), 7.88 (dd, *J*_1_ = 8.2 Hz, *J*_2_ = 1.6 Hz, 1H), 7.81 (t, *J* = 8.2 Hz, 1H), 7.61–7.57 (m, 2H), 7.55–7.52 (m, 3H), 7.41 (dd, *J*_1_ = 8.1 Hz, *J*_2_ = 1.3 Hz, 1H), 7.28 (ddd, *J*_1_ = 7.8 Hz, *J*_2_ = 7.1 Hz, *J*_3_ = 1.3 Hz, 1H), 7.15 (dd, *J*_1_ = 8.0 Hz, *J*_2_ = 1.1 Hz, 1H), 3.71 (s, 3H). ^13^C{^1^H} NMR (126 MHz, CDCl_3_) δ: 159.1, 146.3, 145.1, 144.9, 142.5, 140.8, 136.2, 135.7, 134.3, 131.4, 128.94, 128.90, 127.7, 126.1, 125.1, 121.5, 116.6, 43.7. HRMS (ESI+ – TOF) *m*/*z* [M + H]^+^ calcd for C_20_H_16_N_5_O_2_: 358.1304; found: 358.1302. Anal. Calcd for C_20_H_15_N_5_O_2_: C, 67.22; H, 4.23; N, 19.60. Found: C, 67.26; H, 4.29; N, 19.57%.

#### *N*-(2-Nitrophenyl)-*N*-(3-phenylbenzo[*e*][1,2,4]triazin-8-yl)acetamide (**4c**)

To a solution of amine **4a** (190 mg, 0.55 mmol) in acetic anhydride (0.260 mL, 2.75 mmol), ZnCl_2_ (75 mg, 0.55 mmol) was added in one portion, and the reaction mixture was stirred at 80 °C for 28 h. Upon completion, the reaction mixture was cooled, water (5 mL) was added, and the mixture was stirred for 10 min. The organic product was extracted with AcOEt (3 × 10 mL), the collected organic layers were dried (Na_2_SO_4_) and filtered, and volatiles were removed under reduced pressure. The resulting mixture was chromatographed with AcOEt/pet. ether (3:2) as eluent to give 163 mg (76% yield) of the *title acetamide***4c** as yellow crystals: mp 179–182 °C (toluene/CH_2_Cl_2_). ^1^H NMR (400 MHz, CDCl_3_) δ: 8.80–8.77 (m, 2H), 8.15 (d, *J* = 8.0 Hz, 1H), 8.01 (dd, *J*_1_ = 8.0 Hz, *J*_2_ = 1.4 Hz, 2H), 7.64–7.62 (m, 3H), 7.55–40 (m, 3H), 2.03 (brs, 3H); ^13^C{^1^H} NMR (101 MHz, CDCl_3_) δ: 171.1, 160.5, 147.0, 143.0, 142.4, 140.4, 136.2, 135.1, 134.2, 132.2, 130.3, 130.2, 129.3, 129.1, 128.2, 125.3, 23.0. IR (neat): ν 1678, 1526, 1505, 1495, 1306, 1291, 1273, 713, 701, 693 cm^–1^. HRMS (ESI+ – TOF) *m*/*z* [M + H^+^] Calcd for C_21_H_16_N_5_O_3_: 386.1253; found: 386.1258. Anal. Calcd for C_21_H_15_N_5_O_3_: C, 65.45; H, 3.92; N, 18.17. Found: C, 65.38; H, 3.97; N, 18.11%.

#### *N*,3-Diphenylbenzo[*e*][1,2,4]triazin-8-amine (**5a**)

The amine was obtained as described for **4a** using aniline (82.0 mg, 0.88 mmol), bromide **6-Br** (230 mg, 0.80 mmol), Pd_2_(dba)_3_ (37 mg, 5 mol %), 2-dicyclohexylphosphino-2′-(*N*,*N*-dimethylamino)-biphenyl (23.8 g, 7.5 mol %), Cs_2_CO_3_ (525 mg, 1.61 mmol), and dry toluene (4 mL). The crude product was purified by column chromatography (ether/pet. ether, 3:2) to afford 208 mg (87% yield) of the *title amine***5a** as red crystals: mp 121–123 °C (ether/pet. ether). ^1^H NMR (400 MHz, CDCl_3_) δ: 8.74–8.72 (m, 2H), 8.67 (br s, 1H), 7.78 (t, *J* = 8.5 Hz, 1H), 7.61–7.58 (m, 3H), 7.46–7.42 (m, 4H), 7.38 (td, *J*_1_ = 9.0 Hz, *J*_2_ = 1.0 Hz, 2H), 7.19–7.16 (m, 1H). ^13^C{^1^H} NMR (101 MHz, CDCl_3_) δ: 160.7, 142.7, 142.1, 140.1, 138.2, 138.0, 135.8, 131.5, 129.8, 129.1, 128.8, 124.2, 121.6, 116.0, 107.1. IR (KBr): ν 3339, 1682, 1533, 1357, 1006, 802, 703 cm^–1^. HRMS (ESI+ – TOF) *m*/*z* [M + H]^+^ calcd for C_19_H_15_N_4_: 299.1297; found: 299.1288. Anal. Calcd for C_19_H_14_N_4_: C, 76.49; H, 4.73; N, 18.78. Found: C, 76.32; H, 4.96; N, 18.73%.

#### *N*-Methyl-*N*,3-diphenylbenzo[*e*][1,2,4]triazin-8-amine (**5b**)

To a solution of *N*,3-diphenylbenzo[*e*][1,2,4]triazin-8-amine (**5a**, 80.0 mg, 0.27 mmol) in DMA (2 mL), under an inert atmosphere, was added NaH (13 mg, 0.54 mmol), and the reaction mixture was stirred at *ca*. 20 °C for 10 min. Methyl iodide (0.051 mL, 0.81 mmol) was added in one portion, and the reaction mixture was stirred under N_2_, at *ca*. 20 °C for 2 h. AcOEt (10 mL) and H_2_O (10 mL) were added; the organic layer was separated, washed with water (×3) and brine (5 mL), and dried (Na_2_SO_4_). Volatiles were removed *in vacuo* to afford 75 mg (90% yield) of the *title compound***5b**, which was recrystallized (CH_2_Cl_2_/pet. ether) giving dark red crystals: mp 134–135 °C (CH_2_Cl_2_/pet. ether). ^1^H NMR (400 MHz, CDCl_3_) δ: 8.74–8.71 (m, 2H), 7.85 (t, *J*_1_ = 8.2 Hz, 1H), 7.75 (dd, *J*_1_ = 8.4 Hz, *J*_2_ = 1.2 Hz, 1H), 7.60–7.55 (m, 3H), 7.43 (dd, *J*_1_ = 7.6 Hz, *J*_2_ = 1.2 Hz, 1H), 7.29–7.25 (m, 2H), 7.05–7.02 (m, 2H), 6.96 (tt, *J*_1_ = 7.3 Hz, *J*_2_ = 1.2 Hz, 1H), 3.72 (s, 3H). ^13^C NMR (101 MHz, CDCl_3_) δ: 159.3, 150.3, 147.9, 142.9, 142.8, 136.1, 135.7, 131.5, 129.4, 129.0, 128.9, 123.3, 123.2, 121.5, 119.2, 42.7. HRMS (ESI+ – TOF) *m*/*z* [M + H]^+^ calcd for C_20_H_17_N_4_: 313.1453; found: 313.1450. Anal. Calcd for C_20_H_16_N_4_: C, 76.90; H, 5.16; N, 17.94. Found: C, 76.65; H, 5.21; N, 18.06.

#### 3-Phenylbenzo[*e*][1,2,4]triazin-8-amine (**7**)

A solution of 8-fluoro-3-phenylbenzo[*e*][1,2,4]triazine^12^ (**6-F**, 22.5 mg, 0.10 mmol) in MeCN (1 mL) was placed in a pressure tube fitted with a magnetic stirrer. Conc. aqueous solution of ammonia (25%, 1.4 mL) was added to the mixture. The pressure tube was closed and heated to 120 °C overnight. The solution changed color from yellow to red. Water was added, the product was extracted with AcOEt (×3), the collected organic layers were dried (Na_2_SO_4_), and volatiles were removed *in vacuo*. The resulting crude product was recrystallized (EtOH) giving 20.0 mg (90% yield) of *title amine***7** as red crystals: mp 190–194 °C (EtOH). ^1^H NMR (400 MHz, CDCl_3_) δ: 8.73–8.70 (m, 2H), 7.75 (dd, *J*_1_ = 8.1 Hz, *J*_2_ = 7.8 Hz, 1H), 7.62–7.55 (m, 3H), 7.33 (dd, *J*_1_ = 8.4 Hz, *J*_2_ = 1.0 Hz, 1H), 6.89 (dd, *J*_1_ = 7.8 Hz, *J*_2_ = 1.0 Hz, 1H), 5.35 (brs, 2H). ^13^C{^1^H} NMR (101 MHz, CDCl_3_) δ: 160.2, 145.5, 142.2, 138.0, 137.8, 135.9, 131.4, 129.0, 128.8, 115.6, 109.6; IR (KBr): ν 3437, 3309, 1633, 1499, 1362, 1335, 781, 702, 682 cm^–1^. HRMS (ESI+ – TOF) *m*/*z* [M + H]^+^ calcd for C_13_H_11_N_4_: 223.0984; found: 223.0986. Anal. Calcd for C_13_H_10_N_4_: C, 70.26; H, 4.54; N, 25.21. Found: C, 70.25; H, 4.58; N, 25.27%.

#### 2-Methyl-1-(3-phenylbenzo[*e*][1,2,4]triazin-8-yl)-1*H*-benzimidazole (**8**)

To a solution of acetamide **4c** (200 mg, 0.52 mmol) in EtOH/CH_2_Cl_2_ (20 mL, 3:1) at *ca*. 20 °C, Pd/C (10 mol %, 33.0 mg, 0.031 mmol) was added and the reaction mixture was stirred under a H_2_ atmosphere (55 psi) overnight. The mixture was filtered through a pad of Celite and rinsed with CH_2_Cl_2_ (3 × 10 mL), and volatiles were removed *in vacuo*. The residue was dissolved in EtOH, 1–2 drops of conc. HCl was added, and the solution was stirred at ambient temperature for 1 h, upon which time TLC analysis showed complete conversion of amine **3c** to benzimidazole **8**. The solvent was evaporated and the crude product was purified using a short silica gel column (pet. ether/AcOEt, 6:4) to give 166 mg (95% yield) of the *title benzimidazole***8** as a yellow solid, which was recrystallized from EtOH: mp >250 °C (EtOH). ^1^H NMR (400 MHz, CDCl_3_) δ: 8.78–8.75 (m, 2H), 8.32 (dd, *J*_1_ = 8.7, *J*_2_ = 1.4 Hz, 1H), 8.17 (dd, *J*_1_ = 8.8, *J*_2_ = 7.4 Hz, 1H), 7.89 (dd, *J*_1_ = 7.4 Hz, *J*_2_ = 1.3 Hz, 1H), 7.83 (d, *J* = 8.0 Hz, 1H), 7.62–7.57 (m, 3H), 7.30 (td, *J*_1_ = 7.7 Hz, *J*_2_ = 1.2 Hz, 1H), 7.18 (td, *J*_1_ = 7.7 Hz, *J*_2_ = 1.2 Hz, 1H), 6.98 (d, *J* = 8.0 Hz, 1H), 2.49 (s, 3H). ^13^C NMR (101 MHz, CDCl_3_) δ: 160.7, 152.8, 142.4, 137.4, 135.4, 134.9, 134.3, 132.3, 131.2, 129.4, 129.3, 129.2, 123.3, 123.1, 119.3, 109.8, 14.9. IR (KBr): ν 1606, 1566, 1504, 1394, 1322, 1277, 1007, 803, 751, 709 cm^–1^; HRMS (ESI+ – TOF) *m*/*z* [M + H]^+^ Calcd for C_21_H_16_N_5_: 338.1406; found: 338.1402; Anal. Calcd for C_21_H_15_N_5_: C, 74.76; H, 4.48; N, 20.76. Found: C, 74.76; H, 4.47; N, 20.52%.

#### 11-Methyl-3-phenyl-11*H*-[1,2,4]triazino[6,5-*a*]carbazole (**9**)

Yield 7.0 mg (10%) from **3b**. Mp: 242–244 °C (AcOEt/hexane); ^1^H NMR (400 MHz, CDCl_3_) δ: 8.80–8.78 (m, 2H), 8.64 (d, *J* = 8.7 Hz, 1H), 8.18 (dd, *J*_1_ = 7.9 Hz, *J*_2_ = 1.0 Hz, 1H), 7.78 (d, *J* = 8.7 Hz, 1H), 7.67 (d, *J* = 8.3 Hz, 1H), 7.64–7.58 (m, 4H), 7.42 (ddd, *J*_1_ = 7.4 Hz, *J*_2_ = 6.9 Hz, *J*_3_ = 1.1 Hz, 1H), 4.77 (s, 3H). ^13^C{^1^H} NMR (101 MHz, CDCl_3_) δ: 158.7, 141.8, 141.5, 139.2, 136.2, 132.8, 131.3, 130.2, 129.1, 128.6, 126.5, 122.6, 121.3, 121.1, 120.2, 118.7, 110.2, 34.1. IR (KBr): ν 1561, 1472, 1371, 1335, 1321, 1241, 805, 737, 715, 687, 642 cm^–1^. HRMS (ESI+ – TOF) *m*/*z* [M + H]^+^ Calcd for C_20_H_15_N_4_: 311.1297; found: 311.1285. Anal. Calcd for C_20_H_14_N_4_: C, 77.40; H, 4.55; N, 18.05. Found: C, 77.48; H, 4.53; N, 18.09%.

## Data Availability

The data underlying this study are available in the published article and its Supporting Information.

## Supporting Information Available

NMR and UV spectra and additional details of XRD, electrochemical, and computational analyses (PDF)
FAIR data, including the primary NMR FID files, for compounds **2**–**5** and **7**–**9** (ZIP)
