# Supplementary material for: Toward N-peri-Annulated Planar Blatter Radical through aza-Pschorr and Photocyclization
Source: J Org Chem. 2023 Nov 24;88(24):17197–205. doi: 10.1021/acs.joc.3c02051 (PMC10729016; doi:10.1021/acs.joc.3c02051)
Supplement: Supplementary file 1 — jo3c02051_si_001.pdf [file jo3c02051_si_001.pdf]

Supporting Information

for

Towards N-*peri*-annulated planar Blatter radical through aza-Pschorr and photocyclization

Patrycja Szamweber,[§] Anna Pietrzak,[#] Georgia A. Zissimou,[§] and Piotr Kaszyński*[§,†,¶]

[§] Centre of Molecular and Macromolecular Studies, Polish Academy of Sciences, 90-363 Łódź, Poland
[#] Faculty of Chemistry, Łódź University of Technology, 90-924 Łódź, Poland
[†] Faculty of Chemistry, University of Łódź, 91-403 Łódź, Poland
[¶] Department of Chemistry, Middle Tennessee State University, Murfreesboro, TN, 37132, USA

corresponding author e-mail address: piotr.kaszynski@cbmm.lodz.pl

Table of contents **Page**

1. NMR spectra …S2
2. XRD data collection and refinement …S12
3. UV-vis spectroscopy …S14
4. Electrochemical results …S14
5. Computational details …S15
   a) geometry optimization and energies ...S15
   b) spin delocalization ...S15
   c) electronic excitation data ...S15
   d) partial output data for TD-DFT calculations ...S16
   e) selected MO contours ...S20
   f) MO energies ...S21
   g) modeling cyclization reactions in the excited state ...S22
6. Archive for DFT calculations …S23
7. References …S35

1. NMR spectra

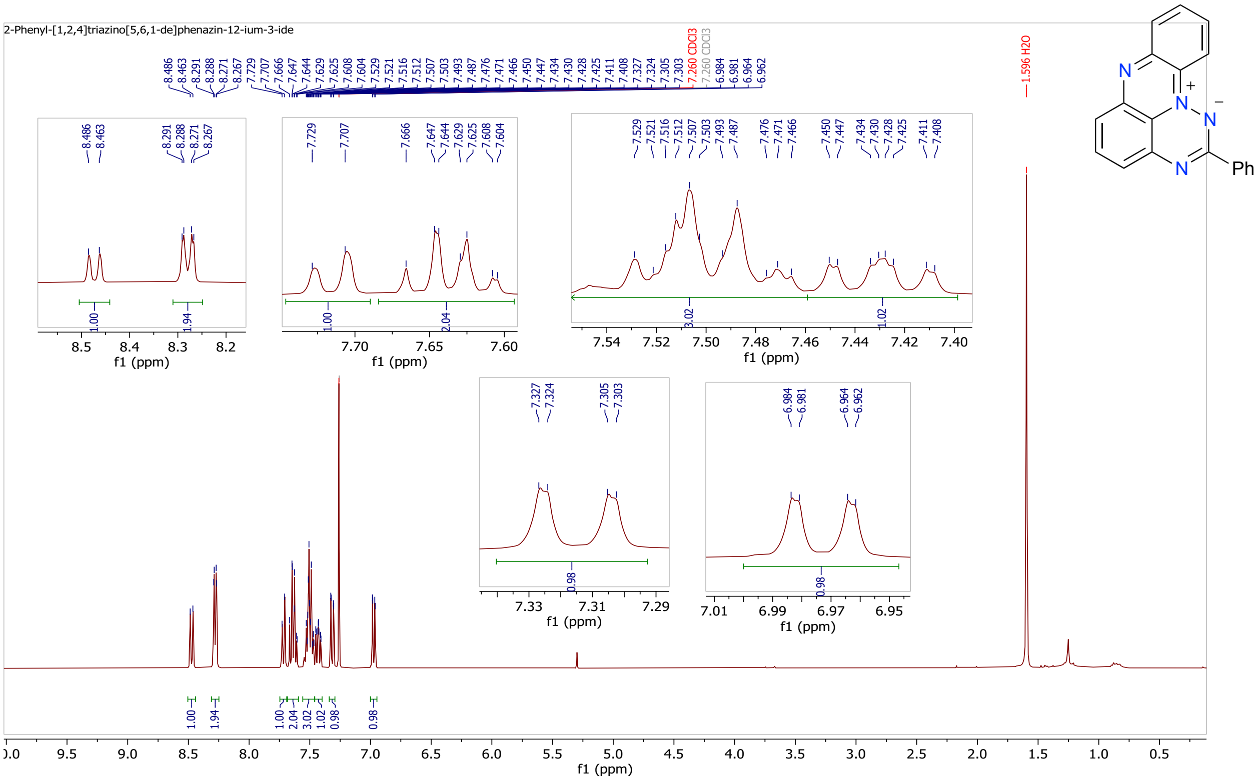

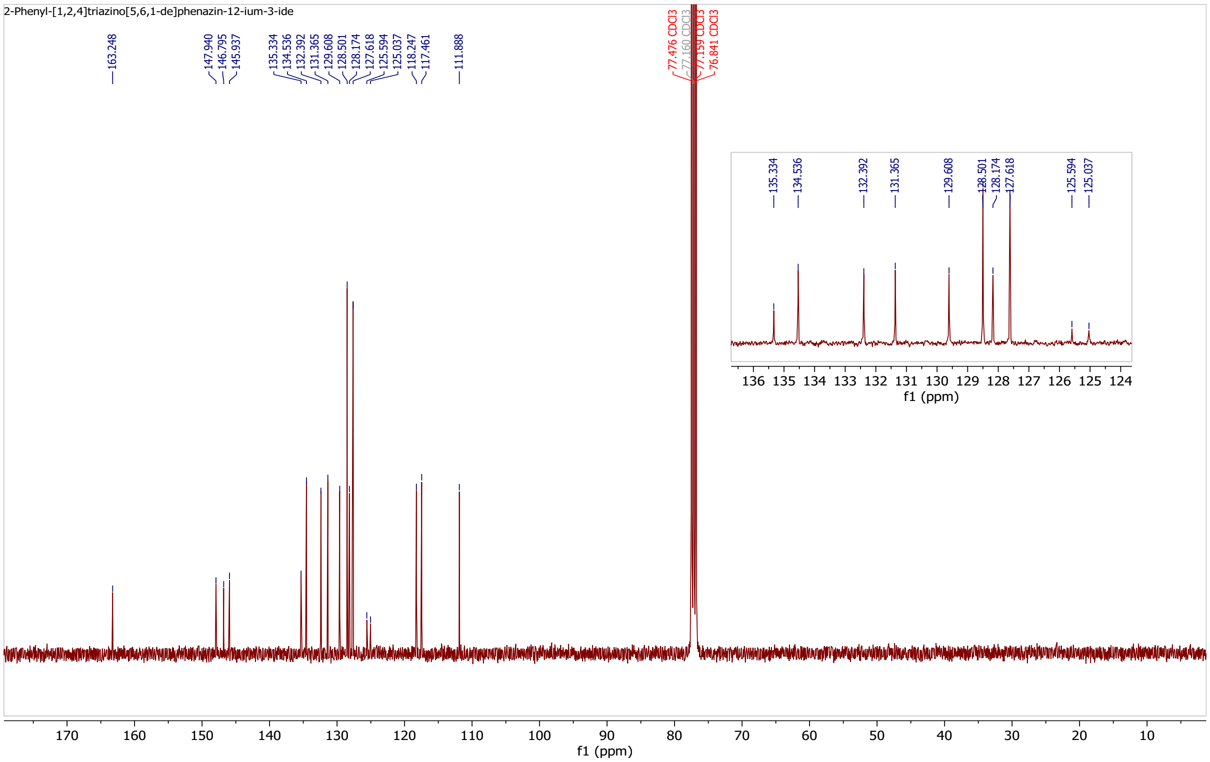

Figure S1. ^{1}H NMR (400 MHz) and ^{13}C{^{1}H} NMR (101 MHz) spectra of **2** ($CDCl_3$).

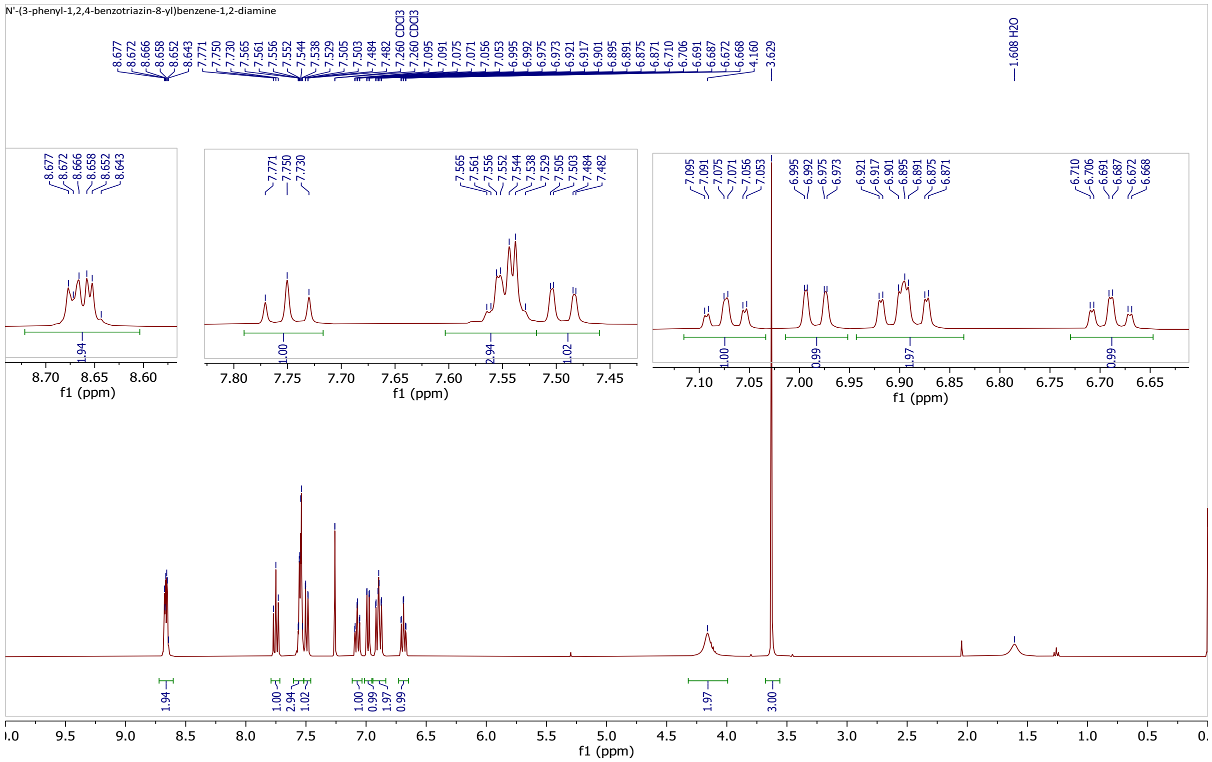

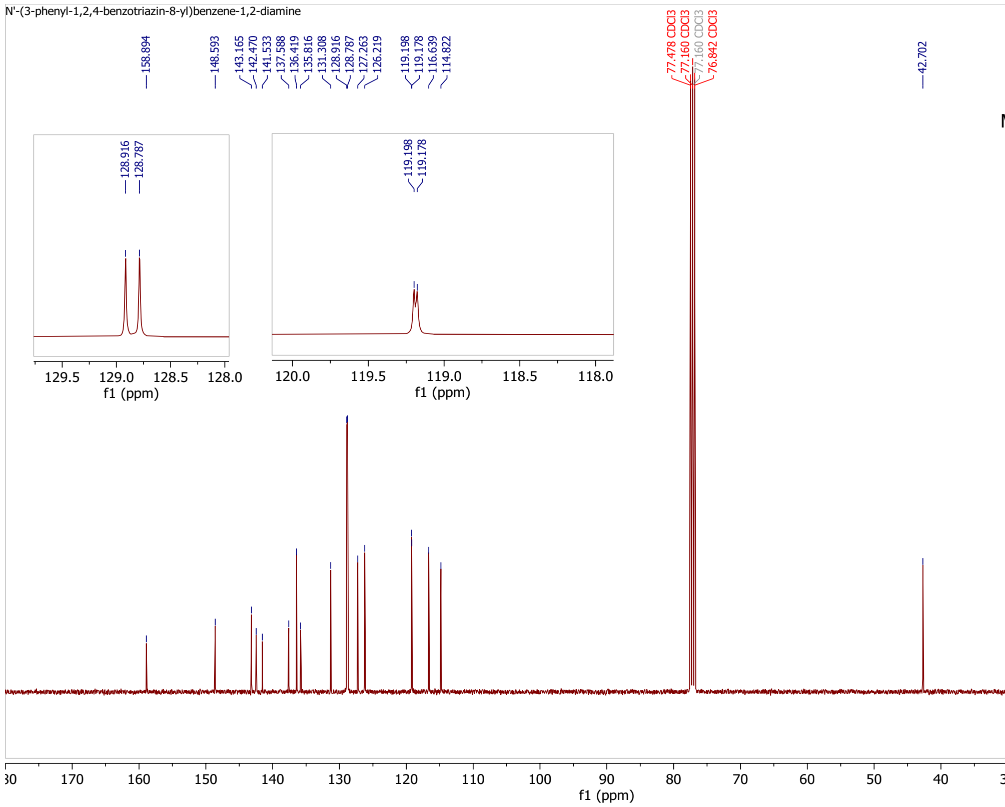

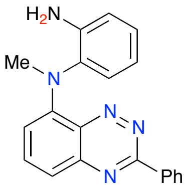

Figure S2. ^{1}H NMR (400 MHz) and ^{13}C{^{1}H} NMR (101 MHz) spectra of **3b** ($CDCl_3$).

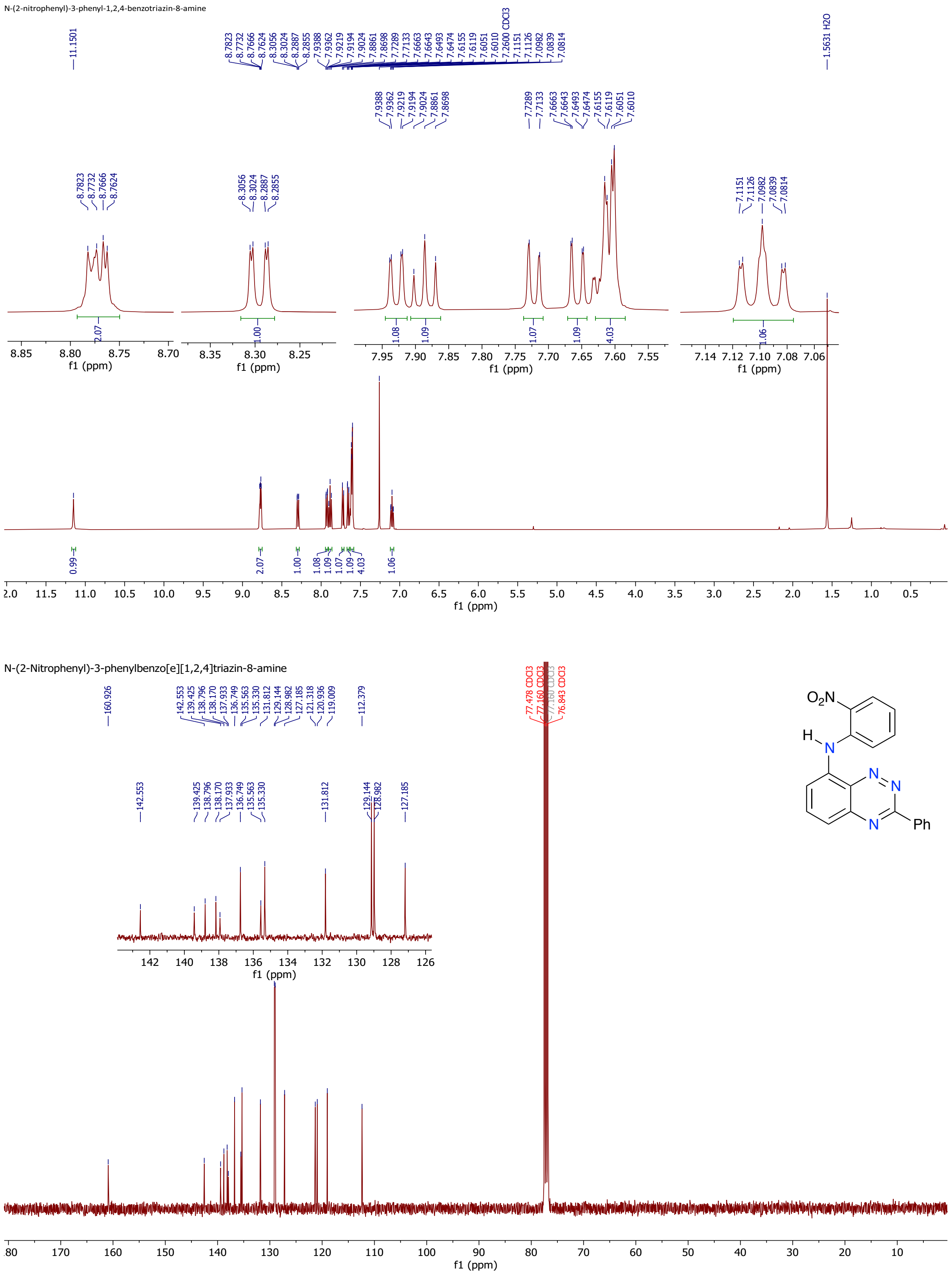

Figure S3. ^{1}H NMR (400 MHz) and $^{13}C\{^1H\}$ NMR (101 MHz) spectra of **4a** ($CDCl_3$).

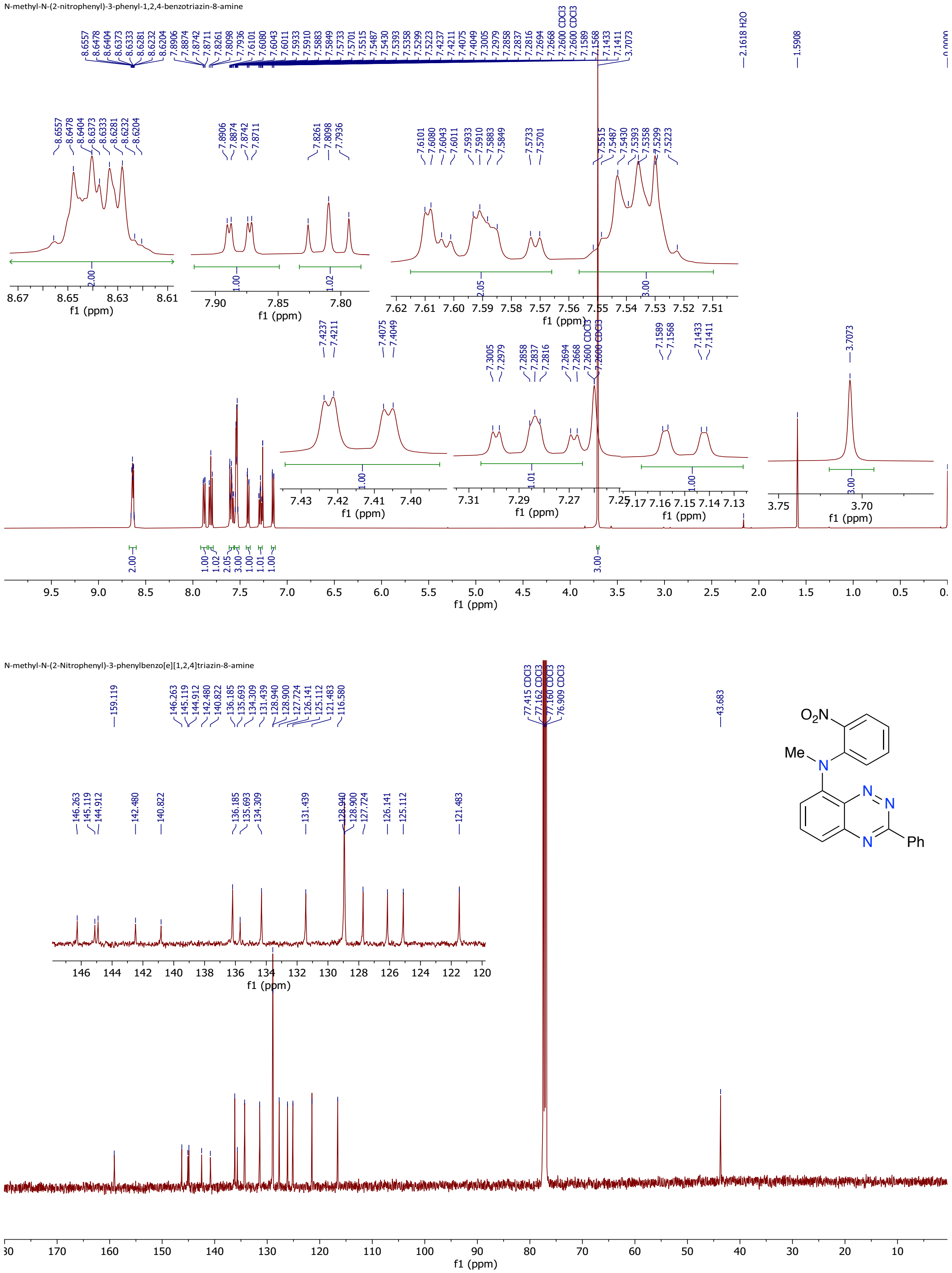

Figure S4. ^{1}H NMR (400 MHz) and ^{13}C{^{1}H} NMR (101 MHz) spectra of **4b** ($CDCl_3$).

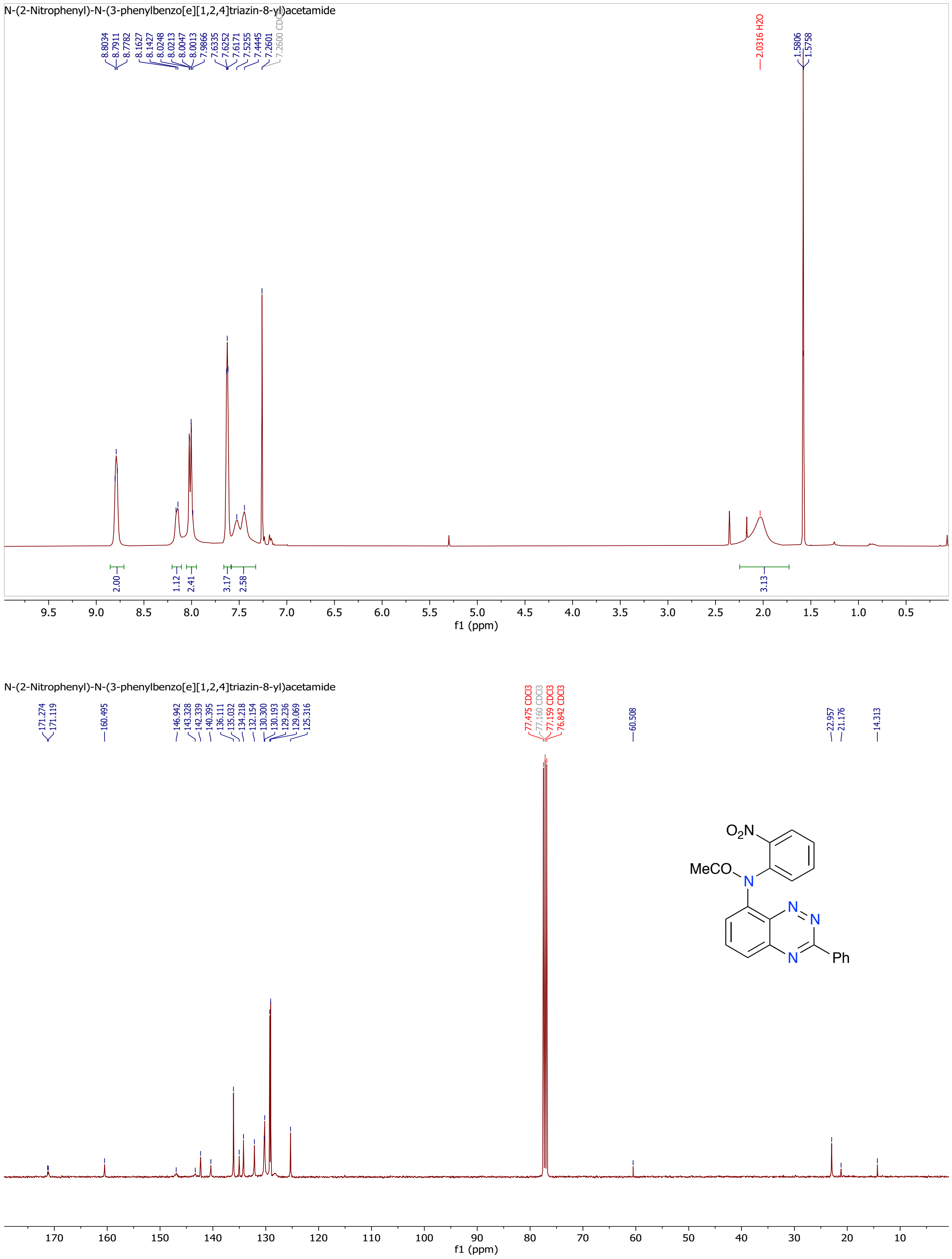

Figure S5. ^{1}H NMR (400 MHz) and $^{13}C\{^{1}H\}$ NMR (101 MHz) spectra of **4c** ($CDCl_3$).

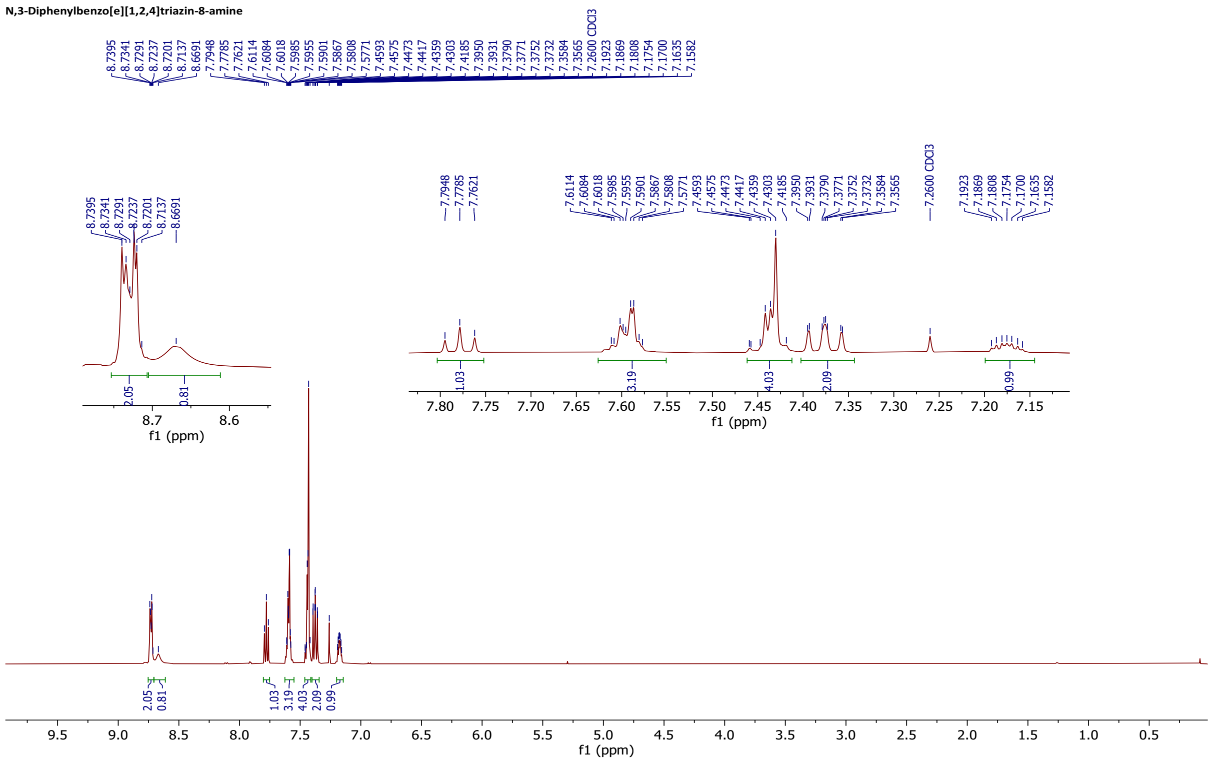

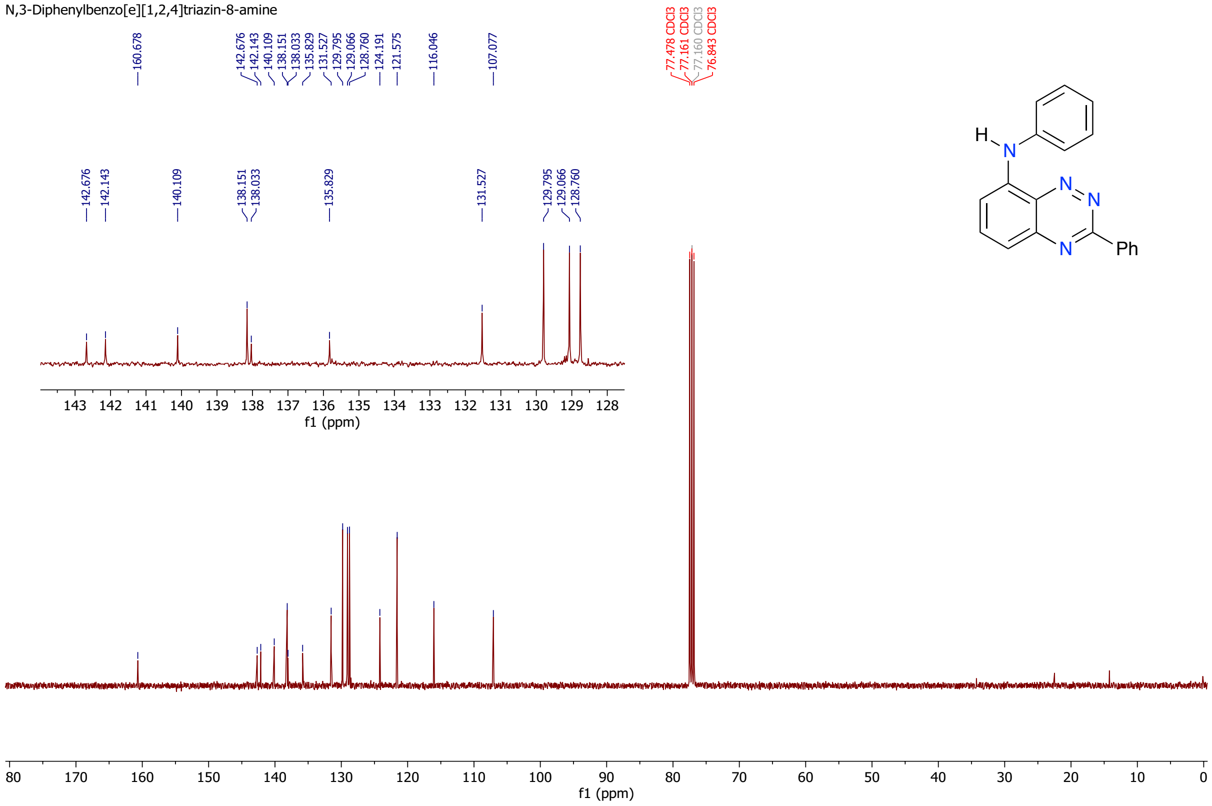

Figure S6. ^{1}H NMR (400 MHz) and ^{13}C{^{1}H} NMR (101 MHz) spectra of **5a** ($CDCl_3$).

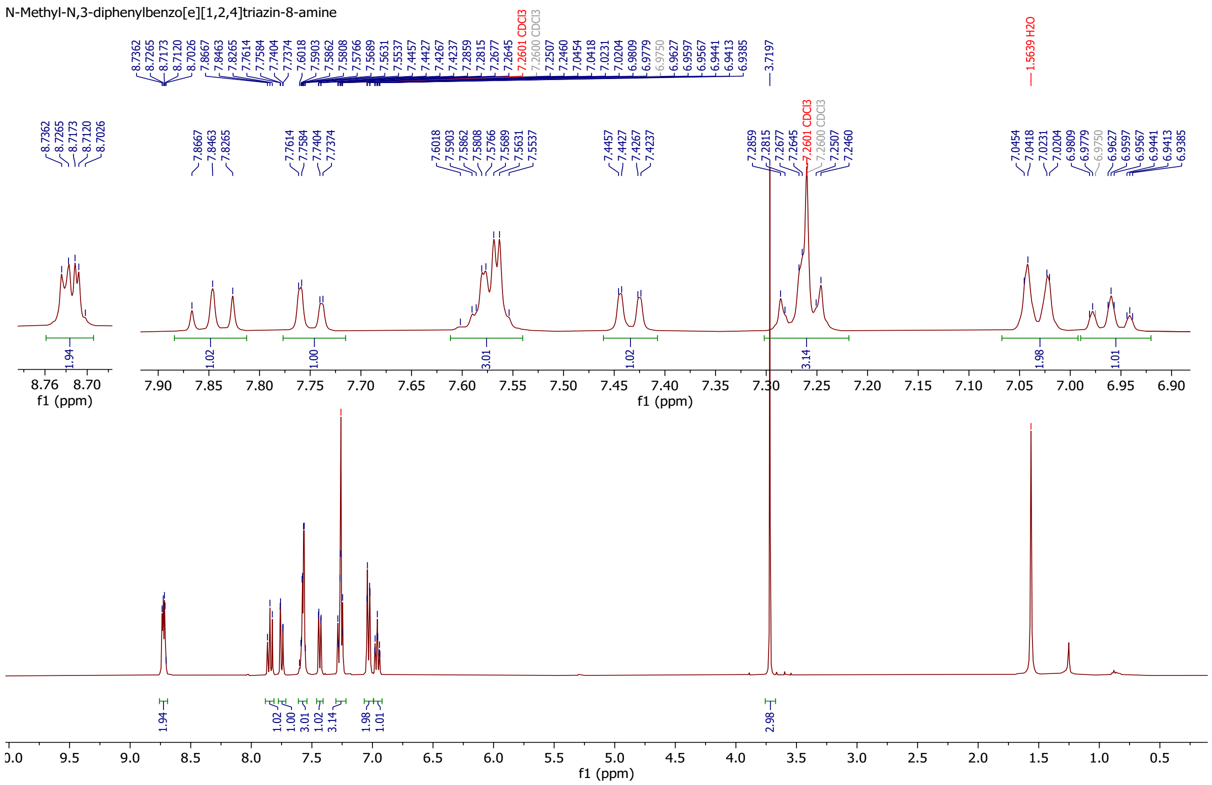

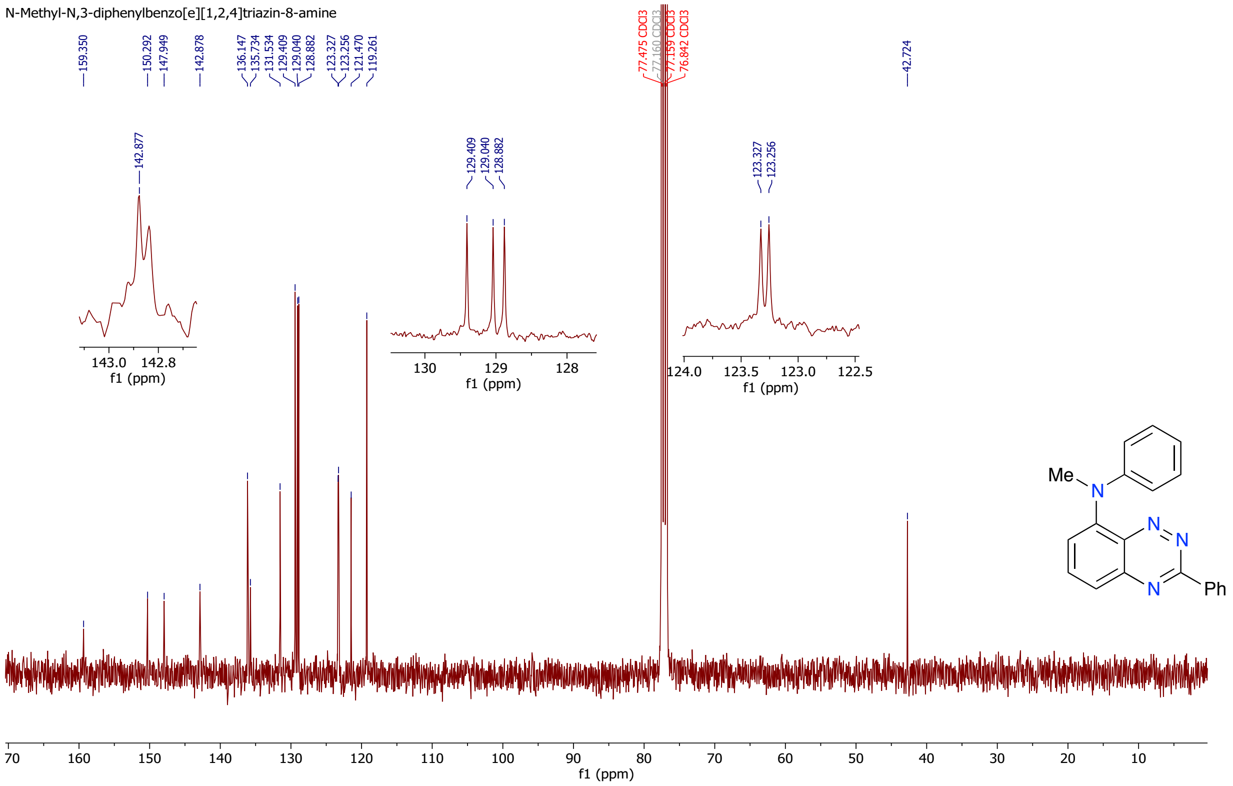

Figure S7. ^{1}H NMR (400 MHz) and ^{13}C{^{1}H} NMR (101 MHz) spectra of **5b** ($CDCl_3$).

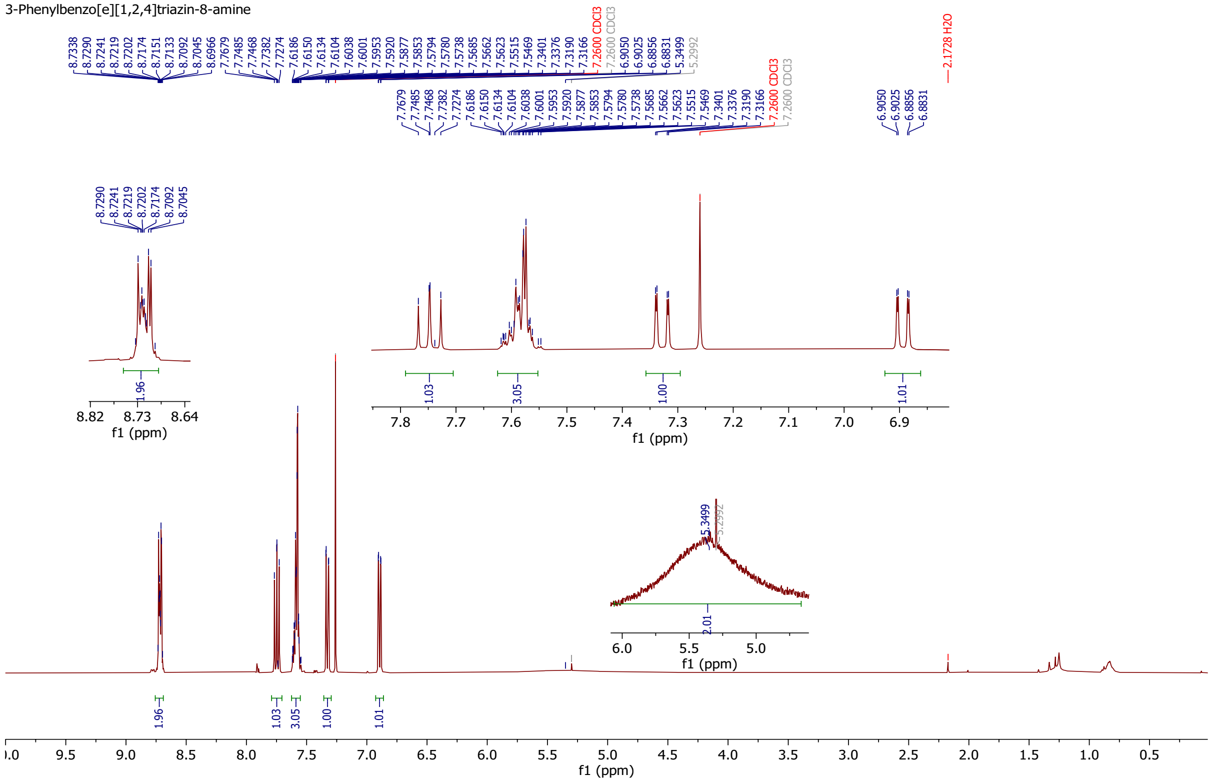

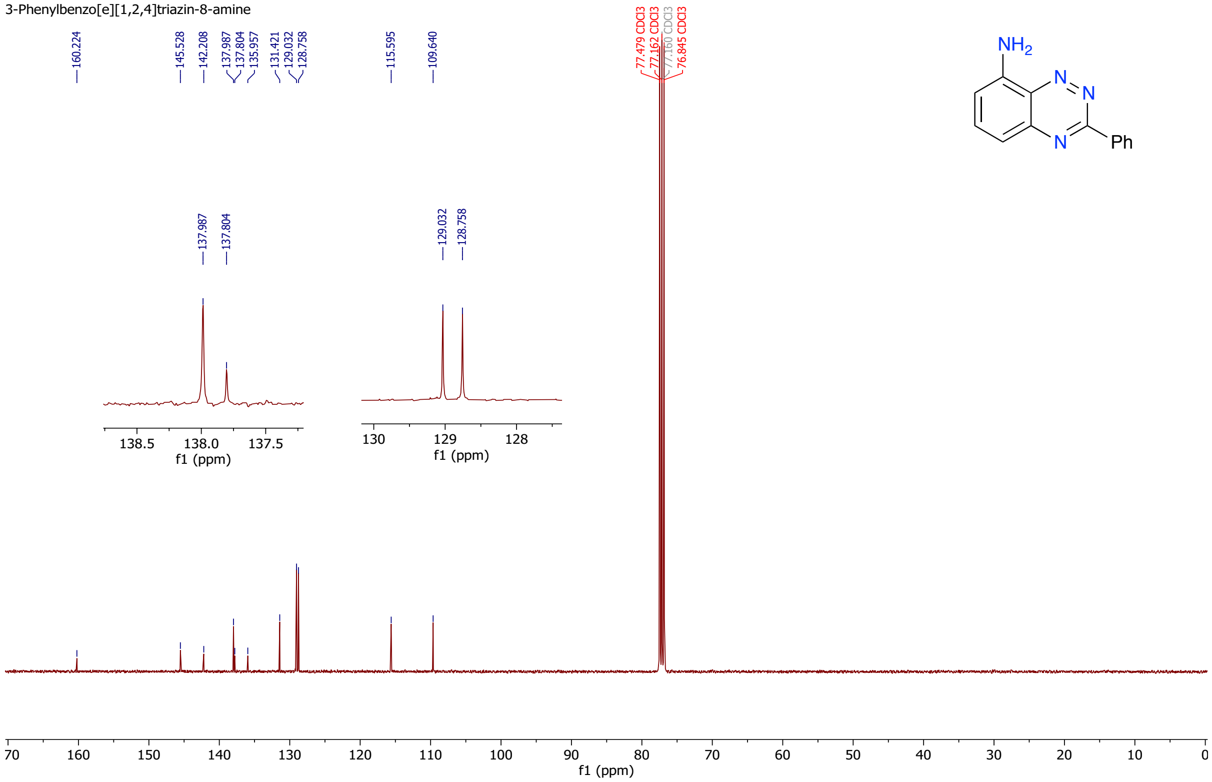

Figure S8. ^{1}H NMR (400 MHz) and ^{13}C{^{1}H} NMR (101 MHz) spectra of **7** ($CDCl_3$).

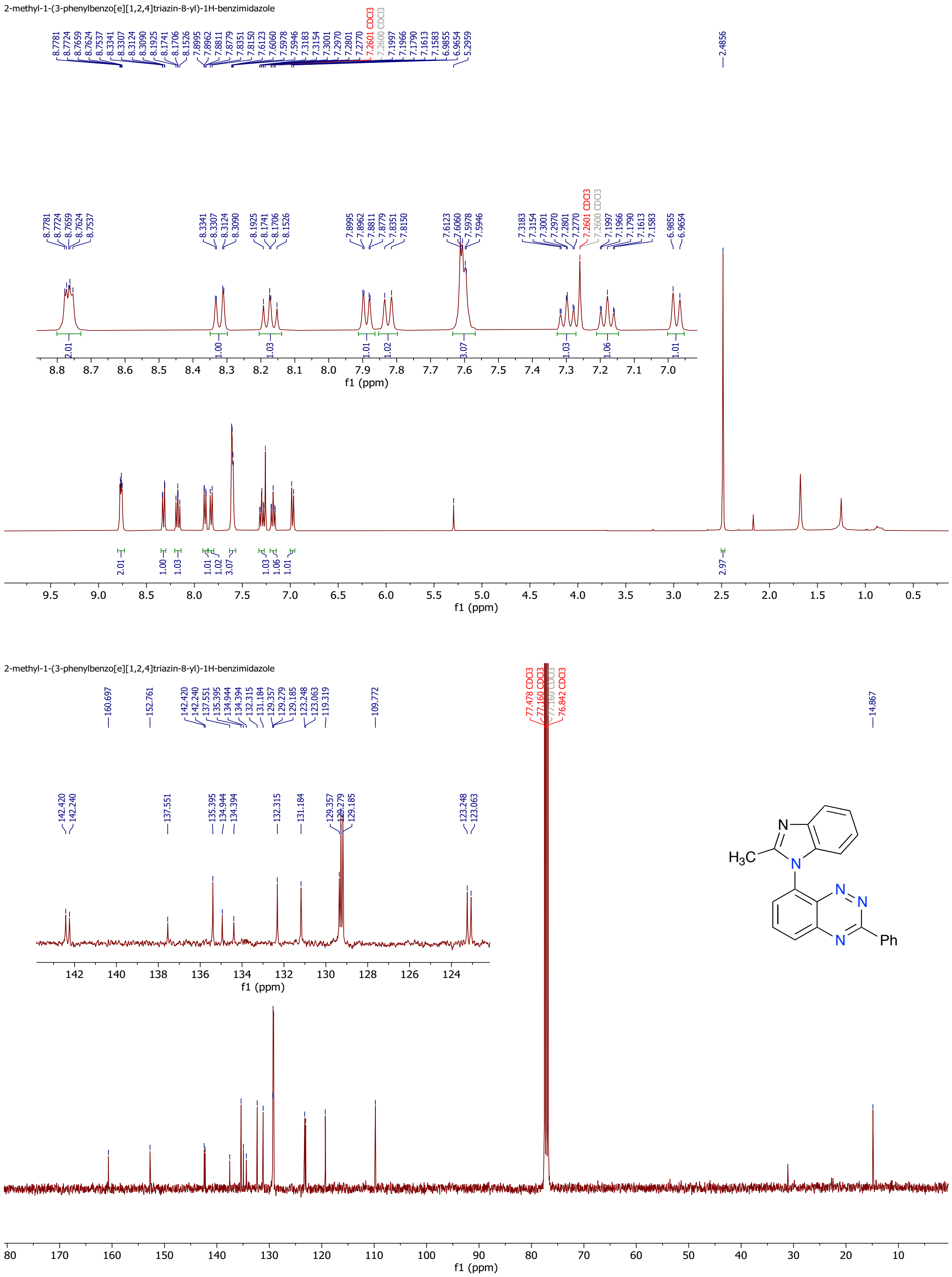

Figure S9. ^{1}H NMR (400 MHz) and ^{13}C{^{1}H} NMR (101 MHz) spectra of **8** ($CDCl_3$).

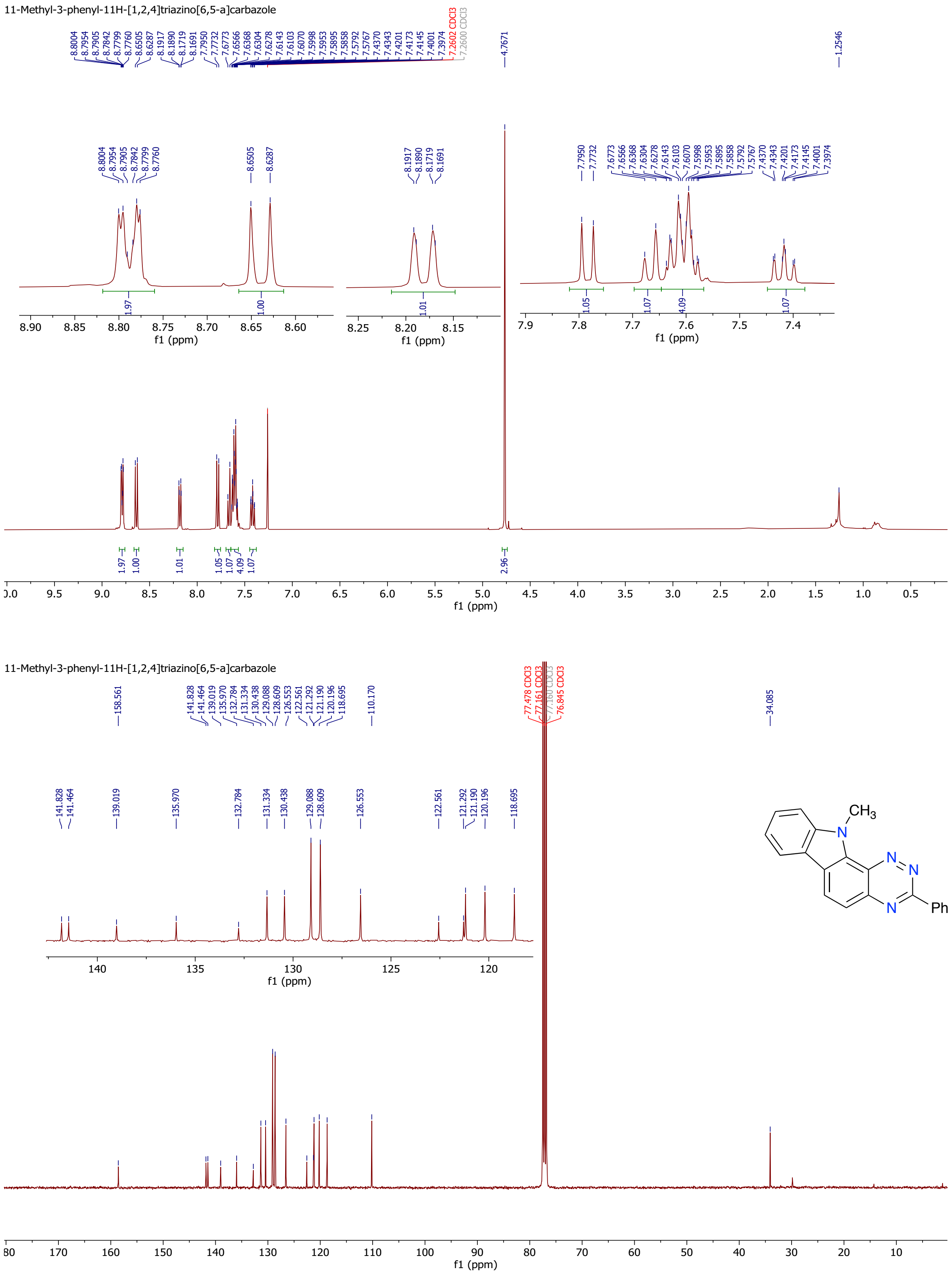

Figure S10. ^{1}H NMR (400 MHz) and ^{13}C{^{1}H} NMR (101 MHz) spectra of **9** ($CDCl_3$).

2. XRD data collection and refinement

Single-crystal XRD measurements for **9** were performed with a Rigaku XtalAB Synergy, Pilatus 300K diffractometer. The measurement was conducted at 100(2) K using the CuK_α radiation (λ = 1.54184 Å). The data was integrated using CrysAlisPro program.[1] Intensities for absorption were corrected using multi-scan method as in SCALE3 ABSPACK scaling algorithm implemented in CrysAlisPro program.

CCDC: File 2288470 contains the supplementary crystallographic data for this paper. These data can be obtained free of charge from The Cambridge Crystallographic Data Centre via www.ccdc.cam.ac.uk/structures.

Table S1. Selected structural data for 9

	**9**<br>CCDC: 2288470
Formula	C20 H14 N4
Formula Weight	310.35
Crystal System	monoclinic
Space Group	$P2_1$/n
a/Å	16.1098(4)
b/Å	3.8364(1)
c/Å	24.2033(6)
α/°	90
β/°	103.836(2)
γ/°	90
Volume/Å^3	1452.45(6)
Z	4
2θ range for data collection/°	5.972 to 156.13
Index ranges	$-20 \le h \le 17$, $-4 \le k \le 4$, $-28 \le l \le 30$
No. of measured, independent, and observed [$I>2\sigma(I)$] reflections	12087, 2930, 2464
R_{int}	0.0267
Goodness-of-fit on F^2	1.038
Final R indexes [$F^2 > 2\sigma(F^2)$]	R_1 = 0.0369, wR_2 = 0.0930
Final R indexes [all data]	R_1 = 0.0451, wR_2 = 0.0982
Data/restraints/parameters	2930/0/218
Largest diff. peak/hole Å^{-3}	0.38/-0.16

Structure solution and refinement

The structures were solved with the ShelXT[2] structure solution program using Intrinsic Phasing and refined in the ShelXle[3] by the full-matrix least-squares minimization on F^2 with the ShelXL[4] refinement package. All non-hydrogen atoms were refined anisotropically and C–H hydrogens were generated geometrically using the HFIX command as in ShelXL. Hydrogen atoms were refined isotropically and constrained to ride on their parent atoms.

The crystal data and structure refinement descriptors are presented in Table S1. Partial packing diagrams for **9** is shown in Figures S11 and S12.

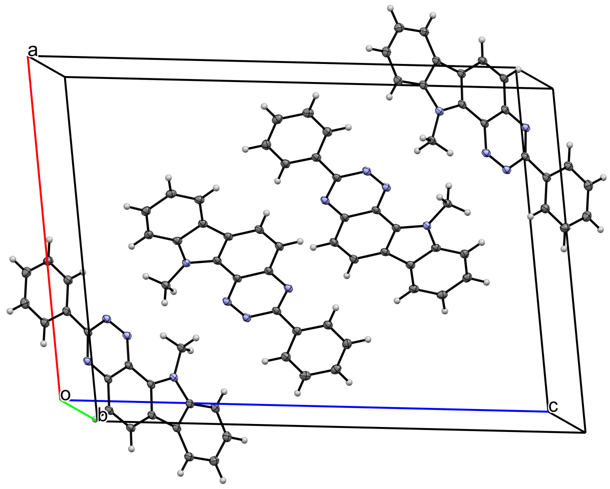

Figure S11. Unit cell packing diagram for **9**.

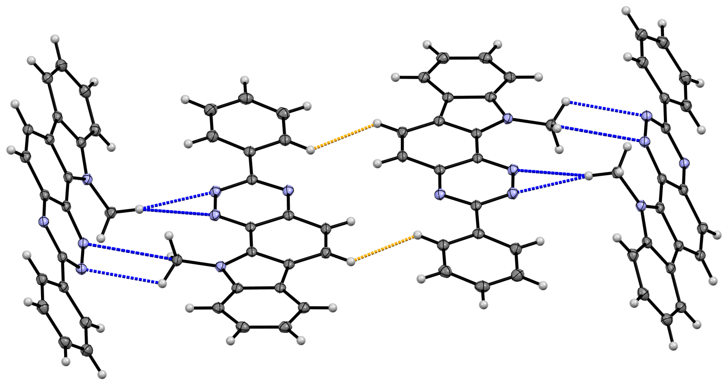

Figure S12. Short contacts (< sum of vdW radii) driving the supramolecular assembly of **9**.

3. UV-vis spectroscopy

UV-vis absorption spectra of zwitterion **2** were recorded on a Perkin-Elmer Lambda-25 UV-vis spectrophotometer. Solutions of **2** were prepared in spectroscopic grade CH_2Cl_2 at concentrations in a range of 1.1 to 4.2×10^{-5} mol·L^{-1}. The measured UV-vis spectra were fitted to the Beer–Lambert law ($A = \varepsilon c l$) and the molar absorption coefficient (ε) was derived from the linear plot. Results are shown in Figure S13.

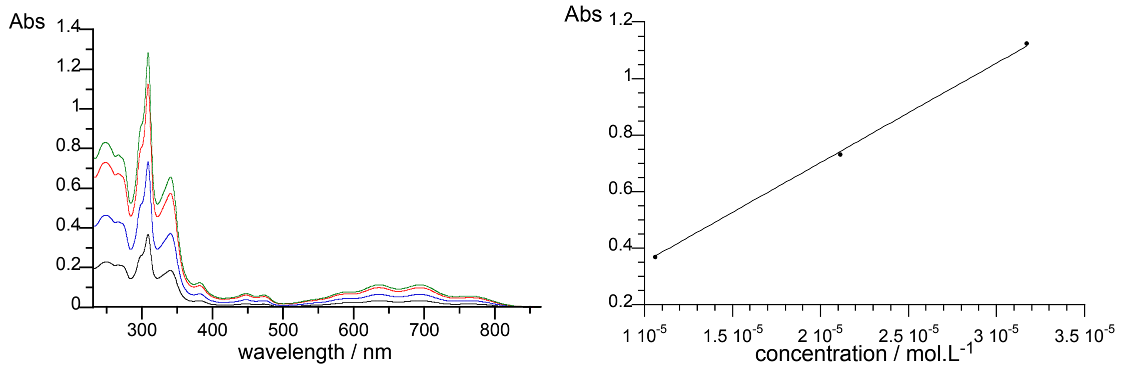

Figure S13. Electronic absorption spectra of **2** in CH_2Cl_2 at four different concentrations (left), determination of molar extinction coefficient ε at λ = 308 nm. Best fit function: ε = 35210(250)×conc, $r^2 = 0.9993$ (right).

4. Electrochemical results

Electrochemical characterization of zwitterion **2** was conducted using a Metrohm Autolab PGSTAT 128N potentiostat/galvanostat instrument. Zwitterion **2** was dissolved in dry, spectroscopic grade CH_2Cl_2 (concentration 1.5 mM) in the presence of $[n\text{-}Bu_4N]^+[PF_6]^-$ as an electrolyte (concentration 100 mM) and the resulting solution was degassed by purging with Ar gas for 20 minutes. A three-electrode electrochemical cell was used with glassy carbon disk as the working electrode (ϕ 2 mm, alumina polished), Pt wire as the counter electrode and Ag/AgCl wire as the pseudoreference electrode. All samples were measured without internal reference followed by measurements with added ferrocene as the internal reference with a scan rate of 50 mV s^{-1} at *ca.* 20 °C. The oxidation potential for the Fc/Fc^+ couple was set at 0.0 V.

Cyclic voltammetry (CV) measurements were started from 0.0 V in the oxidative direction and thee CV plot is shown in Figure 7 in the main text.

5. Computational details

a) geometry optimization and energies

Quantum-mechanical calculations were carried out using Gaussian 09 suite of programs.[5] Geometry optimizations were undertaken at the (U)B3LYP/6-311G(d,p) level of theory in the gas phase using tight convergence limits. Transition states were located using the QST3 method and input geometries from the relaxed scans. Vibrational frequency calculations were used to characterize the nature of the stationary points and to obtain thermodynamic parameters. SCF energies of investigated species were obtained using single point calculations at the (U)B3LYP/6-311++G(d,p)//(U)B3LYP/6-311G(d,p) level of theory in PhCl dielectric medium requested with the "SCRF(Solvent=C6H5Cl)" keyword.[6]

b) spin delocalization

Spin delocalization parameter RDV (Radical Delocalization Value) was calculated according to the formula:[7]

$$RDV = \sum_{i=1}^{n} (\rho_i)^2$$

where spin concentration r_i on each heavy atoms *i* (hydrogen atoms summed up to heavy atoms) is obtained with the UB3LYP/EPR-II // UB3LYP/6-311G(d,p) method in CH_2Cl_2 dielectric medium using the PCM model[8] [keywords: SCRF(Solvent=CH2Cl2)].

For the purpose of this work, an inverse is reported: $RDV^{-1}=1/RDV$, since now larger values corresponds to greater delocalization.

c) electronic excitation data

Electronic excitation energies in CH_2Cl_2 dielectric medium were obtained for radicals **1** and the zwitterion **2** at the (U)B3LYP/6-311++G(d,p) // (U)B3LYP/6-311G(d,p) level of theory, while electronic excitations for precursors **4b**, **4c** and **5b** were obtained at the B3LYP/6-31G(d,p) // B3LYP/6-311G(d,p) level of theory using time-dependent DFT method[9] supplied in the Gaussian 09 package.[5] Solvation models in calculations were implemented by PCM model[6] using the SCRF(solvent=CH2Cl2) keyword.

d) partial output from TD-DFT calculation

```
Open-shell singlet at
UB3LYP/6-311++G(d,p)// UB3LYP/6-311G(d,p) TD(NStates=20) in CH2Cl2
```

Blatter

```
Excited State   1:  2.043-A      2.0937 eV  592.18 nm  f=0.0013  <S**2>=0.793
     75A -> 76A         0.95503
     74B -> 75B        -0.21059
 This state for optimization and/or second-order correction.
 Total Energy, E(TD-HF/TD-KS) =  -896.826343727
 Copying the excited state density for this state as the 1-particle RhoCI density.

 Excited State   2:  2.126-A      2.5816 eV  480.26 nm  f=0.0284  <S**2>=0.880
     75A -> 76A         0.18199
     75A -> 77A        -0.17134
     73B -> 75B        -0.20099
     74B -> 75B         0.90768

 Excited State   3:  2.048-A      2.8754 eV  431.20 nm  f=0.0017  <S**2>=0.799
     75A -> 77A         0.13287
     69B -> 75B        -0.15707
     70B -> 75B        -0.36485
     71B -> 75B         0.80372
     72B -> 75B        -0.16633
     73B -> 75B        -0.32633
     74B -> 75B        -0.11549

 Excited State   4:  2.142-A      2.9550 eV  419.58 nm  f=0.0759  <S**2>=0.897
     75A -> 77A         0.93360
     69B -> 75B        -0.10689
     71B -> 75B        -0.14282
     74B -> 75B         0.14098

 Excited State   5:  2.160-A      3.2821 eV  377.76 nm  f=0.0701  <S**2>=0.916
     75A -> 78A        -0.27864
     69B -> 75B         0.10861
     71B -> 75B         0.34831
     72B -> 75B         0.17811
     73B -> 75B         0.75939
     74B -> 75B         0.18775
     74B -> 76B        -0.18167
```

1O

```
Excited State   1:  2.048-A'     2.0169 eV  614.71 nm  f=0.0019  <S**2>=0.799
     78A -> 79A         0.94994
     76B -> 78B        -0.10720
     77B -> 78B        -0.20214
 This state for optimization and/or second-order correction.
 Total Energy, E(TD-HF/TD-KS) =  -970.866929118
 Copying the excited state density for this state as the 1-particle RhoCI density.

 Excited State   2:  2.078-A'     2.1140 eV  586.50 nm  f=0.0526  <S**2>=0.830
     78A -> 79A         0.19366
     78A -> 80A        -0.10322
     77B -> 78B         0.95417

 Excited State   3:  2.191-A'     2.7401 eV  452.48 nm  f=0.0406  <S**2>=0.950
     77A -> 80A         0.12811
     78A -> 80A         0.94496
     72B -> 78B         0.13319
     77B -> 80B        -0.10497
```

```
 Excited State   4:  2.047-A"     2.9566 eV  419.34 nm  f=0.0011  <S**2>=0.798
     73B -> 78B        0.98747

 Excited State   5:  2.395-A'     3.1100 eV  398.66 nm  f=0.0396  <S**2>=1.184
     76A -> 79A        0.12704
     77A -> 79A        0.29623
     78A -> 79A        0.12698
     76B -> 78B        0.87535
     76B -> 79B       -0.12618
     77B -> 79B       -0.17346
```

1S

```
Excited State   1:  2.089-A      1.9223 eV  644.97 nm  f=0.0451  <S**2>=0.841
     81B -> 82B        0.97739
 This state for optimization and/or second-order correction.
 Total Energy, E(TD-HF/TD-KS) =  -1293.84333215
 Copying the excited state density for this state as the 1-particle RhoCI density.

 Excited State   2:  2.040-A      2.1146 eV  586.33 nm  f=0.0046  <S**2>=0.791
     82A -> 83A        0.95563
     80B -> 82B        0.15621

 Excited State   3:  2.196-A      2.7020 eV  458.87 nm  f=0.0280  <S**2>=0.955
     81A -> 84A        0.12629
     82A -> 84A        0.93578
     77B -> 82B       -0.13798
     80B -> 82B       -0.13600

 Excited State   4:  2.045-A      2.8023 eV  442.44 nm  f=0.0087  <S**2>=0.796
     82A -> 84A        0.16727
     76B -> 82B        0.12852
     77B -> 82B        0.87258
     80B -> 82B        0.38311

 Excited State   5:  2.100-A      2.9513 eV  420.10 nm  f=0.0363  <S**2>=0.852
     81A -> 83A       -0.11235
     82A -> 83A       -0.15190
     77B -> 82B       -0.40902
     80B -> 82B        0.85649

 Excited State   6:  2.318-A      3.1682 eV  391.34 nm  f=0.0183  <S**2>=1.093
     81A -> 83A       -0.24192
     82A -> 83A       -0.10095
     82A -> 85A        0.61168
     82A -> 87A        0.13369
     78B -> 82B       -0.58111
     79B -> 82B       -0.27798
     81B -> 83B        0.16481
```

1N-Ph

```
Excited State   1:  2.037-A      1.8603 eV  666.48 nm  f=0.0627  <S**2>=0.787
     98A -> 99A        0.45885
     97B -> 98B        0.86550
 This state for optimization and/or second-order correction.
 Total Energy, E(TD-HF/TD-KS) =  -1182.11166539
 Copying the excited state density for this state as the 1-particle RhoCI
density.
```

```
 Excited  State     2:    2.059-A          1.8956  eV   654.08  nm   f=0.0036
<S**2>=0.809
     97A -> 99A        0.10581
     98A -> 99A        0.85279
     97B -> 98B       -0.46821

 Excited  State     3:    2.178-A          2.4890  eV   498.13  nm   f=0.0176
<S**2>=0.936
     97A ->100A       -0.10694
     98A ->100A        0.91208
     98A ->102A       -0.29856

 Excited  State     4:    3.250-A          2.9300  eV   423.15  nm   f=0.0100
<S**2>=2.390
     96A -> 99A        0.10833
     97A -> 99A       -0.50953
     97A ->106A        0.11518
     98A -> 99A        0.12182
     98A ->102A        0.29501
     98A ->103A        0.13088
     98A ->104A        0.17397
     96B -> 98B       -0.11429
     96B -> 99B       -0.11196
     97B -> 99B        0.68639
     97B ->107B       -0.12284

 Excited  State     5:    2.281-A          3.0089  eV   412.06  nm   f=0.0327
<S**2>=1.051
     97A -> 99A        0.25043
     98A ->100A        0.20576
     98A ->102A        0.74983
     98A ->103A        0.19626
     98A ->104A        0.22064
     94B -> 98B        0.12127
     95B -> 98B        0.15730
     96B -> 98B        0.34332
     97B -> 99B       -0.15784
```

2

```
Excited State   1:      Singlet-A'     1.8426 eV  672.88 nm  f=0.0760  <S**2>=0.000
      77 -> 78         0.69675
      77 -> 79        -0.10173
 This state for optimization and/or second-order correction.
 Total Energy, E(TD-HF/TD-KS) =  -950.409262760
 Copying the excited state density for this state as the 1-particle RhoCI density.

 Excited State   2:      Singlet-A'     2.9446 eV  421.06 nm  f=0.0659
<S**2>=0.000
      76 -> 78        -0.20053
      77 -> 79         0.65134
      77 -> 80        -0.13066

 Excited State   3:      Singlet-A'     3.3477 eV  370.35 nm  f=0.2096
<S**2>=0.000
      76 -> 78         0.66737
      77 -> 79         0.16943
```

```
 Excited State   4:      Singlet-A"     3.3496 eV  370.15 nm  f=0.0003
<S**2>=0.000
      73 -> 78         0.70379

 Excited State   5:      Singlet-A'     3.4904 eV  355.21 nm  f=0.0338
<S**2>=0.000
      72 -> 78         0.28380
      74 -> 78        -0.31548
      77 -> 79         0.13156
      77 -> 80         0.53821

 Excited State   6:      Singlet-A'     3.5817 eV  346.16 nm  f=0.0178
<S**2>=0.000
      75 -> 78         0.69694

 Excited State   7:      Singlet-A'     3.6761 eV  337.27 nm  f=0.0215
<S**2>=0.000
      72 -> 78         0.35678
      74 -> 78         0.57132
      77 -> 80         0.11153

Precursors at:
B3LYP/6-31G(d,p)// B3LYP/6-311G(d,p) TD(singlets,root=1, NStates=14) in CH2Cl2
```

4b

```
Excited State   1:      Singlet-A      2.4360 eV  508.97 nm  f=0.0193  <S**2>=0.000
      92 -> 94        -0.37507
      93 -> 94         0.58911
 This state for optimization and/or second-order correction.
 Total Energy, E(TD-HF/TD-KS) =  -1195.17233413
 Copying the excited state density for this state as the 1-particle RhoCI density.

 Excited State   2:      Singlet-A      2.6511 eV  467.66 nm  f=0.0280
<S**2>=0.000
      91 -> 94        -0.19118
      92 -> 94         0.55721
      93 -> 94         0.38184

 Excited State   3:      Singlet-A      2.8713 eV  431.80 nm  f=0.0482
<S**2>=0.000
      93 -> 95         0.70192

 Excited State   4:      Singlet-A      3.4027 eV  364.37 nm  f=0.0721
<S**2>=0.000
      91 -> 94         0.60650
      92 -> 94         0.19419
      92 -> 95         0.12487
      93 -> 96         0.24583
```

4c

```
Excited State   1:      Singlet-A      2.5363 eV  488.85 nm  f=0.0026  <S**2>=0.000
      99 ->101         0.62215
      99 ->102         0.13799
     100 ->101         0.28184
 This state for optimization and/or second-order correction.
 Total Energy, E(TD-HF/TD-KS) =  -1308.52039031
 Copying the excited state density for this state as the 1-particle RhoCI density.
```

```
 Excited State   2:      Singlet-A      3.0787 eV  402.71 nm  f=0.0623
<S**2>=0.000
      98 ->101         0.17206
      99 ->101        -0.25015
     100 ->101         0.62544

 Excited State   3:      Singlet-A      3.2445 eV  382.13 nm  f=0.0017
<S**2>=0.000
      99 ->101        -0.16700
      99 ->102         0.43609
     100 ->102         0.51810

 Excited State   4:      Singlet-A      3.3462 eV  370.52 nm  f=0.0150
<S**2>=0.000
      98 ->102        -0.16283
      99 ->102         0.51989
     100 ->102        -0.42807
```

5b

```
Excited State   1:      Singlet-A      2.2847 eV  542.67 nm  f=0.0915  <S**2>=0.000
      82 -> 83         0.70440
 This state for optimization and/or second-order correction.
 Total Energy, E(TD-HF/TD-KS) =  -990.687042771
 Copying the excited state density for this state as the 1-particle RhoCI density.

 Excited State   2:      Singlet-A      2.5849 eV  479.65 nm  f=0.0026
<S**2>=0.000
      81 -> 83         0.70277

 Excited State   3:      Singlet-A      3.2560 eV  380.79 nm  f=0.0401
<S**2>=0.000
      80 -> 83         0.18345
      82 -> 84         0.67452

 Excited State   4:      Singlet-A      3.5436 eV  349.88 nm  f=0.2430
<S**2>=0.000
      80 -> 83         0.67158
      82 -> 84        -0.18257
```

e) selected MO contours

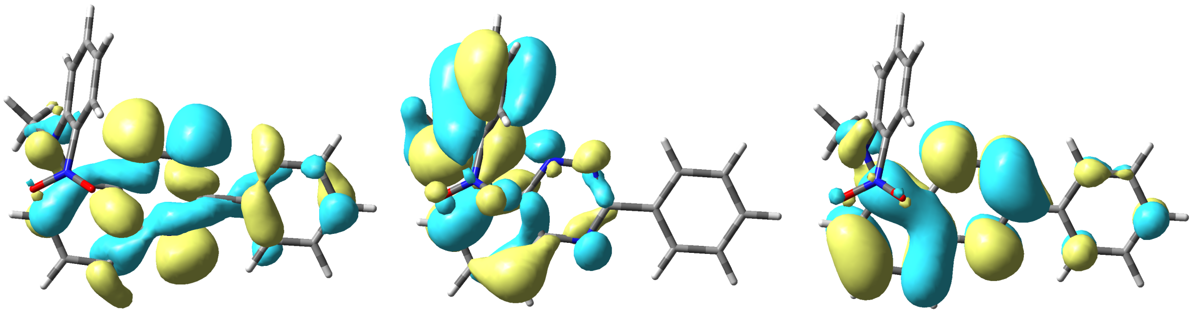

HOMO-1 HOMO LUMO

Figure S14. B3LYP/6-31G(d,p)// B3LYP/6-311G(d,p) derived MO contours for **4b**.

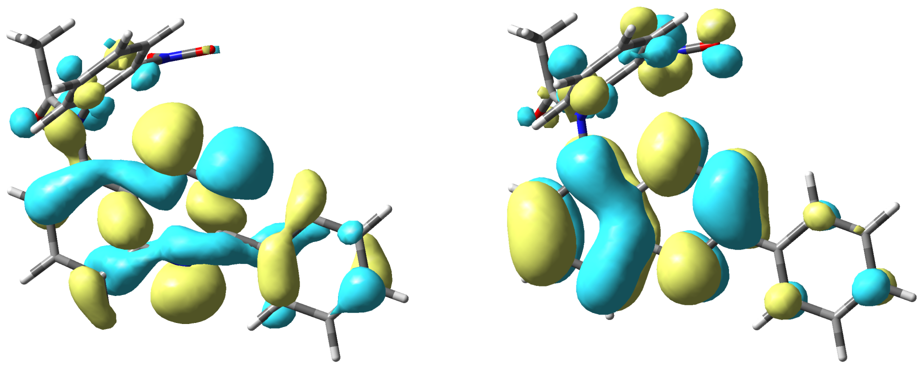

HOMO-1 LUMO

Figure S15. B3LYP/6-31G(d,p)// B3LYP/6-311G(d,p) derived MO contours for **4c**.

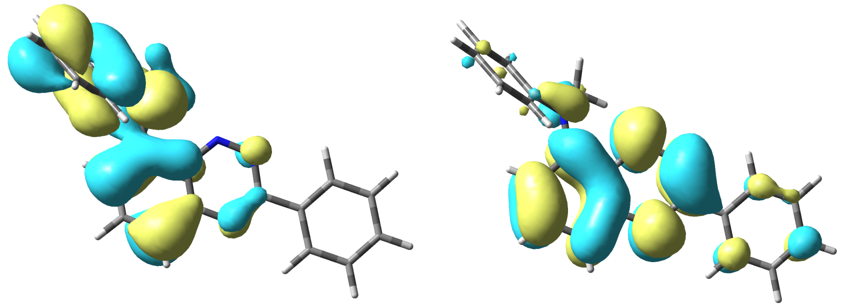

HOMO LUMO

Figure S16. B3LYP/6-31G(d,p)// B3LYP/6-311G(d,p) derived MO contours for **5b**.

f) MO energies obtained with UB3LYP/6-311G(d,p) method

UB3LYP/6-311++G(d,p)//UB3LYP/6-311G(d,p) in CH2Cl2

Blatter

```
Alpha occ. eigenvalues -- -0.27227  -0.26893  -0.26406  -0.24781  -0.18150
Alpha virt. eigenvalues -- -0.06391  -0.03980  -0.03081  -0.02108  -0.01315
Beta occ. eigenvalues --   -0.26740  -0.26520  -0.26076  -0.23806
Beta virt. eigenvalues --   -0.10793  -0.05900  -0.03672  -0.02958  -0.02017
```

1O

```
Alpha occ. eigenvalues --   -0.31212  -0.28692  -0.27930  -0.27734  -0.26529
Alpha occ. eigenvalues --   -0.26221  -0.23253  -0.18127
Alpha virt. eigenvalues --   -0.06869  -0.04389  -0.02868  -0.02054  -0.01202
Beta occ. eigenvalues --   -0.30020  -0.28151  -0.27316  -0.27137  -0.26522
Beta occ. eigenvalues --   -0.25630  -0.22358
Beta virt. eigenvalues --   -0.11264  -0.06493  -0.04010  -0.02388  -0.01937
```

1S

```
Alpha occ. eigenvalues --   -0.28621  -0.27845  -0.27539  -0.26544  -0.25874
Alpha occ. eigenvalues --   -0.22686  -0.18443
Alpha virt. eigenvalues --   -0.06788  -0.04912  -0.03335  -0.02231  -0.01450
Alpha virt. eigenvalues --   24.10429  24.17088  24.17996  24.19248  35.52709
Alpha virt. eigenvalues --   35.58791  35.73098 189.19589
Beta occ. eigenvalues --  -88.88405 -14.43032 -14.35122 -14.31243 -10.26006
```

```
1N-Ph
Alpha occ. eigenvalues --   -0.35003  -0.34065  -0.32857  -0.30526  -0.28135
Alpha occ. eigenvalues --   -0.27722  -0.27720  -0.27077  -0.26909  -0.26326
Alpha occ. eigenvalues --   -0.25562  -0.21375  -0.16967
Alpha virt. eigenvalues --   -0.06357  -0.04278  -0.03649  -0.03189  -0.01919
Beta occ. eigenvalues --   -0.27658  -0.27404  -0.26582  -0.26477  -0.26211
Beta occ. eigenvalues --   -0.24927  -0.20423
Beta virt. eigenvalues --   -0.10384  -0.06006  -0.04057  -0.03650  -0.02983
```

```
2
Alpha occ. eigenvalues --   -0.29302  -0.28099  -0.27793  -0.27523  -0.26772
Alpha occ. eigenvalues --   -0.25847  -0.20243
Alpha virt. eigenvalues --   -0.11692  -0.07051  -0.04845  -0.02412  -0.01909
```

g) modeling cyclization reactions in the excited state

Barriers to cyclization reactions were assessed for model derivatives, in which the C(3)–Ph in **4** and **5** was replaced with C(3)–H in **4’** and **5’** in the 1st excited state (S_1) in CH_2Cl_2 dielectric medium using relaxed scan of the PES, in which the distance between the connecting atoms was shortened in an increment of 0.04 Å, and B3LYP/6-31G(d,p) method implemented with keywords Opt(ModRedundant, MaxCycle=10) and TD=(singlets, root=1, NStates=14). The initial geometry for the PES scan was obtained by geometry optimization of the model compounds in the S_1 state in CH_2Cl_2 dielectric medium with the same DFT method. The relevant FMOs are shown in Figures S17 and S18, while the geometry of the structure with maximum energy on the PES scan in Figure S19.

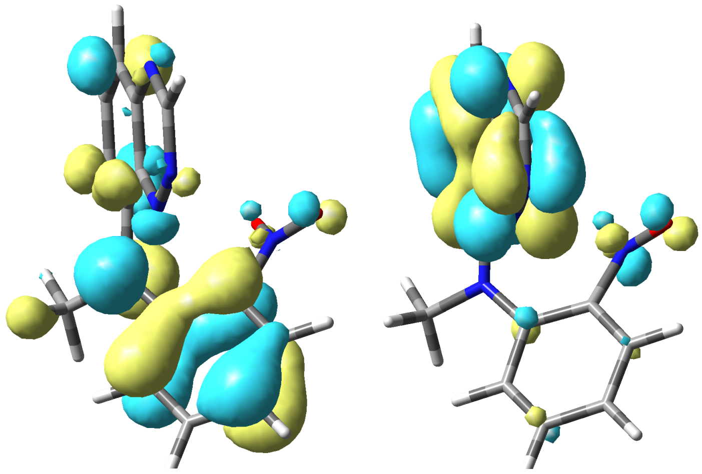

HOMO LUMO

Figure S17. B3LYP/6-31G(d,p) derived FMO contours for model **4b’** with relaxed geometry optimized in the S_1 state.

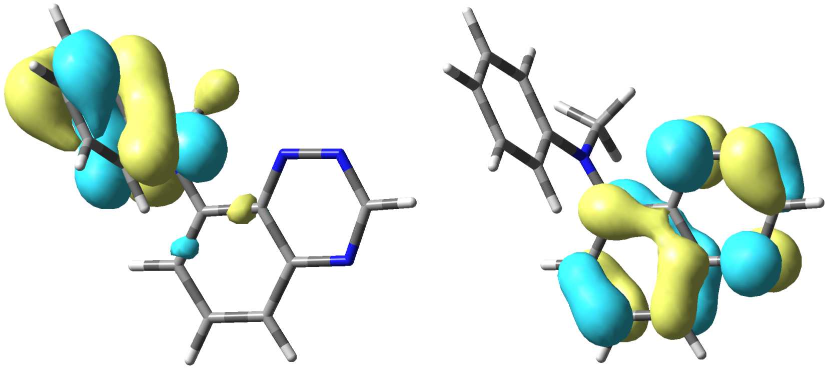

HOMO LUMO

Figure S18. B3LYP/6-31G(d,p) derived FMO contours for model **5b’** with relaxed geometry optimized in the S_1 state.

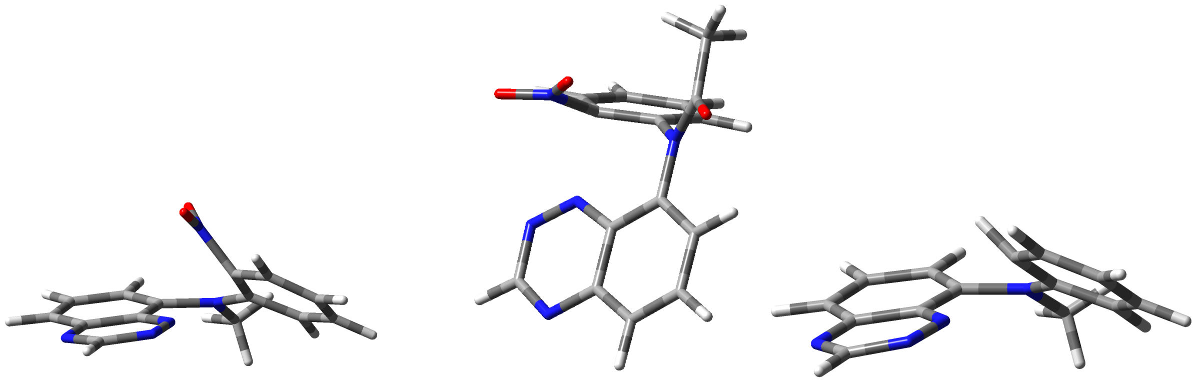

4b’ N(1)···C : 2.063 Å **4c’** N(1)···C : 2.095 Å **5b’** N(1)···C : 2.009 Å

Figure S19. TD-B3LYP/6-31G(d,p) derived FMO geometry for the maximum energy of the S_1 potential energy surface scan for model precursors.

6. Archive for DFT calculations

Blatter


```
1\1\GINC-LOCALHOST\FOpt\UB3LYP\6-311G(d,p)\C19H14N3(2)\PIOTR\18-Aug-20
 23\0\\#P UB3LYP/6-311G(d,p) FOpt(tight) SCF=Direct #P Geom=(NoDistance
 ,NoAngle) fcheck\\Diphenyl Benzotriazinyl\\0,2\N,0.0651685266,0.033601
 2521,-0.099696704\N,0.0231035487,-0.0513936032,1.25808216\C,1.19192726
 29,0.0060184975,1.9010732839\N,2.4004528954,0.1295535316,1.3487867017\
 C,2.449115426,0.1087820781,-0.0169956647\C,1.2687965628,0.0266809161,-
 0.8075958798\C,1.3576667201,-0.0876972779,-2.2011404942\C,2.6036391097
 ,-0.0669410259,-2.8142513853\C,3.7720510331,0.059393823,-2.0525621348\
 C,3.6939570727,0.1396482322,-0.6732978127\C,1.1104298324,-0.063569734,
 3.3859103345\C,-0.1254125094,-0.1029427949,4.0453533261\C,-0.179334605
 2,-0.1696364975,5.4333196952\C,0.9965874851,-0.1971732979,6.1820682904
 \C,2.2288473192,-0.1559643916,5.5323008393\C,2.2874292753,-0.089143532
 2,4.1440971776\C,-1.221419951,0.0582337736,-0.7246557334\C,-2.17921845
 59,-0.8855918586,-0.3497230144\C,-3.4426710651,-0.8504937932,-0.929884
```

2646\C,-3.7562002672,0.1210384256,-1.8793543956\C,-2.8000784103,1.0683
471975,-2.2398128903\C,-1.5337796641,1.0447930399,-1.6621845384\H,4.57
89688421,0.2135277474,-0.053536304\H,4.7380719908,0.0813171178,-2.5432
431616\H,2.6650595057,-0.1547762534,-3.8925853403\H,0.4624017719,-0.19
94040619,-2.7964939193\H,3.2368874534,-0.0546037102,3.6267518852\H,3.1
474043564,-0.1760557179,6.108305346\H,0.9522853447,-0.2486872607,7.264
3787167\H,-1.1415562269,-0.1976555019,5.9326075007\H,-1.0350843989,-0.
0752761882,3.4603012718\H,-0.7985118901,1.796540337,-1.9202885089\H,-3
.0422297396,1.8389735061,-2.9625409241\H,-4.7418768775,0.144675843,-2.
3291997471\H,-4.1836383511,-1.5866642768,-0.6402183086\H,-1.9246249228
,-1.6267505406,0.3962195969\\Version=ES64L-G09RevD.01\State=2-A\HF=-89
6.8857216\S2=0.766193\S2-1=0.\S2A=0.750158\RMSD=5.754e-09\RMSF=6.196e-
07\Dipole=-0.6468206,-0.0435498,-1.0254812\Quadrupole=3.4978461,-8.378
1217,4.8802757,1.0109596,1.1453408,-2.7793456\PG=C01 [X(C19H14N3)]\\@

1O

1\1\GINC-GAUSIANDELL\FOpt\UB3LYP\6-311G(d,p)\C19H12N3O1(2)\PKASZYNSKI\
21-Jul-2023\0\\#P UB3LYP/6-311G(d,p) FOpt=tight SCF=Direct #P Geom=(No
Distance,NoAngle) fcheck\\Parent C(8)-O-Ph(N1) benzotrazinyl (phenazin
oBT), Cs\\0,2\N,-0.2278779588,0.,-0.708360423\N,1.632203919,0.,1.31309
30444\C,0.3129852527,0.,1.5112622984\N,-0.6502990169,0.,0.5768713662\C
,2.8785071969,0.,-2.690204567\C,1.5290863703,0.,-2.3802261775\C,1.1219
160853,0.,-1.0441073004\C,2.0578843729,0.,0.0127353727\C,3.4247314678,
0.,-0.3200921519\C,3.8168628338,0.,-1.6491135769\C,-1.1822316061,0.,-1
.7397717043\C,-0.7290717848,0.,-3.0714325829\C,-1.6371712113,0.,-4.120
1801887\C,-3.0046643111,0.,-3.8561829165\C,-3.4598586091,0.,-2.5385350
028\C,-2.5557153019,0.,-1.4828482891\C,-0.1685145741,0.,2.9197084452\C
,-1.5362027656,0.,3.2244547117\C,-1.9622923621,0.,4.5480779788\C,-1.03
12454766,0.,5.5858451972\C,0.3307687064,0.,5.2902736957\C,0.7608667265
,0.,3.967742324\O,0.6084927733,0.,-3.4015303842\H,4.1445702805,0.,0.48
7965961\H,-1.2509388755,0.,-5.131781866\H,-4.5225765433,0.,-2.32882282
34\H,-2.8833350606,0.,-0.4537989321\H,-2.2565224736,0.,2.4178018371\H,
-3.0234995223,0.,4.7709616663\H,1.0604446267,0.,6.092348288\H,1.814588
9,0.,3.7234413809\H,4.8718916969,0.,-1.8963204375\H,3.1796178507,0.,-3
.7297073762\H,-3.7084292396,0.,-4.6795559916\H,-1.3655953666,0.,6.6174
291244\\Version=ES64L-G09RevD.01\State=2-A"\HF=-970.9223721\S2=0.76517
9\S2-1=0.\S2A=0.750177\RMSD=6.359e-09\RMSF=4.266e-06\Dipole=-0.3139002
,0.,-0.7904145\Quadrupole=6.2141448,-11.0254651,4.8113203,0.,-0.462501
4,0.\PG=CS [SG(C19H12N3O1)]\\@

1S

1\1\GINC-GAUSIANDELL\FOpt\UB3LYP\6-311G(d,p)\C19H12N3S1(2)\PKASZYNSKI\
21-Jul-2023\0\\#P UB3LYP/6-311G(d,p) FOpt=tight SCF=Direct #P Geom=(No
Distance,NoAngle) fcheck\\Parent C(8)-S-Ph(N1) benzotrazinyl (phenazin
oBT), C1\\0,2\N,-0.1444957409,-0.1856678226,-0.7943300867\N,1.64338940
61,0.1721383735,1.2773683909\C,0.3379317813,-0.0318511318,1.4497970237
\N,-0.5792916962,-0.1952388446,0.4929233518\C,3.0946015393,-0.17760782
98,-2.6202085352\C,1.7285647021,-0.3111194711,-2.3919345537\C,1.222021
9765,-0.1430146636,-1.0958707846\C,2.1026544489,0.0920559054,-0.005990
2737\C,3.4806921479,0.2118020403,-0.2672711783\C,3.9634061375,0.086596
2004,-1.5562899146\C,-1.1588739318,-0.0426626022,-1.776930252\C,-0.893
1865016,-0.2556614895,-3.1400326472\C,-1.9121467352,-0.0857414689,-4.0
781501042\C,-3.1941320797,0.27279841,-3.6787124571\C,-3.4573154193,0.4
86907384,-2.3272432065\C,-2.4488167167,0.340720887,-1.3850277386\C,-0.
179526032,-0.0711546579,2.8445085302\C,-1.5030252547,-0.440222024,3.11
88630671\C,-1.966265017,-0.4730517534,4.4296156841\C,-1.1176699211,-0.
1351742636,5.4829768106\C,0.2004538362,0.231335024,5.2169996868\C,0.66
92538403,0.2605605189,3.9078008675\S,0.6824772632,-0.8221186657,-3.731

```
8812377\H,4.1342161236,0.3935915698,0.5760751604\H,-1.6903784135,-0.24
10460503,-5.1277572473\H,-4.4487810303,0.779003942,-2.0023681083\H,-2.
6337128722,0.5153654929,-0.3361729745\H,-2.1564743603,-0.7084283603,2.
2993749008\H,-2.9911485347,-0.7648715569,4.6301645114\H,0.8659661039,0
.4937364815,6.0318320167\H,1.6914518925,0.5380052309,3.6875455366\H,5.
0249553076,0.1855356295,-1.7503737463\H,3.4792466604,-0.2882651386,-3.
6269177962\H,-3.9756345857,0.3928757601,-4.4193035455\H,-1.4810313237,
-0.1601310554,6.5043608497\\Version=ES64L-G09RevD.01\State=2-A\HF=-129
3.8957795\S2=0.765877\S2-1=0.\S2A=0.750187\RMSD=5.905e-09\RMSF=1.446e-
06\Dipole=-0.2810743,0.1629985,-0.6362039\Quadrupole=7.1944052,-11.156
3669,3.9619618,-0.0511991,-0.4397056,0.0867872\PG=C01 [X(C19H12N3S1)]\
\@
```

1N-a

```
1\1\GINC-GAUSIANDELL\FOpt\UB3LYP\6-311G(d,p)\C19H13N4(2)\PKASZYNSKI\21
-Jul-2023\0\\#P UB3LYP/6-311G(d,p) FOpt=tight freq(noraman, ReadIso) S
CF=Direct #P Geom=(NoDistance,NoAngle) fcheck\\Parent C(8)-NMe-Ph(N1)
benzotrazinyl (phenazinoBT), C1\\0,2\N,-0.4676763178,-0.2829774949,-0.
0477273425\N,1.4844486948,1.660984759,-0.0339293413\C,1.7317832992,0.3
506759739,-0.0194273804\N,0.8391862203,-0.6472974971,-0.0248427677\C,-
2.5572099938,2.7650006683,-0.1045081489\C,-2.2143447965,1.4151057956,-
0.0882303169\C,-0.8550455858,1.0594194523,-0.0646211875\C,0.1676696667
,2.0321080856,-0.0570181665\C,-0.2088480589,3.3885757923,-0.073862439\
C,-1.548849747,3.7357081655,-0.0971074564\C,-1.4346680202,-1.307511843
3,-0.0540627292\C,-2.8012794163,-0.9469414854,-0.0778229739\C,-3.77004
38417,-1.9516796739,-0.0843473669\C,-3.3993816005,-3.2932414959,-0.067
7200333\C,-2.0514499797,-3.6441858749,-0.0443233896\C,-1.0726457815,-2
.6558525795,-0.0375118257\C,3.1591392184,-0.0748538302,0.0057937207\C,
3.5192636255,-1.4288570378,0.0223879748\C,4.8588821493,-1.8012124647,0
.0458370885\C,5.8583121606,-0.8291401119,0.0530735462\C,5.5077552271,0
.5197006988,0.0366674456\C,4.168905009,0.8957263554,0.0132004987\H,0.5
761819748,4.1331957064,-0.0680098166\H,-4.8182107375,-1.6717106401,-0.
1025987996\H,-1.7577029611,-4.6865941115,-0.0313167288\H,-0.0216127411
,-2.9014079546,-0.0194991129\H,2.7421422732,-2.1809635207,0.0167309564
\H,5.1242050463,-2.8526333397,0.0585054452\H,6.2796184189,1.2813641114
,0.0421666142\H,3.8812556075,1.9383868748,0.0002653618\H,-1.8307847403
,4.7822825604,-0.109972958\H,-3.6019115645,3.0544019778,-0.1227841531\
H,-4.1657116942,-4.0590459257,-0.073118469\H,6.9024790895,-1.121494623
3,0.0713681385\N,-3.1539647527,0.3950610239,-0.0941802243\H,-4.1315442
399,0.6365327637,-0.1110448325\\Version=ES64L-G09RevD.01\State=2-A\HF=
-951.0631915\S2=0.764085\S2-1=0.\S2A=0.750158\RMSD=1.518e-09\RMSF=3.56
1e-06\Dipole=-1.7200334,-0.1988658,-0.0245621\Quadrupole=11.94023,1.35
65149,-13.2967449,-3.0652753,0.4094816,-0.1668171\PG=C01 [X(C19H13N4)]
\\@
```

```
Low frequencies ---   -0.0010   -0.0005    0.0009    5.6124    6.0334   11.0869
 Low frequencies ---   23.2630   46.8063   64.0858
```

1N-b

```
1\1\GINC-GAUSIANDELL\FOpt\UB3LYP\6-311G(d,p)\C20H15N4(2)\PKASZYNSKI\20
-Jul-2023\0\\#P UB3LYP/6-311G(d,p) FOpt=tight freq(noraman, ReadIso) S
CF=Direct #P Geom=(NoDistance,NoAngle) fcheck guess=check\\Parent C(8)
-NMe-Ph(N1) benzotrazinyl (phenazinoBT), C1\\0,2\N,0.0446615249,0.7710
861342,0.0268485907\N,1.159898688,-1.7384161801,-0.1134179921\C,-0.148
4376927,-1.5089966206,-0.002255534\N,-0.7613003266,-0.3219286846,0.062
5690301\C,3.6259458913,1.6417141322,-0.10418481\C,2.2443265263,1.81397
02899,0.0098277061\C,1.4323522095,0.6633505849,-0.0278066059\C,1.97315
95939,-0.6383019459,-0.1203515136\C,3.3709244876,-0.7730710251,-0.2086
```

849098\C,4.169264575,0.3545408926,-0.2080705046\C,-0.5673981668,2.0333
54397,-0.0520191994\C,0.2496695747,3.1899595023,-0.0024591643\C,-0.370
8909416,4.4385307427,-0.1270031422\C,-1.7545455078,4.5449027231,-0.254
8055653\C,-2.5467898423,3.4025854939,-0.2780588328\C,-1.9521285519,2.1
495949778,-0.1859978945\C,-1.0497885815,-2.6938486147,0.0496367941\C,-
2.4320929882,-2.5529951753,0.22949461\C,-3.2544974525,-3.6736326585,0.
2711566352\C,-2.7102888807,-4.9498774179,0.1335336185\C,-1.3357388538,
-5.0977222975,-0.0432232176\C,-0.50997453,-3.9791769513,-0.0839031621\
H,3.7821975825,-1.7713886347,-0.2789774043\H,0.2272323385,5.338411486,
-0.1372842081\H,-3.6225029274,3.4798714302,-0.3783390222\H,-2.53569406
49,1.2421265416,-0.2168238508\H,-2.8504050149,-1.5617484117,0.34053252
35\H,-4.3227580819,-3.5514314965,0.4121826768\H,-0.9053423331,-6.08738
08476,-0.1495729024\H,0.5587078237,-4.0777733018,-0.2192518822\H,5.245
0743012,0.2511821115,-0.2920621627\H,4.28565239,2.4968549209,-0.128457
3548\H,-2.2035125186,5.5270132702,-0.3439723335\H,-3.3532637247,-5.822
6171458,0.1653780074\N,1.6319707866,3.0605944769,0.1779161699\C,2.4464
284782,4.2332860916,0.4525511423\H,2.7832028018,4.7384093918,-0.461346
0181\H,1.8763619237,4.9392212614,1.055437471\H,3.3198500444,3.93588865
69,1.0312642359\\Version=ES64L-G09RevD.01\State=2-A\HF=-990.3760624\S2
=0.764347\S2-1=0.\S2A=0.750165\RMSD=4.064e-09\RMSF=1.158e-06\Dipole=0.
485797,1.7184284,0.0998516\Quadrupole=4.6963317,7.8703557,-12.5666874,
5.87971,0.3868391,1.0372416\PG=C01 [X(C20H15N4)]\\@

Low frequencies --- -0.0005 0.0001 0.0006 4.6121 5.3406 8.6593
Low frequencies --- 23.9477 39.2323 70.4639

1N-c

1\1\GINC-GAUSIANDELL\FOpt\UB3LYP\6-311G(d,p)\C21H15N4O1(2)\PKASZYNSKI\
20-Jul-2023\0\\#P UB3LYP/6-311G(d,p) FOpt=tight freq(noraman, ReadIso)
SCF=Direct #P Geom=(NoDistance,NoAngle) fcheck\\Parent C(8)-NAc-Ph(N1
) benzotrazinyl (phenazinoBT), C1\\0,2\N,-0.5480151527,-0.3778541344,0
.0772889418\N,1.3461245753,1.5977649208,-0.1574319839\C,1.6344707114,0
.3075262233,0.0319674646\N,0.7661802871,-0.7088457079,0.1194155625\C,-
2.725268939,2.5662535429,-0.1319641036\C,-2.3305478507,1.2558074883,0.
1209751976\C,-0.9685390099,0.9434787042,0.0334599028\C,0.0219854224,1.
9323514815,-0.1609492379\C,-0.4065723951,3.2595661958,-0.3478882554\C,
-1.759310414,3.5568747327,-0.3543164033\C,-1.4912837629,-1.399539401,-
0.1664908937\C,-2.8631450844,-1.0928711998,-0.0524123829\C,-3.80274145
18,-2.0626754239,-0.3988838289\C,-3.38609958,-3.3279660209,-0.80508123
24\C,-2.0294864665,-3.6336277774,-0.883177493\C,-1.0792137119,-2.66906
70356,-0.5775402217\C,3.0698536944,-0.0695235363,0.1357849695\C,3.4564
553022,-1.3792116518,0.4504067644\C,4.8036249457,-1.7102814306,0.54577
30911\C,5.7822592356,-0.7417137586,0.326646799\C,5.4044787787,0.563125
1767,0.0147228692\C,4.0581966758,0.8993459408,-0.078650175\H,0.3476113
278,4.0208556931,-0.5003832818\H,-4.8519820236,-1.8320598958,-0.313993
8785\H,-1.7075729803,-4.6190633659,-1.1983167338\H,-0.0203835835,-2.86
58167,-0.6592462156\H,2.6944056543,-2.1268441143,0.6254552665\H,5.0909
364495,-2.726145812,0.7930875062\H,6.160574222,1.3213479595,-0.1558320
031\H,3.7508318125,1.9087708475,-0.316883987\H,-2.0843891057,4.5748612
765,-0.5338019583\H,-3.7766099186,2.8184560217,-0.1472302151\H,-4.1297
484778,-4.0739281216,-1.0585506156\H,6.8322511113,-1.0023347597,0.4005
433611\N,-3.2277545279,0.200120757,0.4494126016\C,-4.2315616225,0.3131
89167,1.4368356836\O,-5.0256355647,-0.5819151088,1.6297745057\C,-4.267
7389563,1.5704121979,2.2876946302\H,-3.2915874984,2.0264181585,2.44589
58708\H,-4.9281017327,2.3181945486,1.842306148\H,-4.7037777749,1.27277
8442,3.2403784243\\Version=ES64L-G09RevD.01\State=2-A\HF=-1103.7441042
\S2=0.765746\S2-1=0.\S2A=0.750183\RMSD=2.670e-09\RMSF=1.007e-06\Dipole
=-0.1419202,0.5035106,-0.2113463\Quadrupole=-1.6240564,8.6580597,-7.03

40033,-7.9872084,3.2326574,3.1201469\PG=C01 [X(C21H15N4O1)]\\@

Low frequencies --- -3.4452 -0.0005 -0.0003 -0.0001 0.3399 3.2592
Low frequencies --- 31.8328 34.2836 55.1197

1N-d
1\1\GINC-GAUSIANDELL\FOpt\UB3LYP\6-311G(d,p)\C25H17N4(2)\PKASZYNSKI\21
-Jul-2023\0\\#P UB3LYP/6-311G(d,p) FOpt=tight freq(noraman, ReadIso) S
CF=Direct #P Geom=(NoDistance,NoAngle) fcheck\\Parent C(8)-NPh-Ph(N1)
benzotrazinyl (phenazinoBT), C1\\0,2\N,-0.4617364288,-0.2811913257,0.1
385321322\N,1.4897065526,1.6565860367,0.0556603119\C,1.7343361586,0.34
83107434,-0.0040954128\N,0.8412832282,-0.6470513906,0.0303742932\C,-2.
5318085779,2.7678744335,0.3888906285\C,-2.2072692798,1.4119885922,0.32
17056203\C,-0.8499611947,1.0562391542,0.2098519146\C,0.1767302539,2.02
69854934,0.1646977966\C,-0.1862612988,3.3837548629,0.234760923\C,-1.51
95607445,3.733329758,0.3446395289\C,-1.4256833859,-1.3041890688,0.1773
773167\C,-2.7928617075,-0.955533262,0.2897374417\C,-3.7396683152,-1.98
47032514,0.3271159118\C,-3.3496822181,-3.3192870758,0.2557606196\C,-2.
0032038162,-3.6524463504,0.1454271343\C,-1.0455092404,-2.6467231733,0.
1064963058\C,3.1571442258,-0.0773484144,-0.123027854\C,3.5121952673,-1
.4307969654,-0.192380683\C,4.8470972457,-1.8045684706,-0.303151105\C,5
.8470717165,-0.8340228153,-0.3463501442\C,5.5015423025,0.5144487009,-0
.2778609636\C,4.1673131564,0.8917828025,-0.1670265195\H,0.6031893173,4
.1229324721,0.1998109044\H,-4.7876945047,-1.73444969,0.4127077094\H,-1
.694700075,-4.6892641558,0.0895835237\H,0.0068338097,-2.8701032711,0.0
214424004\H,2.7344379441,-2.1814935306,-0.1586489729\H,5.1082385188,-2
.8557932141,-0.3559334851\H,6.2737931555,1.275098868,-0.3108686617\H,3
.8837706523,1.9342778821,-0.1128611545\H,-1.7976562683,4.7797224349,0.
3985361365\H,-3.5659090478,3.0689102514,0.4750644556\H,-4.1066622254,-
4.0939809931,0.2871759769\H,6.8876504226,-1.1269914615,-0.4327488597\N
,-3.172183437,0.3945492717,0.3607971389\C,-4.5620507386,0.7427573011,0
.4748410449\C,-5.1439461577,0.8733797719,1.7354880872\C,-5.3225640975,
0.9501068455,-0.6756037233\C,-6.4912738411,1.2105465792,1.8436065793\C
,-6.6695345777,1.2871202341,-0.5626705829\C,-7.254472234,1.4168786141,
0.6957519582\H,-4.5388268747,0.7095725114,2.6193756633\H,-4.8549227231
,0.8453553779,-1.6475082493\H,-6.9435906179,1.3116984329,2.8234029232\
H,-7.2606772278,1.4479062275,-1.4568301423\H,-8.3027608815,1.678842056
3,0.7817469426\\Version=ES64L-G09RevD.01\State=2-A\HF=-1182.1579619\S2
=0.764177\S2-1=0.\S2A=0.750161\RMSD=4.898e-09\RMSF=1.655e-06\Dipole=-2
.1024718,-0.0820397,0.1531312\Quadrupole=10.1563279,-0.9208122,-9.2355
157,-5.0667028,-1.6080129,0.6442727\PG=C01 [X(C25H17N4)]\\@

Full mass-weighted force constant matrix:
Low frequencies --- -5.8887 -4.3938 -0.0010 0.0003 0.0012 1.8637
Low frequencies --- 15.8864 25.4103 40.9301

2
1\1\GINC-GAUSIANDELL\FOpt\RB3LYP\6-311G(d,p)\C19H12N4\PKASZYNSKI\20-Ju
l-2023\0\\#P B3LYP/6-311G(d,p) FOpt=tight freq(noraman, ReadIso) SCF=D
irect #P Geom=(NoDistance,NoAngle) fcheck\\Parent C(8)-N-Ph(N1) benzot
razinyl zwittetion, Cs\\0,1\N,-0.1963047382,0.,-0.7189058732\N,1.63723
87788,0.,1.3137318993\C,0.3351078884,0.,1.5053055588\N,-0.6298249102,0
.,0.5524896417\C,2.9057466013,0.,-2.7020720245\C,1.5167472201,0.,-2.43
41424752\C,1.1265217912,0.,-1.0531482216\C,2.0771650512,0.,0.016228650
3\C,3.4262418411,0.,-0.3172691933\C,3.8176149904,0.,-1.6652265154\C,-1
.151573961,0.,-1.7302727009\C,-0.6823808261,0.,-3.083188016\C,-1.67278
2068,0.,-4.1117264171\C,-3.0063789857,0.,-3.8080093039\C,-3.4408588919

,0.,-2.4571098496\C,-2.5295611002,0.,-1.4306177038\C,-0.1678000376,0.,
2.9052992828\C,-1.5394840726,0.,3.1893402624\C,-1.9844902677,0.,4.5073
618574\C,-1.0681635116,0.,5.557191975\C,0.2987691887,0.,5.2814674042\C
,0.7474995154,0.,3.9660984123\N,0.6106265255,0.,-3.4300665942\H,4.1529
573315,0.,0.4841907369\H,-1.3119006188,0.,-5.13241875\H,-4.5008584967,
0.,-2.2337118039\H,-2.836207308,0.,-0.3957759542\H,-2.2490376089,0.,2.
3734317037\H,-3.0486037679,0.,4.7152776963\H,1.0165206206,0.,6.0940640
037\H,1.8047152013,0.,3.736823545\H,4.87687035,0.,-1.8982943754\H,3.21
43837585,0.,-3.7389968479\H,-3.7423365683,0.,-4.6038777933\H,-1.416801
9147,0.,6.5839677836\\Version=ES64L-G09RevD.01\State=1-A'\HF=-950.4579
044\RMSD=2.579e-09\RMSF=1.420e-06\Dipole=-0.7655366,0.,-0.035137\Quadr
upole=7.6390897,-9.4785725,1.8394828,0.,2.3090139,0.\PG=CS [SG(C19H12N
4)]\\@

Low frequencies --- -8.8127 -0.0005 0.0005 0.0005 3.7598 4.4760
Low frequencies --- 19.9447 49.8515 85.8133
Diagonal vibrational polarizability:

4b

1\1\GINC-LOCALHOST\FOpt\RB3LYP\6-311G(d,p)\C20H15N5O2\PIOTR\19-Oct-202
3\0\\#P B3LYP/6-311G(d,p) FOpt=tight SCF=Direct #P Geom=(NoDistance,No
Angle) fcheck\\3Ph-benzotrazinyl 2-nitro-phenyl-1-NMe opt in GS\\0,1\N
,0.6288564356,1.2524557379,-1.0828587691\N,3.1634733971,1.8011514876,-
0.1254632597\C,2.3823544594,2.6998367429,-0.6938209919\N,1.1280337679,
2.4395018048,-1.1990080993\C,1.6054238072,-2.0317185509,0.1761659624\C
,0.8036878956,-1.0433540566,-0.3540888567\C,1.3517861638,0.2736697264,
-0.4759003742\C,2.6707170513,0.5478080051,-0.0143219195\C,3.4581069094
,-0.4951446949,0.5273694539\C,2.9251573885,-1.7585849216,0.603147128\C
,-1.8097647221,-0.0496654314,0.8332870435\C,-1.6015292574,-0.554732611
3,-0.4705777587\C,-2.5811370625,-0.2224426322,-1.4248846629\C,-2.86448
78005,0.8101197538,1.1326179837\C,-3.6605963378,0.5894632521,-1.111875
9403\C,-3.8028938258,1.1308158836,0.1653476672\N,-0.9897674708,-0.4614
063623,1.9854357198\O,-0.744179355,0.3852801324,2.8370342087\O,-0.6483
980808,-1.6359131693,2.0560918841\H,4.4592493287,-0.2639509147,0.86834
05403\H,3.512194834,-2.5671848739,1.0232087522\H,1.1956534845,-3.02573
07846,0.3007500333\H,-2.9410664494,1.1894161124,2.1426365685\H,-2.4640
011614,-0.5690490035,-2.4416638341\H,-4.383299743,0.8250505931,-1.8849
051473\H,-4.6340426346,1.7816665121,0.4051835945\N,-0.503280216,-1.342
5736903,-0.8205341864\C,-0.5838136936,-2.1730430185,-2.0230108824\H,-1
.5395044388,-2.6990639085,-2.0489988145\H,-0.469708671,-1.5876465567,-
2.9451041725\H,0.2134784924,-2.9137056578,-1.9915938324\C,2.8615915904
,4.0930585888,-0.8324138941\C,2.0599921056,5.070817603,-1.4387721197\C
,4.1315422879,4.4481638878,-0.3558755822\C,2.5240109481,6.3757942165,-
1.5653645251\H,1.0807279972,4.792805767,-1.8039786474\C,4.589285539,5.
7534792504,-0.4849349091\H,4.744469057,3.6887355017,0.1117285485\C,3.7
877483192,6.7215292228,-1.0902717215\H,1.8970357609,7.1247882257,-2.03
57829945\H,5.5726854575,6.0180719795,-0.1129771559\H,4.1467892023,7.73
98249516,-1.1901019071\\Version=ES64L-G09RevD.01\State=1-A\HF=-1195.51
41279\RMSD=7.028e-09\RMSF=2.605e-06\Dipole=0.001289,-0.5013394,-1.2584
024\Quadrupole=6.4119332,6.1112694,-12.5232026,-1.4130758,6.0435932,5.
4995583\PG=C01 [X(C20H15N5O2)]\\@

4c

1\1\GINC-LOCALHOST\FOpt\RB3LYP\6-311G(d,p)\C21H15N5O3\PIOTR\19-Oct-202
3\0\\#P B3LYP/6-311G(d,p) FOpt=tight SCF=Direct #P Geom=(NoDistance,No
Angle) fcheck\\3Ph-benzotrazinyl 2-nitro-phenyl-1-NAc, GS state\\0,1\N
,-0.9193530828,-1.3272429769,-0.4431827103\N,-3.5980335529,-1.67587512
89,0.1242849244\C,-2.8019421479,-2.6464751973,-0.2833811653\N,-1.46712

4555,-2.4897743587,-0.5774161513\C,-1.8566495169,2.0701728433,0.506632
8287\C,-1.0547726503,1.0150618963,0.1258756782\C,-1.6617869269,-0.2709
783712,-0.0203136657\C,-3.0467816192,-0.4498482885,0.2589440578\C,-3.8
339195108,0.6570563899,0.6535048898\C,-3.2361719984,1.8876621032,0.761
0664074\C,1.9768536324,-0.7098309707,0.0405368109\C,1.2605568726,0.365
3382473,0.5922463409\C,1.427295147,0.6196895226,1.9561355654\C,2.84351
11882,-1.4713932362,0.8176180183\C,2.2979707124,-0.1327822278,2.737436
9929\C,3.0168662355,-1.1776159926,2.1640541165\N,1.8406003958,-1.12318
27746,-1.373394914\O,2.0118253505,-2.3061284753,-1.6199408162\O,1.5948
038693,-0.2630141577,-2.2065974982\H,-4.8868639984,0.4999789496,0.8500
084335\H,-3.8241629137,2.7496782776,1.0546734835\H,-1.4304279932,3.059
1370838,0.5913035662\H,3.3539232821,-2.3025048978,0.3512699612\H,0.857
0394942,1.4293596579,2.3958167904\H,2.4130021058,0.0990887687,3.789717
1166\H,3.7000034809,-1.7696789224,2.7606859737\N,0.3377489872,1.191950
4619,-0.1321664876\C,0.7700650484,2.283224585,-0.9181836201\O,-0.02542
57864,3.002521972,-1.4783874812\C,2.2634380879,2.5174813479,-0.9941809
154\H,2.7240666293,1.7554097191,-1.6237782992\H,2.413805465,3.49506216
44,-1.4477347887\H,2.7391226715,2.4873169008,-0.0121569472\C,-3.345598
4123,-4.0119664057,-0.4512888527\C,-2.5295791738,-5.0544525854,-0.9138
888625\C,-4.6895118713,-4.2748839895,-0.1500743719\C,-3.052855194,-6.3
334037861,-1.0710225417\H,-1.4941165133,-4.8463085183,-1.1470283058\C,
-5.2056109137,-5.5550164192,-0.3081273329\H,-5.3134490187,-3.464960673
9,0.2046990317\C,-4.3893870466,-6.5881211361,-0.7691940547\H,-2.415546
4832,-7.1328501698,-1.4314873428\H,-6.2460841748,-5.7487863629,-0.0729
539952\H,-4.7943242615,-7.5863491379,-0.8932528974\\Version=ES64L-G09R
evD.01\State=1-A\HF=-1308.8939318\RMSD=2.887e-09\RMSF=1.105e-06\Dipole
=0.2517191,0.1930077,2.4102078\Quadrupole=9.0861573,-0.6137382,-8.4724
191,3.1369211,7.2408049,8.8880297\PG=C01 [X(C21H15N5O3)]\\@

5b

1\1\GINC-LOCALHOST\FOpt\RB3LYP\6-311G(d,p)\C20H16N4\PIOTR\19-Oct-2023\
0\\#P B3LYP/6-311G(d,p) FOpt=tight SCF=Direct #P Geom=(NoDistance,NoAn
gle) fcheck\\3Ph-benzotrazinyl phenyl-1-NMe opt in GS\\0,1\N,0.7794974
312,2.0851117506,-1.3334678566\N,3.2291210948,2.1402460104,-0.06461755
83\C,2.7923584657,3.1009419063,-0.8561394128\N,1.5690631826,3.10189142
14,-1.481829315\C,0.6842928362,-1.0859218068,0.4816758816\C,0.27244032
17,-0.0873868063,-0.3881472515\C,1.1568852054,1.0371645999,-0.55760046
75\C,2.411596532,1.0795703611,0.1217064676\C,2.783991555,0.0336910728,
0.9950376405\C,1.9165265885,-1.018532171,1.1652579388\C,-2.3664191342,
-0.4453486124,0.9159716446\C,-2.0809027084,-0.7396281781,-0.4297377205
\C,-2.9703927955,-1.5648736644,-1.1331119585\C,-3.4895287683,-0.975727
0955,1.5336178183\C,-4.1028365725,-2.0826396133,-0.505506488\C,-4.3693
878429,-1.7997626378,0.8288941724\H,3.7381815937,0.0950844431,1.502145
5073\H,2.1844820719,-1.8358305376,1.8257527366\H,0.042148352,-1.943271
8003,0.6363159021\H,-3.690161848,-0.72803536,2.5702008389\H,-2.7818026
558,-1.8128632246,-2.1690204533\H,-4.7749569484,-2.7184478314,-1.07128
35223\H,-5.2500353191,-2.2049131315,1.3129413755\N,-0.9592797523,-0.15
43538927,-1.0605007241\C,-0.9673472331,-0.0539154636,-2.5227621324\H,-
0.7655758432,-1.0229532293,-2.9996463203\H,-1.9420436868,0.3073511807,
-2.8534202563\H,-0.2209292125,0.6667730677,-2.8384455593\C,3.652554896
2,4.2799196913,-1.1067411085\C,3.2211446385,5.3171990677,-1.9457285596
\C,4.9155827679,4.3677020147,-0.5045678853\C,4.0398683862,6.4178772801
,-2.1753309223\H,2.2458867945,5.2456974986,-2.4077399422\C,5.729037893
4,5.4697185275,-0.7376983402\H,5.2417533098,3.5638294758,0.1423199775\
C,5.2944059755,6.4983369739,-1.5737148785\H,3.6974816887,7.2147835842,
-2.8258241216\H,6.7040683599,5.5280270943,-0.2671126942\H,5.9306575803
,7.3575588772,-1.7547199904\H,-1.7079369106,0.2150371271,1.4664231478\
\Version=ES64L-G09RevD.01\State=1-A\HF=-990.9704734\RMSD=8.456e-09\RMS

F=1.613e-06\Dipole=0.1365731,-0.7791055,0.2139714\Quadrupole=-0.309942
2,1.7011056,-1.3911634,6.6867795,1.5637251,-1.9993347\PG=C01 [X(C20H16
N4)]\\@

4b′-S$_1$

1\1\GINC-LOCALHOST\FOpt\RB3LYP TD-FC\6-31G(d,p)\C14H11N5O2\PIOTR\09-Au
g-2023\0\\#P B3LYP/6-31G(d,p) Opt geom(noangle, nodistance) #P TD=(sin
glets,root=1, NStates=14) SCF=Direct SCRF(Solvent=CH2CL2)\\3H-benzotra
zinyl 2-nitro-phenyl-1-NMe opt in 1st exited state\\0,1\N,-0.327233109
5,-0.0819756794,-0.0855307141\N,-2.7727791957,1.3081183045,-0.10788885
92\C,-2.5999675036,0.0490312549,0.2919790642\N,-1.4781196693,-0.680213
572,0.3090765853\C,0.7439848569,3.2339422675,-1.3242223217\C,0.7950282
715,1.8987370889,-0.9134297161\C,-0.3866905291,1.2162419639,-0.4956726
915\C,-1.6172748317,1.9340183774,-0.5179896529\C,-1.6406511804,3.27904
47037,-0.938371396\C,-0.4711422625,3.9227245986,-1.3348481684\C,2.0308
020976,0.1790623312,1.2905811534\C,2.4211580055,0.223584681,-0.0971807
73\C,3.2472203975,-0.8536320189,-0.5462403337\C,2.3872340736,-0.882663
8295,2.1072497805\C,3.609386089,-1.8939885256,0.2839928395\C,3.1715387
79,-1.9251853008,1.616873899\N,1.4068201073,1.3081520434,1.9751089478\
O,0.6801360262,1.0723360164,2.9441611994\O,1.6880323046,2.4399978765,1
.5722445858\H,-2.5914923486,3.8021762579,-0.9170314678\H,-0.4943870379
,4.9621729098,-1.6425290739\H,1.6567921046,3.7526003954,-1.5944913844\
H,2.0635657043,-0.8647529068,3.1396838864\H,-3.4863855637,-0.480596343
8,0.6394989312\H,3.5435175901,-0.893191646,-1.5841840425\H,4.216829468
8,-2.7009084572,-0.1097253323\H,3.4493882327,-2.7441721705,2.270002887
3\N,2.0267722287,1.1727576499,-0.9989832986\C,2.7429471097,1.273429850
1,-2.2841091941\H,3.8170553277,1.1895618526,-2.1261463911\H,2.39888503
61,0.4958469971,-2.9740925906\H,2.5213415404,2.2389034796,-2.729465487
6\\Version=ES64L-G09RevD.01\State=1-A\HF=-964.1802064\RMSD=7.452e-09\R
MSF=1.236e-05\Dipole=4.6280803,-0.6220934,-1.9813323\PG=C01 [X(C14H11N
5O2)]\\@

4c′-S$_1$

1\1\GINC-GAUSIANDELL\FOpt\RB3LYP TD-FC\6-31G(d,p)\C15H11N5O3\PKASZYNSK
I\11-Aug-2023\0\\#P B3LYP/6-31G(d,p) Opt geom(noangle, nodistance) #P
TD=(singlets,root=1, NStates=14) SCF=Direct SCRF(Solvent=CH2CL2)\\3H-b
enzotrazinyl 2-nitro-phenyl-1-NAc optim 2 in 1st exited state\\0,1\N,-
0.2367467952,0.5691977439,2.1282790404\N,0.0000981535,2.6558099661,3.8
31986393\C,-0.3360683406,1.4381166875,4.2510072334\N,-0.4394928591,0.4
430861665,3.36273387\C,0.7258455251,3.2201040115,-0.2595440463\C,0.390
2911344,1.9352978254,0.1992378243\C,0.1285284485,1.7544072132,1.567350
0973\C,0.2347687495,2.8679574418,2.5067859172\C,0.5746201083,4.1249683
944,1.9948787972\C,0.8083296372,4.2913648448,0.6239104506\C,1.03058235
09,-1.4801370483,0.0614499532\C,1.3085826708,-0.2132918607,-0.49553782
16\C,2.6448713849,0.0806459149,-0.7955667481\C,2.0480060737,-2.4145787
984,0.2774993549\C,3.6597917339,-0.8509762329,-0.5940922456\C,3.359579
3592,-2.1071012609,-0.0612754356\N,-0.3167001917,-1.8881322278,0.46592
44164\O,-0.4083431013,-2.7146878781,1.3774696878\O,-1.2885757259,-1.39
93805573,-0.1134179834\H,0.6510935774,4.9565106366,2.6855197487\H,1.06
74634001,5.274427823,0.2454251913\H,0.9117404815,3.3649388205,-1.31691
4415\H,1.7888994056,-3.3720964374,0.7107127814\H,-0.5308807776,1.22225
14295,5.292062026\H,2.8732323944,1.0621289548,-1.1970673665\H,4.681656
7331,-0.596674935,-0.8552263363\H,4.1421646371,-2.8415528912,0.0944564
59\N,0.3319726959,0.8174195125,-0.692972589\C,-0.5017216558,0.87551685
23,-1.8152120294\O,-1.3068496731,1.7859380906,-1.9467712439\C,-0.31978
66167,-0.2095772576,-2.8544963303\H,-0.585183764,-1.1853756288,-2.4425
941505\H,-0.9763294157,0.0180817957,-3.6938173458\H,0.7159226519,-0.25
58091511,-3.2030483348\\Version=ES64L-G09RevD.01\State=1-A\HF=-1077.52

96021\RMSD=6.547e-09\RMSF=7.515e-06\Dipole=3.1931187,-0.0160377,-0.219
1557\PG=C01 [X(C15H11N5O3)]\\@

5b′-S$_1$
1\1\GINC-LOCALHOST\FOpt\RB3LYP TD-FC\6-31G(d,p)\C14H12N4\PIOTR\09-Aug-
2023\0\\#P B3LYP/6-31G(d,p) Opt geom(noangle, nodistance) #P TD=(singl
ets,root=1, NStates=14) SCF=Direct SCRF(Solvent=CH2CL2)\\3H-benzotrazi
nyl phenyl-1-NMe optim in 1st exited state\\0,1\N,0.9357879816,1.11137
00423,-1.2400319182\N,3.5442350057,1.227704607,-0.122579121\C,3.005907
1376,2.1207743108,-0.9632028917\N,1.8010144524,2.139227934,-1.52878702
94\C,0.9698580196,-1.9587972796,0.8195449194\C,0.5563164534,-0.9379734
728,-0.0342820737\C,1.3928816101,0.1498392052,-0.3895058134\C,2.702877
2757,0.1825455785,0.1864680111\C,3.1086245494,-0.8467983371,1.05486192
47\C,2.2543978746,-1.9068070528,1.3683586257\C,-1.708298765,0.18559285
39,1.2400210725\C,-1.8297248715,-0.3997996417,-0.0631583058\C,-3.09693
37906,-0.3413764991,-0.7289687066\C,-2.7986810947,0.7849188107,1.83296
74953\C,-4.1694271195,0.265994848,-0.1137456928\C,-4.0345577371,0.8306
623961,1.1679786334\H,4.1085668925,-0.7926051419,1.4742888854\H,2.5832
866356,-2.6933288474,2.0392936158\H,0.2941260221,-2.7751511782,1.05302
00181\H,-2.6977175543,1.2250157659,2.8181264264\H,3.640858791,2.965483
9924,-1.2338698182\H,-3.2140962336,-0.7562488839,-1.7198457618\H,-5.12
36764869,0.3121930475,-0.6255023021\H,-4.8870121073,1.3045510931,1.640
5940851\N,-0.7595111661,-0.9902989891,-0.6419879955\C,-0.8193734848,-1
.5607340658,-1.9933748186\H,-1.7161338564,-2.1664686215,-2.1237380461\
H,-0.7987475559,-0.7497633788,-2.72690118\H,0.0593220292,-2.1864723579
,-2.1278676407\H,-0.7545769065,0.1477442623,1.7460494029\\Version=ES64
L-G09RevD.01\State=1-A\HF=-759.6857818\RMSD=4.116e-09\RMSF=1.297e-05\D
ipole=-5.6263579,-2.5880215,1.0365258\PG=C01 [X(C14H12N4)]\\@

9
1\1\GINC-GAUSIANDELL\FOpt\RB3LYP\6-311G(d,p)\C20H14N4\PKASZYNSKI\21-Ju
l-2023\0\\#P B3LYP/6-311G(d,p) FOpt=tight freq(noraman, readiso) SCF=D
irect #P Geom=(NoDistance,NoAngle) fcheck\\Carbazole cyclized on the C
7 of BT\\0,1\N,-0.6386950689,-1.2000896903,0.0203180808\N,-2.039918139
4,1.1738848836,0.0963700203\C,-2.613637979,-0.0187959024,0.0484428538\
N,-1.9392116535,-1.2077978087,0.0101442706\C,2.105326232,1.2829287976,
0.1318833293\C,1.4560398536,0.0318598835,0.0812488517\C,0.0305113337,-
0.025337015,0.06831656\C,-0.6938050133,1.2109083581,0.1079614263\C,-0.
0115483619,2.4563053429,0.1586395191\C,1.3598533353,2.4839598368,0.170
0810499\C,3.5202355273,1.0188491835,0.1330000782\C,3.6595975396,-0.390
7343689,0.0825745917\C,4.9138031173,-1.00592368,0.0696445808\C,6.03268
4722,-0.1840869634,0.1081731127\C,5.9127397842,1.2157968989,0.15839200
75\C,4.6655806689,1.8232300713,0.1710923139\C,-4.0923149962,-0.1038169
158,0.0340904321\C,-4.7392690394,-1.3468348172,-0.0162313789\C,-6.1283
145238,-1.4134538858,-0.0292299145\C,-6.8887358575,-0.2459169674,0.007
630263\C,-6.2512690532,0.9935708894,0.0577509823\C,-4.8635736308,1.066
3045854,0.0709625989\H,-0.6083581855,3.3586819363,0.1870595686\H,5.016
4773609,-2.0833719031,0.0310713925\H,6.8085511798,1.8249391927,0.18745
50403\H,4.5813435366,2.9035318752,0.2099099639\H,-4.1411260724,-2.2475
228626,-0.0445792887\H,-6.6187288824,-2.3796518079,-0.0682502544\H,-6.
8379100551,1.9049746237,0.0865777272\H,-4.3583073955,2.0224522597,0.10
97288476\H,1.8827498678,3.4333347229,0.2087346757\H,7.0196059938,-0.63
23539608,0.099306936\H,-7.9716628106,-0.3009131364,-0.0026153661\N,2.4
002421417,-0.9733256146,0.051716057\C,2.1827615053,-2.4140189687,-0.00
25598391\H,1.1146227637,-2.6066465222,-0.0177202951\H,2.6425764313,-2.
8280670976,-0.9044835218\H,2.6290513635,-2.8930133732,0.8736374399\\Ve
rsion=ES64L-G09RevD.01\State=1-A\HF=-989.7980352\RMSD=7.642e-09\RMSF=2
.117e-06\Dipole=0.8016562,0.1099884,0.0101152\Quadrupole=8.1819972,2.7

191482,-10.9011453,-2.2450893,0.0632867,0.4810649\PG=C01 [X(C20H14N4)]
\\@

Low frequencies --- -2.3091 -0.0013 0.0005 0.0011 3.6358 10.9311
Low frequencies --- 28.8740 39.5972 75.6019

11 cyclization on N(1)

1\1\GINC-GAUSIANDELL\FOpt\UB3LYP\6-311G(d,p)\C20H15N4(2)\PKASZYNSKI\23
-Jul-2023\0\\#P UB3LYP/6-311G(d,p) FOpt=tight freq(noraman, readiso) S
CF=Direct #P Geom=(NoDistance,NoAngle) fcheck\\C(8)-NMe-Ph(N1) GS befo
re cyclization onto N1\\0,2\N,0.2723420928,-0.185794026,-0.6474581303\
N,-1.7077942927,1.5194211036,0.2333358916\C,-1.9299528819,0.281262773,
-0.165035289\N,-0.9660689368,-0.5701148742,-0.6447611225\C,2.238506715
3,2.7937353196,0.1135806045\C,1.9832262853,1.4650867821,-0.200386657\C
,0.600650166,1.048000105,-0.195705238\C,-0.4267356215,1.9508849761,0.2
063889849\C,-0.1107103847,3.2866969776,0.5429976851\C,1.2019731741,3.6
825814967,0.4744509328\C,2.6924405456,-1.060612409,1.2335819205\C,3.00
78637359,-0.7501902347,-0.0748556651\C,3.4066021847,-1.8290578507,-0.8
784450697\C,3.468231471,-3.1172426714,-0.3541287797\C,3.1204556878,-3.
3703860365,0.9724095868\C,2.709790963,-2.3113978653,1.7941917223\C,-3.
3111086164,-0.2530180573,-0.1258112546\C,-3.5846789405,-1.5631494881,-
0.5429971691\C,-4.8855401887,-2.0538177222,-0.5009179313\C,-5.92653256
35,-1.2476731099,-0.0438108895\C,-5.6604823541,0.0565072783,0.37322692
78\C,-4.3628888117,0.5521075128,0.3332463195\H,-0.9107331702,3.9544154
653,0.8349062717\H,3.6346086561,-1.6490047613,-1.9236273985\H,3.161027
1061,-4.3785729801,1.3695422553\H,2.4323964546,-2.4805370351,2.8289562
26\H,-2.7695505634,-2.179965614,-0.8964222666\H,-5.0870905994,-3.06839
00187,-0.8258687197\H,-6.4668995086,0.6877113445,0.7295336363\H,-4.143
9471425,1.5625783865,0.6529146704\H,1.4680930901,4.7044223929,0.722139
2842\H,3.2537778221,3.1642610996,0.1124325537\H,3.7713117024,-3.936527
4784,-0.9958350687\H,-6.9397659023,-1.6328067073,-0.0122418529\N,2.988
583557,0.5829537965,-0.5869467301\C,4.2963409316,1.1362250445,-0.93706
26018\H,4.17499377,1.9557150592,-1.6477763681\H,4.847764279,1.50188075
64,-0.0591532442\H,4.8928380883,0.3568332702,-1.407341027\\Version=ES6
4L-G09RevD.01\State=2-A\HF=-990.2791707\S2=0.757442\S2-1=0.\S2A=0.7500
31\RMSD=5.228e-09\RMSF=1.436e-06\Dipole=0.9464608,0.3713947,0.0853773\
Quadrupole=5.8012891,2.8766592,-8.6779483,3.2564498,-3.8929477,0.36222
61\PG=C01 [X(C20H15N4)]\\@

Low frequencies --- -5.9901 -0.0004 0.0000 0.0002 4.6167 6.8904
Low frequencies --- 28.3327 31.9247 38.7280

11-TS cyclization on N(1)

1\1\GINC-GAUSIANDELL\FTS\UB3LYP\6-311G(d,p)\C20H15N4(2)\PKASZYNSKI\23-
Jul-2023\0\\#P UB3LYP/6-311G(d,p) Opt(QST3) geom(noangle, nodistance)
fcheck #P freq(noraman, readIso)\\MeNPh rad TS Cyclization on N1, befo
re TS\\0,2\N,0.2042395937,-0.1798333222,-0.670057325\N,-1.7519769075,1
.5015790231,0.3109429264\C,-1.9937965835,0.2999268569,-0.17456849\N,-1
.0378989903,-0.538232308,-0.7011566971\C,2.2179663156,2.6935591683,0.3
32711601\C,1.9354981397,1.4067405368,-0.0979907614\C,0.5529008392,1.01
86191517,-0.1334047297\C,-0.4613070042,1.9075628796,0.3240856668\C,-0.
121579448,3.2066073088,0.7644914814\C,1.1998091101,3.5797440968,0.7485
197739\C,2.1286617813,-1.3799877782,0.7413265163\C,3.1014800764,-0.759
389319,-0.0271414833\C,4.2687524617,-1.5225144059,-0.2309251537\C,4.37
9218014,-2.8116234221,0.2960841111\C,3.356083487,-3.3829606146,1.04271
10445\C,2.1844768472,-2.6395685061,1.2647988434\C,-3.3835140088,-0.210
0520288,-0.1876275674\C,-3.6747308639,-1.4844967786,-0.694423423\C,-4.
9842660644,-1.9532801595,-0.6979065938\C,-6.0160367339,-1.1606512838,-

0.1984037498\C,-5.732559155,0.1082607918,0.3069982251\C,-4.4264846254,
0.5820080392,0.313108155\H,-0.9123037452,3.8661074143,1.0981240505\H,5
.098696165,-1.120910923,-0.7972861164\H,3.452808546,-4.3848114712,1.44
52462626\H,1.3571634314,-3.0546811313,1.8307544838\H,-2.8670983118,-2.
0916072338,-1.08026606\H,-5.1997379831,-2.9402340089,-1.0915022496\H,-
6.5319709881,0.7286881536,0.6963033679\H,-4.1940874118,1.5649008833,0.
701721732\H,1.4842606837,4.5710166359,1.0834938686\H,3.2465731405,3.02
68087489,0.3811191492\H,5.2899695016,-3.3706624418,0.1129635345\H,-7.0
359559926,-1.528933801,-0.2025102956\N,2.9565855612,0.5466651968,-0.53
64767859\C,4.0916898824,1.1357987078,-1.2382387615\H,3.7808971994,2.07
58274937,-1.6892252023\H,4.953373554,1.3200931732,-0.5829598253\H,4.40
91174866,0.4699476774,-2.0436105233\\Version=ES64L-G09RevD.01\State=2-
A\HF=-990.2787932\S2=0.758654\S2-1=0.\S2A=0.750048\RMSD=6.026e-09\RMSF
=5.700e-06\Dipole=0.6856527,0.901378,0.0723147\Quadrupole=8.8214935,1.
8256336,-10.6471271,5.9851895,-4.090728,-0.073886\PG=C01 [X(C20H15N4)]
\\@

Low frequencies --- -34.7337 0.0002 0.0005 0.0006 2.4299 4.0271
Low frequencies --- 7.5706 28.1143 35.0877
****** 1 imaginary frequencies (negative Signs) ******

11 cyclization on C(7)

1\1\GINC-GAUSIANDELL\FOpt\UB3LYP\6-311G(d,p)\C20H15N4(2)\PKASZYNSKI\21
-Jul-2023\0\\#P UB3LYP/6-311G(d,p) FOpt=tight freq(noraman, readiso) S
CF=Direct #P Geom=(NoDistance,NoAngle) fcheck guess=check\\C(8)-NMe-Ph
(N1) GS before cyclization onto C7\\0,2\N,0.3515024996,-0.875829053,0.
284992745\N,2.0678824842,1.2737725051,0.0493867781\C,2.4580970991,0.01
43748838,-0.0038988046\N,1.6174146166,-1.069031435,0.0868483645\C,-1.9
931176928,1.9086260515,0.6049293369\C,-1.5400527358,0.5993211426,0.604
1636042\C,-0.1338355655,0.3880671155,0.3884908659\C,0.7473648081,1.498
697319,0.2300155705\C,0.2367718979,2.8161649631,0.2396001269\C,-1.1136
3517,2.9969707173,0.4150891037\C,-4.0063974299,0.4134854426,-0.8656939
294\C,-3.6431748618,-0.5223667463,0.0969513878\C,-4.5952904322,-1.5422
505523,0.313193924\C,-5.7940389322,-1.562543169,-0.4008627812\C,-6.101
3724737,-0.58604083,-1.3420584871\C,-5.1733905478,0.4433637195,-1.5746
701914\C,3.8959279803,-0.2906910671,-0.1821049611\C,4.3466921211,-1.61
7436548,-0.2327408149\C,5.7005738529,-1.8907267098,-0.3963336049\C,6.6
190015205,-0.8488515579,-0.511629242\C,6.1765929516,0.4731754266,-0.46
2875212\C,4.8253742477,0.752401765,-0.299416817\H,0.925408833,3.640787
0626,0.1078336822\H,-4.4013313036,-2.3249764568,1.0346764108\H,-7.0346
76208,-0.6158636947,-1.8922871237\H,-5.3752668669,1.2232837523,-2.3016
206055\H,3.6262441059,-2.4190642914,-0.1430714021\H,6.0394384975,-2.91
98954205,-0.4338571887\H,6.887145774,1.2871225456,-0.5530209848\H,4.47
02877152,1.7739091383,-0.2609111144\H,-1.5249016086,4.0000633302,0.432
5170857\H,-3.0425244416,2.1042145931,0.7786080955\H,-6.4978590422,-2.3
646605103,-0.2091081465\H,7.6739279872,-1.0649223285,-0.6391711694\N,-
2.4156432847,-0.4851067996,0.7667936734\C,-2.0961446611,-1.5595907066,
1.7061916575\H,-1.1559316829,-1.3392447723,2.1995647244\H,-2.879596869
,-1.633354854,2.4687380621\H,-1.9940691823,-2.5213249708,1.1973993819\
\Version=ES64L-G09RevD.01\State=2-A\HF=-990.2831405\S2=0.758234\S2-1=0
.\S2A=0.75004\RMSD=2.610e-09\RMSF=2.285e-06\Dipole=-0.3613409,0.459881
9,0.4532796\Quadrupole=6.3802187,0.927499,-7.3077176,-0.0390559,-1.528
9002,-2.5188631\PG=C01 [X(C20H15N4)]\\@

Low frequencies --- -6.4693 -2.8635 -0.0007 -0.0006 -0.0004 6.4146
Low frequencies --- 21.5799 34.9872 36.1897

11-TS cyclization on C(7)

```
1\1\GINC-GAUSIANDELL\FTS\UB3LYP\6-311G(d,p)\C20H15N4(2)\PKASZYNSKI\22-
 Jul-2023\0\\#P UB3LYP/6-311G(d,p) Opt(QST3) geom(noangle, nodistance)
 fcheck #P freq(noraman)\\MeNPh rad TS Cyclization on C7, before TS\\0,
 2\N,0.4669679859,-1.1213289134,0.364013348\N,1.9305141772,1.2090715544
 ,0.1672577779\C,2.4563447113,-0.0032066516,0.0498759922\N,1.7433466113
 ,-1.1679466019,0.1249328058\C,-2.1469076683,1.4206432234,0.9479698124\
 C,-1.5511107232,0.1471216274,0.803057985\C,-0.1482057505,0.07662025,0.
 5363435489\C,0.6103840445,1.2836546016,0.4011335791\C,-0.0324933,2.549
 302248,0.4854996075\C,-1.367992468,2.6035532881,0.7658063467\C,-3.8406
 600002,0.7051330343,-0.3489005122\C,-3.6312694362,-0.6174065563,0.0305
 220027\C,-4.6068545163,-1.562269366,-0.3182657863\C,-5.7434364706,-1.1
 427399964,-1.0134683982\C,-5.9336118815,0.1924907603,-1.3582949885\C,-
 4.9662407354,1.1460606531,-0.9962207203\C,3.9097695592,-0.1356568831,-
 0.190509904\C,4.5033816642,-1.3998718045,-0.3205842027\C,5.8708309424,
 -1.5113633449,-0.5466869672\C,6.662980858,-0.3685689475,-0.6455724722\
 C,6.0790249216,0.8915731581,-0.5169529877\C,4.7130291656,1.0094820583,
 -0.2913055113\H,0.572446651,3.437987748,0.3574697817\H,-4.4927741577,-
 2.6071135823,-0.0571526238\H,-6.8188887299,0.4972999113,-1.9053143202\
 H,-5.1040308689,2.1925618129,-1.2491570951\H,3.8806482366,-2.280756501
 7,-0.2429450819\H,6.3199057387,-2.4931917447,-0.6460612736\H,6.6905093
 452,1.7836536016,-0.5935314994\H,4.2491234258,1.9819984092,-0.19145475
 49\H,-1.8567478098,3.5632105748,0.8911840466\H,-3.0485738902,1.5041568
 347,1.5407276501\H,-6.4913910475,-1.8788144637,-1.2858679296\H,7.72909
 99709,-0.4588153071,-0.8218790294\N,-2.4306941704,-0.9205445473,0.7041
 023636\C,-2.1027793774,-2.3041103154,1.0288112677\H,-1.2206765737,-2.3
 298995237,1.6583230457\H,-2.9431176177,-2.7508047162,1.5676819186\H,-1
 .895848816,-2.893685582,0.1307091781\\Version=ES64L-G09RevD.01\State=2
 -A\HF=-990.2737031\S2=0.787904\S2-1=0.\S2A=0.75081\RMSD=4.194e-09\RMSF
 =4.514e-06\Dipole=-0.5878824,0.2852315,0.3615396\Quadrupole=5.6149567,
 2.6624704,-8.277427,1.0266293,-1.6484691,-0.2717257\PG=C01 [X(C20H15N4
 )]\\@

Low frequencies --- -340.1954  -0.0005  -0.0004  0.0004  2.6459  4.0433
 Low frequencies ---   11.1546   25.3316   38.0336
 ******    1 imaginary frequencies (negative Signs) ******
```

12

```
1\1\GINC-GAUSIANDELL\FOpt\UB3LYP\6-311G(d,p)\C20H15N4(2)\PKASZYNSKI\23
 -Jul-2023\0\\#P UB3LYP/6-311G(d,p) FOpt=tight freq(noraman, readiso) S
 CF=Direct #P Geom=(NoDistance,NoAngle) fcheck\\C(8)-NMe-Ph(N1) cyclize
 d onto C7\\0,2\N,0.6188147023,-1.2173713832,0.1784789173\N,1.806169603
 9,1.2710330617,-0.1066150429\C,2.4976032356,0.1062268842,-0.0057144795
 \N,1.9237078398,-1.1069580196,0.087783061\C,-2.3116038083,1.1763325835
 ,0.5699000212\C,-1.5373654735,-0.1009374068,0.3257443022\C,-0.14262455
 41,-0.0808945111,0.2017257685\C,0.4974843859,1.1946344623,-0.006425534
 \C,-0.3108051199,2.3904612707,-0.2209241825\C,-1.6354128363,2.38514694
 57,-0.0177249583\C,-3.7192320374,0.7790857427,0.1793275649\C,-3.732410
 1366,-0.6166754952,0.0291684214\C,-4.9080645345,-1.308445666,-0.245598
 3064\C,-6.0828292894,-0.5626245241,-0.3760276515\C,-6.0809163629,0.823
 5600419,-0.2321040506\C,-4.8902291267,1.5033427211,0.0569747745\C,3.97
 01534872,0.1624000375,-0.0388829518\C,4.7369521913,-1.0121401284,0.037
 3377627\C,6.1242892133,-0.9486978979,0.0072910866\C,6.7725777256,0.282
 4123903,-0.0978622779\C,6.0190586236,1.4529167319,-0.1732804101\C,4.63
 02359067,1.3965290093,-0.1445147069\H,0.2158759399,3.2714793387,-0.568
 554267\H,-4.9196128773,-2.3845556831,-0.3637285144\H,-7.0048539318,1.3
 784250891,-0.342259604\H,-4.8939065857,2.5812039812,0.178903078\H,4.22
 64422599,-1.9624607857,0.1186740387\H,6.7040851928,-1.8632498768,0.066
```

4384489\H,6.5156888709,2.4135218472,-0.2553921916\H,4.0382962803,2.300
2666278,-0.2046966162\H,-2.231588843,3.2747519839,-0.1899606949\H,-2.3
393527111,1.3446919489,1.6671579478\H,-7.011429096,-1.0763866565,-0.59
69552787\H,7.8557422576,0.3283237292,-0.1204316281\N,-2.4344745984,-1.
131747217,0.1710079164\C,-2.1240016991,-2.5367806144,-0.0616770889\H,-
1.0565875546,-2.6846054191,0.0754098809\H,-2.6797333653,-3.1584453531,
0.6459757409\H,-2.4067206549,-2.8227657906,-1.0803406355\\Version=ES64
L-G09RevD.01\State=2-A\HF=-990.3365253\S2=0.769728\S2-1=0.\S2A=0.75032
\RMSD=9.993e-09\RMSF=4.118e-07\Dipole=-1.7662692,0.3772973,0.061686\Qu
adrupole=6.4438447,2.7305302,-9.1743749,1.9543119,0.1665416,0.8217993\
PG=C01 [X(C20H15N4)]\\@

```
Low frequencies ---  -2.3352  -0.0005  -0.0004   0.0001   0.9103  5.5574
 Low frequencies ---   24.7233   39.7613   66.7685
```

7. References

(1) Rigaku Oxford Diffraction (2020). CrysAlis CCD, CrysAlis RED, CrysAlisPro. Version 1.171.40.84a Rigaku Oxford Diffraction, Abingdon, England.

(2) Sheldrick, G. M. SHELXT- Integrated space-group and crystal-structure determination, *Acta Cryst., Sect.* **2015**, *A71*, 3-8.

(3) Hübschle, C. B.; Sheldrick, G. M.; Dittrich, B. ShelXle: A Qt graphical user interface for SHELXL, *J. Appl. Cryst.* **2011**, *44*, 1281–1284.

(4) Sheldrick, G. M. SHELXT- Integrated space-group and crystal-structure determination, *Acta Cryst., Sect.* **2015**, *C71*, 3-8.

(5) Gaussian 09, Revision A.02, M. J. Frisch, G. W. Trucks, H. B. Schlegel, G. E. Scuseria, M. A. Robb, J. R. Cheeseman, G. Scalmani, V. Barone, B. Mennucci, G. A. Petersson, H. Nakatsuji, M. Caricato, X. Li, H. P. Hratchian, A. F. Izmaylov, J. Bloino, G. Zheng, J. L. Sonnenberg, M. Hada, M. Ehara, K. Toyota, R. Fukuda, J. Hasegawa, M. Ishida, T. Nakajima, Y. Honda, O. Kitao, H. Nakai, T. Vreven, J. A. Montgomery, Jr., J. E. Peralta, F. Ogliaro, M. Bearpark, J. J. Heyd, E. Brothers, K. N. Kudin, V. N. Staroverov, R. Kobayashi, J. Normand, K. Raghavachari, A. Rendell, J. C. Burant, S. S. Iyengar, J. Tomasi, M. Cossi, N. Rega, J. M. Millam, M. Klene, J. E. Knox, J. B. Cross, V. Bakken, C. Adamo, J. Jaramillo, R. Gomperts, R. E. Stratmann, O. Yazyev, A. J. Austin, R. Cammi, C. Pomelli, J. W. Ochterski, R. L. Martin, K. Morokuma, V. G. Zakrzewski, G. A. Voth, P. Salvador, J. J. Dannenberg, S. Dapprich, A. D. Daniels, O. Farkas, J. B. Foresman, J. V. Ortiz, J. Cioslowski, and D. J. Fox, Gaussian, Inc., Wallingford CT, 2009.

(6) Cossi, M.; Scalmani, G.; Rega, N.; Barone, V. New developments in the polarizable continuum model for quantum mechanical and classical calculations on molecules in solution, *J. Chem. Phys.* **2002**, *117*, 43–54.

(7) De Vleeschouwer, F.; Chankisjijev, A.; Yang, W.; Geerlings, P.; De Proft, F. Pushing the boundaries of intrinsically stable radicals: Inverse design using the thiadiazinyl radical as a template, *J. Org. Chem.* **2013**, *78*, 3151–3158.

(8) Cossi, M.; Scalmani, G.; Rega, N.; Barone, V. New developments in the polarizable continuum model for quantum mechanical and classical calculations on molecules in solution, *J. Chem. Phys.* **2002**, *117*, 43-54, and references therein.

(9) Stratmann, R. E.; Scuseria, G. E.; Frisch, M. J. An efficient implementation of time-dependent density-functional theory for the calculation of excitation energies of large molecules, *J. Chem. Phys.* **1998**, *109*, 8218–8224.
