# Supplementary figures and images for: Toward N-peri-Annulated Planar Blatter Radical through aza-Pschorr and Photocyclization
Source: J Org Chem. 2023 Nov 24;88(24):17197–205. doi: 10.1021/acs.joc.3c02051 (PMC10729016; doi:10.1021/acs.joc.3c02051)

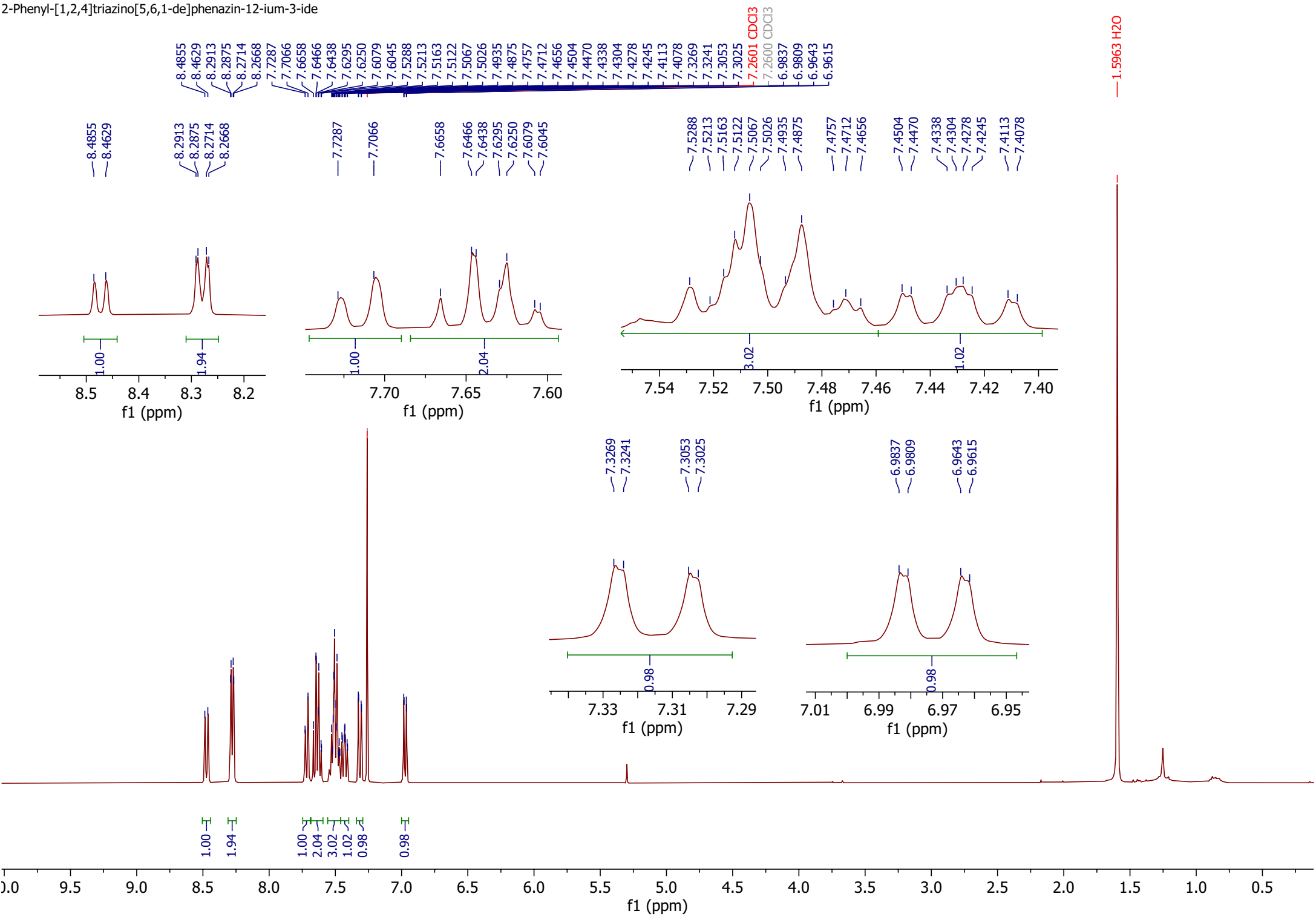

Supplement: Supplementary file 2 — jo3c02051_si_002.zip [file jo3c02051_si_002.zip › NMR N_Flat/2/1 H 2-Phenyl-[1,2,4]triazino[5,6,1-de]phenazin-12-ium-3-ide.pdf]

2-Phenyl-[1,2,4]triazino[5,6,1-de]phenazin-12-ium-3-ide

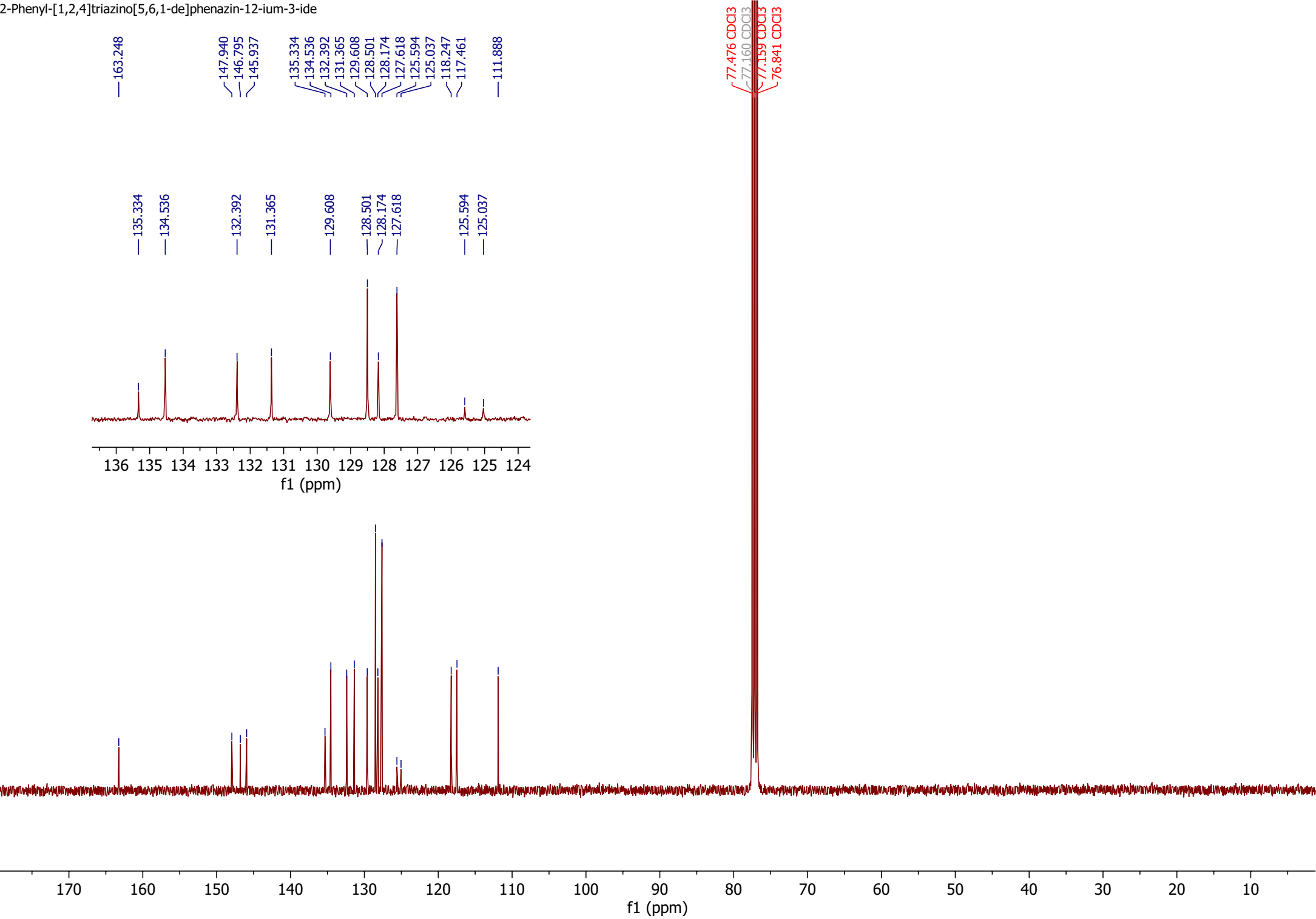

Supplement: Supplementary file 2 — jo3c02051_si_002.zip [file jo3c02051_si_002.zip › NMR N_Flat/2/13 C 2-Phenyl-[1,2,4]triazino[5,6,1-de]phenazin-12-ium-3-ide.pdf]

N'-(3-phenyl-1,2,4-benzotriazin-8-yl)benzene-1,2-diamine

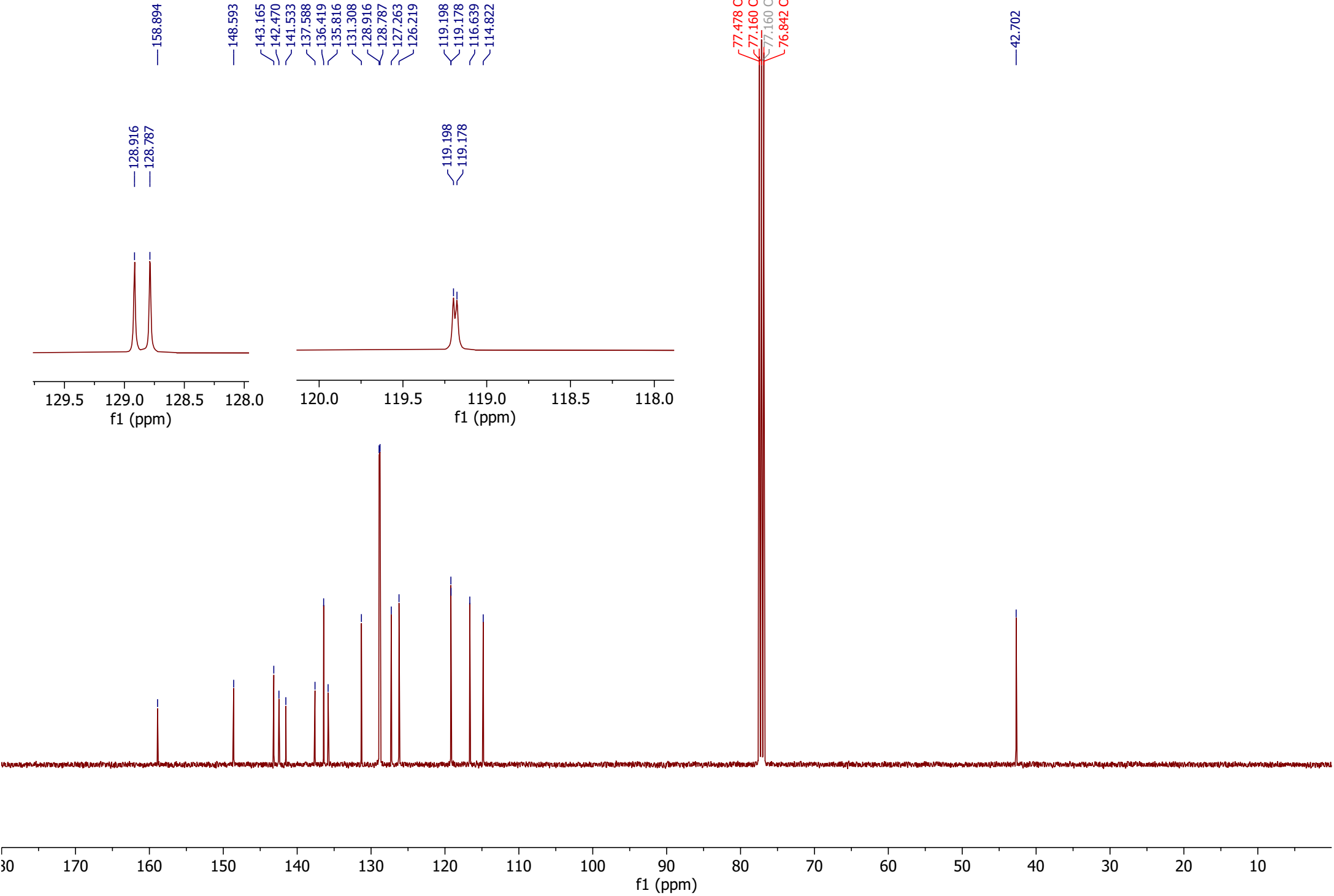

Supplement: Supplementary file 2 — jo3c02051_si_002.zip [file jo3c02051_si_002.zip › NMR N_Flat/3b/13 C N'-(3-phenyl-1,2,4-benzotriazin-8-yl)benzene-1,2-diamine.pdf]

N'-(3-phenyl-1,2,4-benzotriazin-8-yl)benzene-1,2-diamine

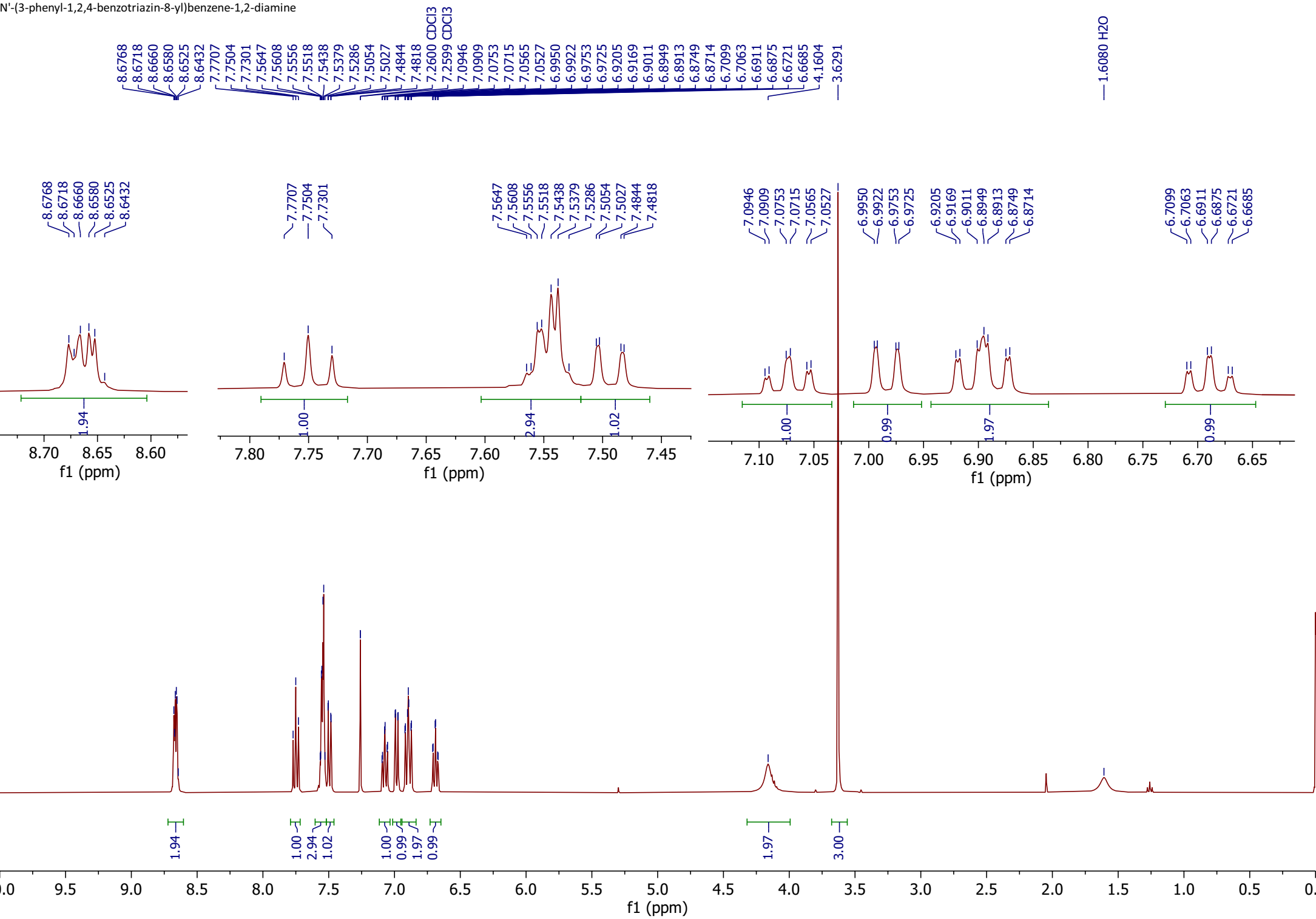

Supplement: Supplementary file 2 — jo3c02051_si_002.zip [file jo3c02051_si_002.zip › NMR N_Flat/3b/1H N'-(3-phenyl-1,2,4-benzotriazin-8-yl)benzene-1,2-diamine.pdf]

N-(2-Nitrophenyl)-3-phenylbenzo[e][1,2,4]triazin-8-amine

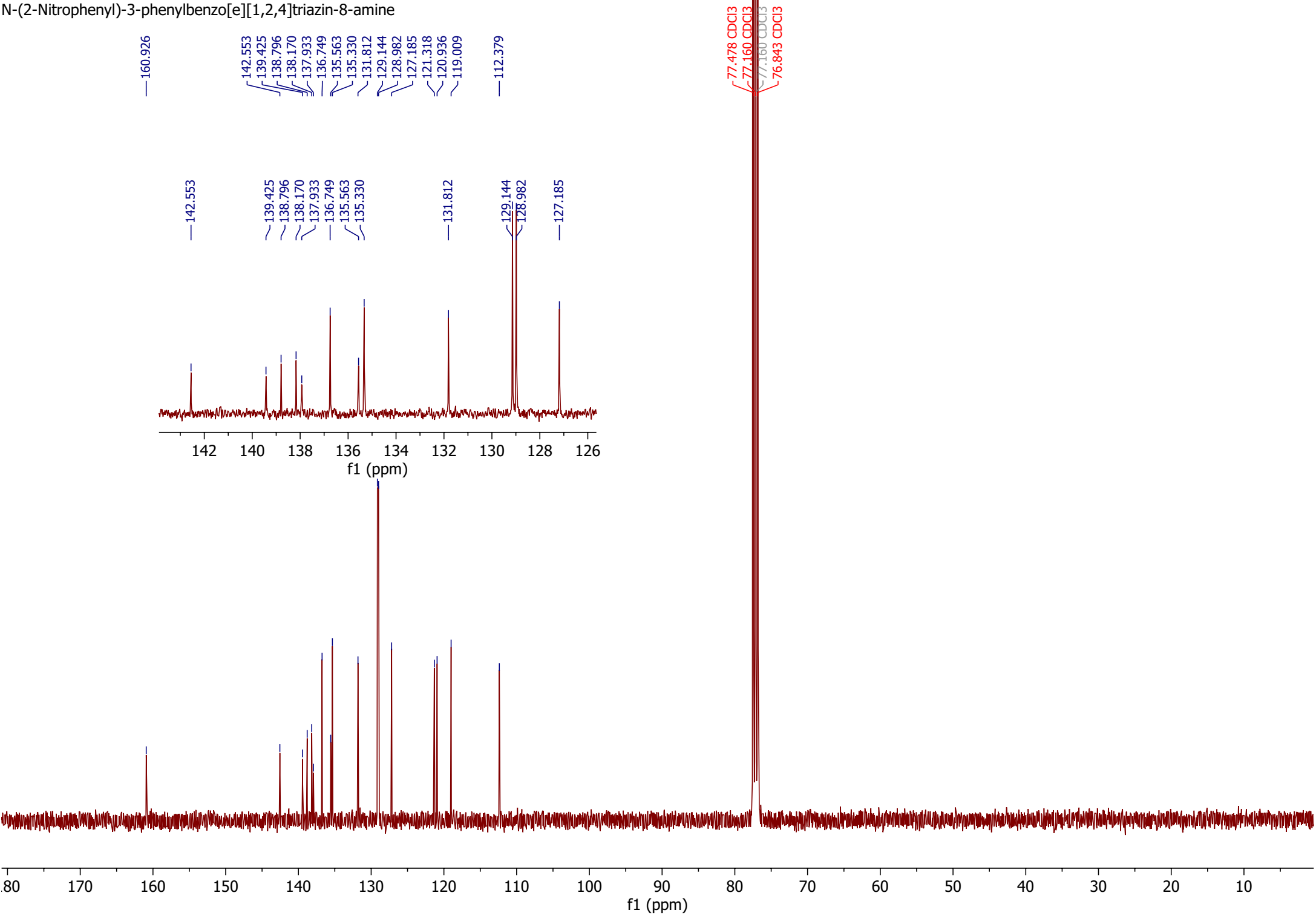

Supplement: Supplementary file 2 — jo3c02051_si_002.zip [file jo3c02051_si_002.zip › NMR N_Flat/4a/13 C N-(2-nitrophenyl)-3-phenyl-1,2,4-benzotriazin-8-amine.pdf]

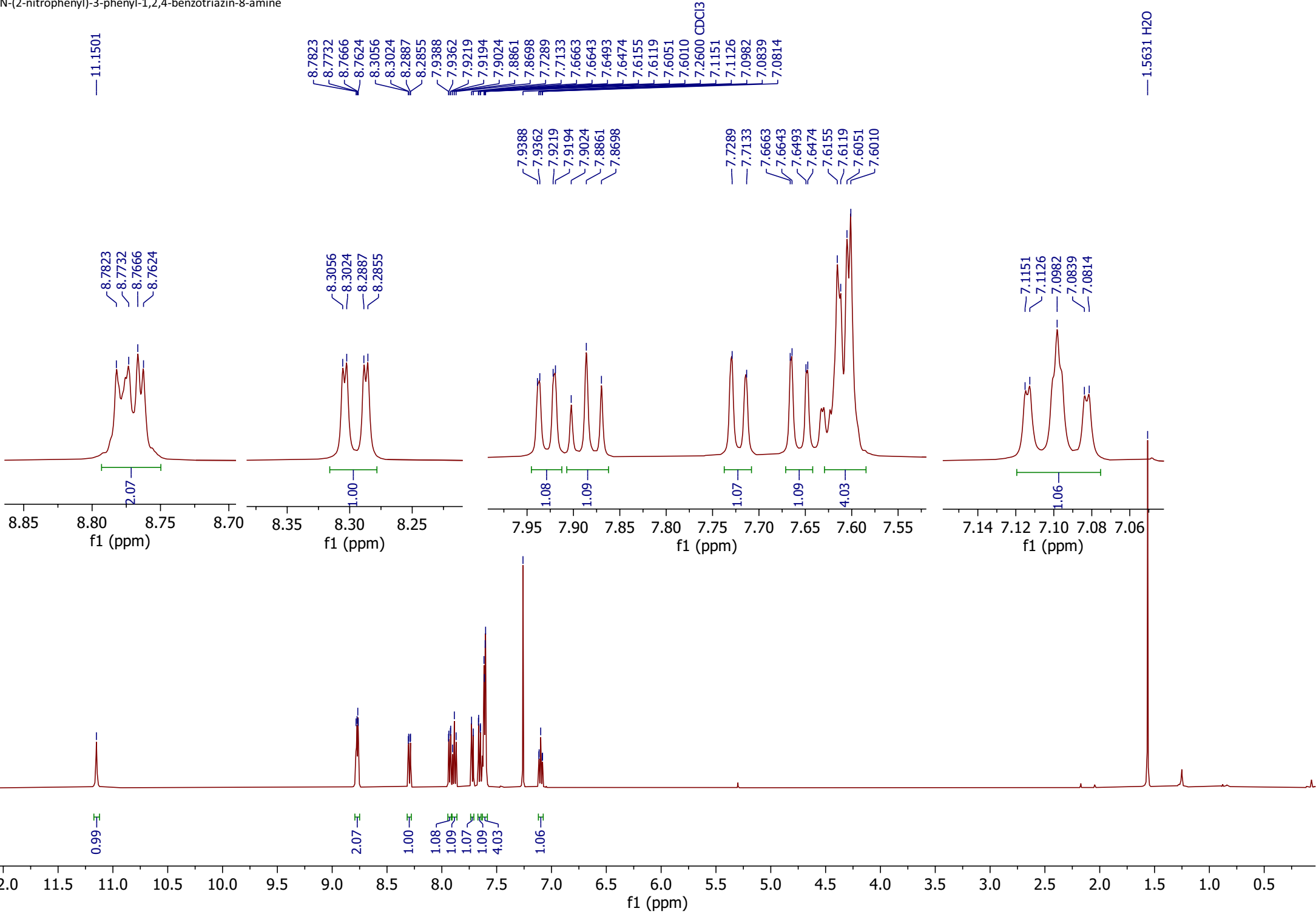

Supplement: Supplementary file 2 — jo3c02051_si_002.zip [file jo3c02051_si_002.zip › NMR N_Flat/4a/1H N-(2-nitrophenyl)-3-phenyl-1,2,4-benzotriazin-8-amine.pdf]

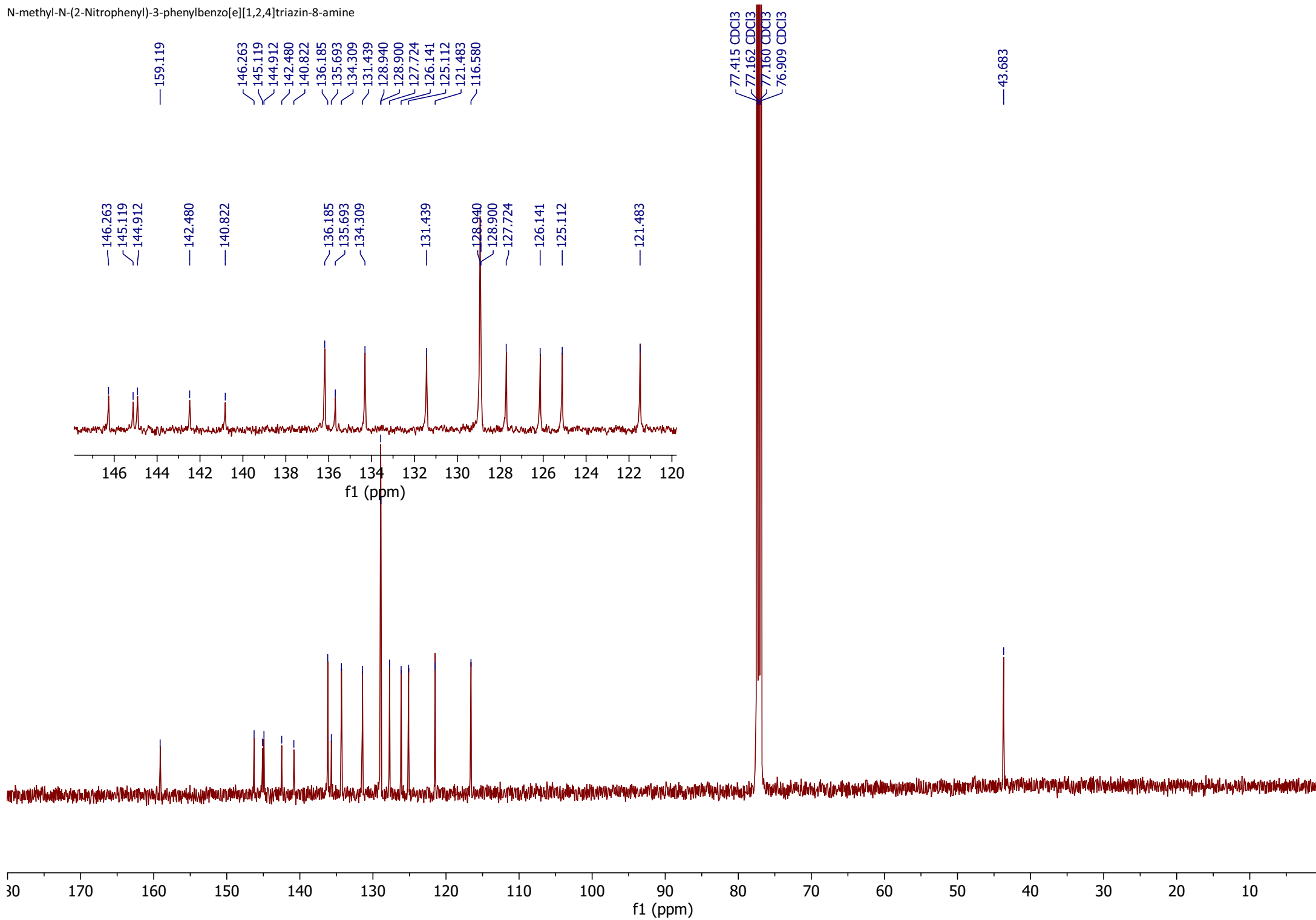

Supplement: Supplementary file 2 — jo3c02051_si_002.zip [file jo3c02051_si_002.zip › NMR N_Flat/4b/13 C N-methyl-N-(2-nitrophenyl)-3-phenyl-1,2,4-benzotriazin-8-amine.pdf]

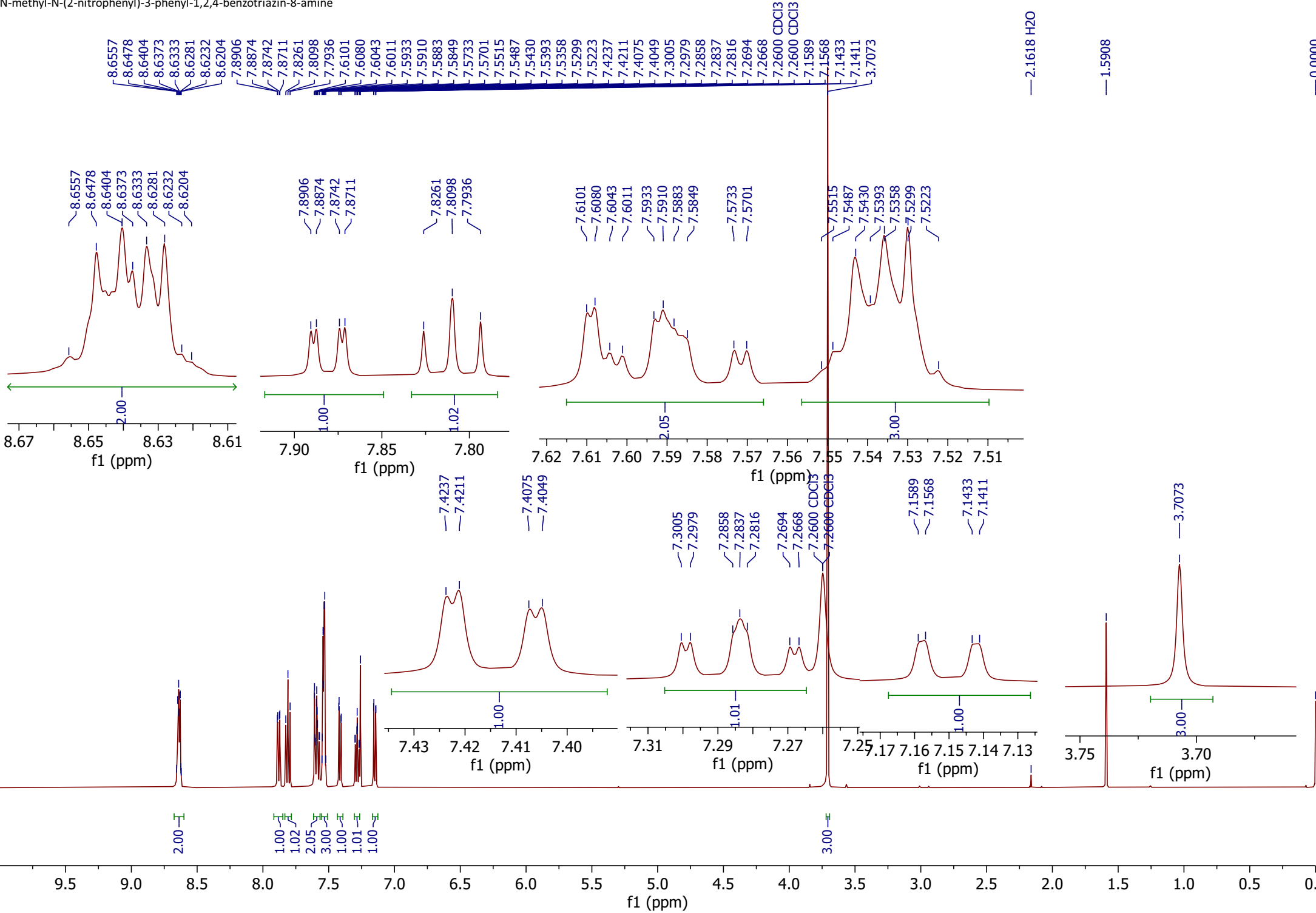

Supplement: Supplementary file 2 — jo3c02051_si_002.zip [file jo3c02051_si_002.zip › NMR N_Flat/4b/1H N-methyl-N-(2-nitrophenyl)-3-phenyl-1,2,4-benzotriazin-8-amine.pdf]
