# Supplementary material for: Toward N-peri-Annulated Planar Blatter Radical through aza-Pschorr and Photocyclization
Source: J Org Chem. 2023 Nov 24;88(24):17197–205. doi: 10.1021/acs.joc.3c02051 (PMC10729016; doi:10.1021/acs.joc.3c02051)

N-Methyl-N,3-diphenylbenzo[e][1,2,4]triazin-8-amine

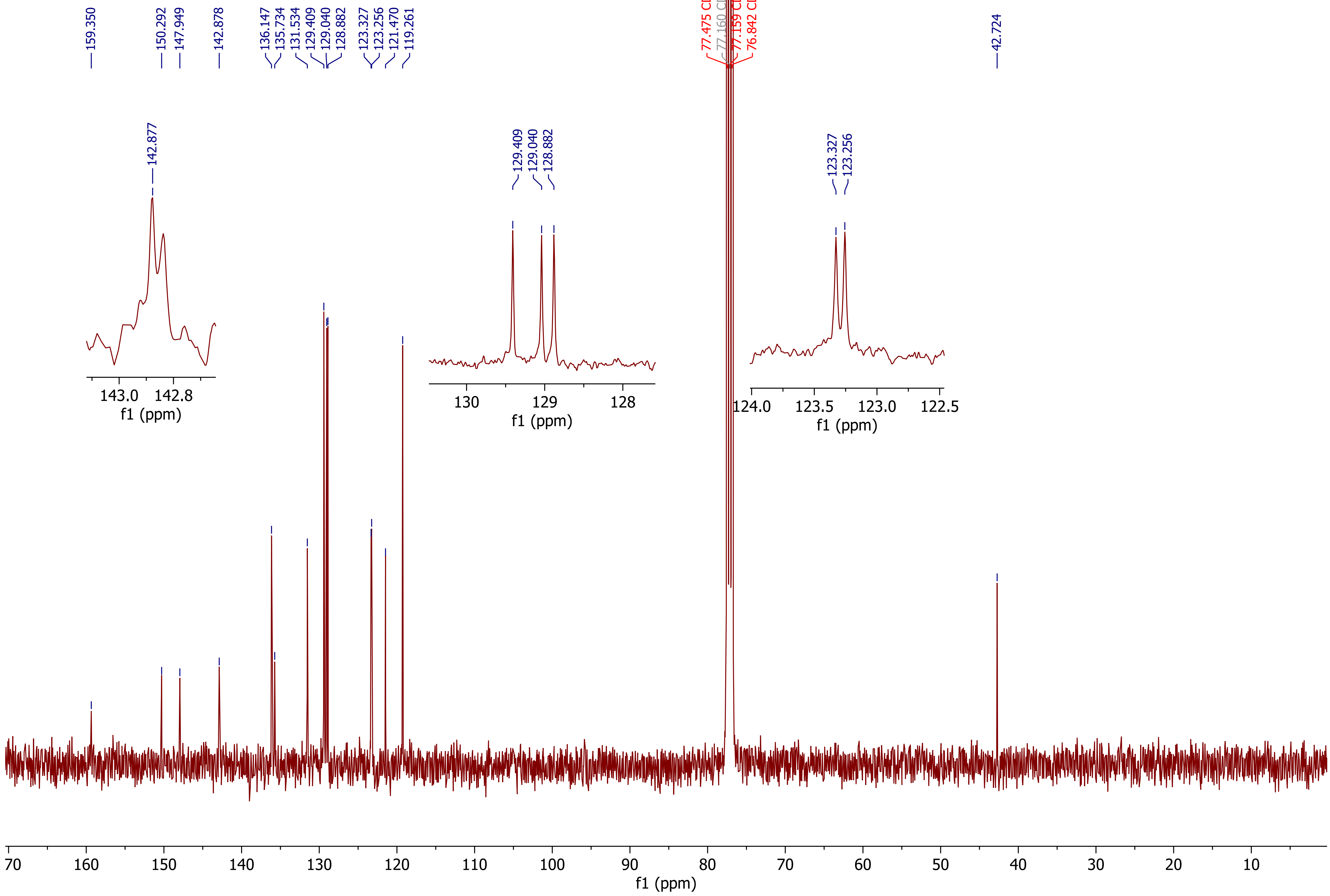

Supplement: Supplementary file 2 — jo3c02051_si_002.zip [file jo3c02051_si_002.zip › NMR N_Flat/5b/13C N-Methyl-N,3-diphenylbenzo[e][1,2,4]triazin-8-amine.pdf]
