# Supplementary material for: Toward N-peri-Annulated Planar Blatter Radical through aza-Pschorr and Photocyclization
Source: J Org Chem. 2023 Nov 24;88(24):17197–205. doi: 10.1021/acs.joc.3c02051 (PMC10729016; doi:10.1021/acs.joc.3c02051)

11-Methyl-3-phenyl-11H-[1,2,4]triazino[6,5-a]carbazole

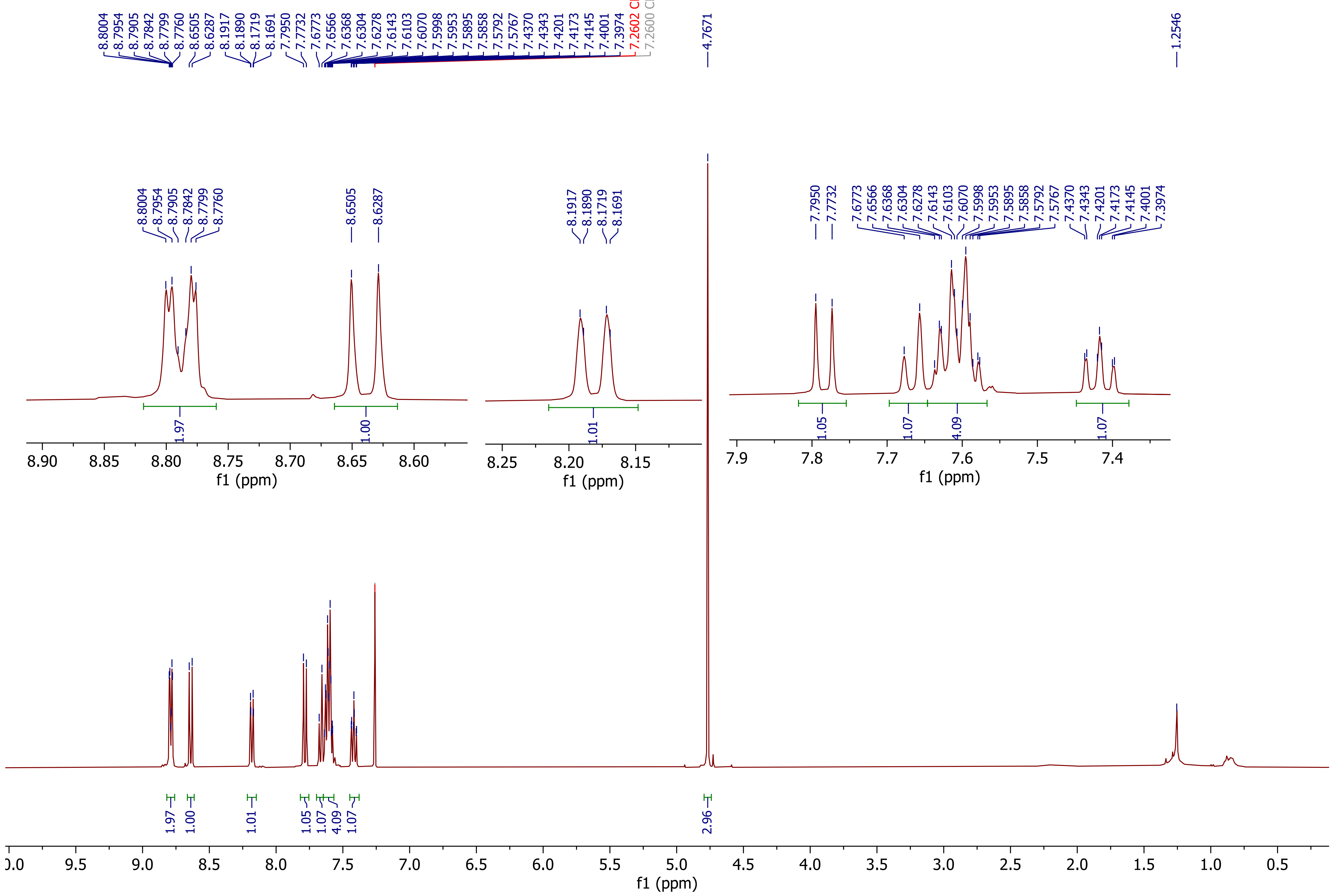

Supplement: Supplementary file 2 — jo3c02051_si_002.zip [file jo3c02051_si_002.zip › NMR N_Flat/9/1 H 11-Methyl-3-phenyl-11H-[1,2,4]triazino[6,5-a]carbazole.pdf]
